# Personalized relapse prediction in patients with major depressive disorder using digital biomarkers

**DOI:** 10.1038/s41598-023-44592-8

**Published:** 2023-10-30

**Authors:** Srinivasan Vairavan, Homa Rashidisabet, Qingqin S. Li, Seth Ness, Randall L. Morrison, Claudio N. Soares, Rudolf Uher, Benicio N. Frey, Raymond W. Lam, Sidney H. Kennedy, Madhukar Trivedi, Wayne C. Drevets, Vaibhav A. Narayan

**Affiliations:** 1 Janssen Research & Development, LLC, 1125 Trenton Harbourton Road, Titusville, NJ 08560 USA; 2 Department of Bioengineering, University of Illinois Chicago, Chicago, IL USA; 3 Department of Psychiatry, Queen’s University School of Medicine, Kingston, ON Canada; 4 Department of Psychiatry, Dalhousie University, Halifax, Canada; 5 Department of Psychiatry and Behavioural Neurosciences, McMaster University, Hamilton, ON, Canada; 6 Mood Disorders Program, St. Joseph’s Healthcare Hamilton, Hamilton, ON Canada; 7 Department of Psychiatry, University of British Columbia, Vancouver, Canada; 8 Centre for Depression and Suicide Studies, Unity Health Toronto, Toronto, Canada; 9 Krembil Neurosciences, University Health Network, Toronto, Canada; 10 Department of Psychiatry, University of Toronto, Toronto, Canada; 11 Peter O’Donnell Jr. Brain Institute and the Department of Psychiatry, UT Southwestern Medical Center, Dallas, TX USA; 12 Present Address: Davos Alzheimer’s Collaborative, Geneva, Switzerland

**Keywords:** Depression, Sleep disorders

## Abstract

Major depressive disorder (MDD) is a chronic illness wherein relapses contribute to significant patient morbidity and mortality. Near-term prediction of relapses in MDD patients has the potential to improve outcomes by helping implement a ‘predict and preempt’ paradigm in clinical care. In this study, we developed a novel personalized (N-of-1) encoder-decoder anomaly detection-based framework of combining anomalies in multivariate actigraphy features (passive) as triggers to utilize an active concurrent self-reported symptomatology questionnaire (core symptoms of depression and anxiety) to predict near-term relapse in MDD. The framework was evaluated on two independent longitudinal observational trials, characterized by regular bimonthly (every other month) in-person clinical assessments, weekly self-reported symptom assessments, and continuous activity monitoring data with two different wearable sensors for ≥ 1 year or until the first relapse episode. This combined passive-active relapse prediction framework achieved a balanced accuracy of ≥ 71%, false alarm rate of ≤ 2.3 alarm/patient/year with a median relapse detection time of 2–3 weeks in advance of clinical onset in both studies. The study results suggest that the proposed personalized N-of-1 prediction framework is generalizable and can help predict a majority of MDD relapses in an actionable time frame with relatively low patient and provider burden.

## Introduction

Major depressive disorder (MDD) is one of the leading causes of disability worldwide, with a lifetime prevalence of ~ 15% (> 300 million people worldwide), and is associated with significant morbidity and mortality^1^. The illness course in MDD is dynamic and variable, with episodes of relapses interspersed with periods of remission. Disease state transitions may occur faster than the time between clinic visits, and often leave the patient without needed intervention until the next scheduled clinic visit, or until an emergency arises due to a relapse. Currently, there are no individual patient-level indicators which have been shown to accurately quantify risk of near-term relapse in individual patients with MDD and the current guidance recommends visits every 1 to 6 months based on clinical judgment^2^.

In primary care and psychiatry settings, self-reported measures such as the 9-item Patient Health Questionnaire (PHQ-9) and the 16-item (9 domain) Quick Inventory of Depressive Symptomatology—Self-Report (QIDS-SR16)^3^ are commonly used to assess the presence and severity of depressive symptoms^4^. Although these two measures are relatively brief, efforts have been made to extract shorter versions (e.g., The Very Quick Inventory of Depressive Symptomatology [VQIDS-SR5]^4^), in order to further reduce the time burden on patients and clinicians while maintaining or improving the sensitivity of the original measures. In an effort to monitor individual patients with MDD for relapse risk, Judd et al.^2^ proposed using a set of 12-symptoms from the Symptom Checklist-90 assessment, to identify patients with substantial risk of relapse within the next 6 months using a mixed logistic regression model. In contrast to the periodic use of self-report measures or in-person assessments completed at sparse clinical visits, continuous passive data collection from smartphones and wearable sensors provide an unprecedented opportunity to derive insights on MDD symptom evolution and opportunities to predict near-term relapse, thus enabling proactive and preemptive care^5^. For example, sleep disturbance is a very common symptom in patients with MDD and up to 90% of individuals with MDD experience poor sleep quality during a depressive episode^6^. Wrist-worn actigraphy devices are widely accepted and commonly used to objectively assess sleep, wakefulness, and daily/circadian activity rhythms^7–10^. However, one of the major challenges in interpreting actigraphy-derived sleep measures (e.g., sleep duration) is the confounding influence of uncontrolled daily schedules, environmental conditions, and individual differences^11,12^.

Over the last two decades, the interdisciplinary field of “fractal physiology” has found that physiological outputs, such as motor activity and cardiac activity, exhibit fractal fluctuations (similar temporal, structural and statistical properties of the fluctuations at a wide range of time scale)^11–13^. One of the inherent characteristics of fractal fluctuations is that they are stable within individuals and sensitive to pathological conditions^11^. Fractal activity patterns persist under different environmental conditions, despite large variations in mean physical activity, and tend to degrade with aging and pathological states^14,15^. Moreover, many physiological outputs also exhibit a special class of complex processes termed “multifractal”, characterized by multiple co-existent dynamic processes and temporally local fluctuations captured by distinct scaling properties at different time scales^16,17^. Such multifractal processes can be exquisitely sensitive to changes in underlying behavioral or physiological states. Heath et al.^18^ first demonstrated changes in the multifractal properties of activity for a patient on the verge of a manic episode. In addition to fractal measures, measures from nonlinear dynamics such as sample entropy have been applied to actigraphy data to study randomness and chaos signatures in patients with depression and schizophrenia^19^.

Key challenges in building robust and generalizable relapse prediction models in MDD include class imbalance caused by relatively low prevalence of the relapse events and considerable intra and inter-individual variability in the active and passive features associated with depression severity^20,21^.

In the present study, we addressed the skewed distribution of classes and intra and inter-individual variability by considering relapse as unique events characterized by deviation from the patient’s normal behavior. To this end, we developed a personalized (N-of-1) Long Short-Term Memory Networks (LSTM) based Encoder-Decoder scheme for Anomaly Detection (EncDec-AD) using fractal and entropy activity features^22,23^ leveraging the longitudinal data collection (≥ 1 year). Once anomaly was detected in a patient’s actigraphy data (passive measure), symptom exacerbation was assessed by nearest self-reported questions (VQIDS-5, GAD-7) (active measure) and if and only if anomaly in actigraphy data was accompanied by self-reported symptom exacerbation, the flag for impending relapse was raised. Herein, we evaluated the performance of the proposed framework (‘Passive + Active’) and ‘Active’ only framework for a continuous prediction of relapses in patients with MDD. We believe that leveraging a personalized N-of-1 relapse prediction framework that combines passive and active data can predict relapses in individuals with MDD within a time frame that potentially supports earlier intervention leading to better outcomes.

## Methods

### Study design and data collection

The data from two prospective, multicenter, longitudinal, single-cohort, observational studies (OBSERVEMDD [NCT02489305] and CBN-WELL [NCT02934334]) were analyzed. The OBSERVEMDD and CBN-WELL studies were conducted from 15 December 2014 to 1 May 2018 and 31 May 2016 to 25 February 2019, respectively. These studies enrolled patients with recurrent MDD and the inclusion, exclusion criteria, and treatments for both studies are described briefly below, with additional details provided in the supplement (Supplement 1, OBSERVEMDD Protocol; Supplement 2, CBN-WELL Trial Protocol).

### Inclusion criteria

In OBSERVEMDD, adult patients aged 18–64 years were recruited if they met the criteria for nonpsychotic, recurrent MDD according to the Diagnostic and Statistical Manual of Mental Disorders, Fifth Edition (DSM-V) criteria and had experienced the onset of the current major depressive episode within the 1 year prior to screening, as confirmed using the MINI International Neuropsychiatric Interview [MINI—DSM-V]). Patients must have responded to and must be willing to continue an oral antidepressant treatment (within the past 3 months) and have a Montgomery-Asberg Depression Rating Scale (MADRS) score ≤ 14 at both the screening and baseline visits. In addition, patients endorsed willingness to complete self-reported assessments via a study-specific smartphone and to wear a wrist actigraphy device for the entire duration of the study. In CBN-WELL, the same inclusion criteria as described for the OBSERVEMDD study were followed except that the recency (within the past 3 months) of achieving remission in response to current antidepressant treatment was not required for inclusion (and patients achieving remission because of psychotherapy treatment could be eligible for enrollment as well) and that the patients were not required to continue pharmacotherapy throughout the study.

### Exclusion criteria

In both studies, volunteers were excluded if they met lifetime DSM-V criteria for MDD with psychotic features, bipolar disorder, schizophrenia, or schizoaffective disorder, or if they had manifested drug/alcohol use disorder of at least moderate severity within 6 months. Patients who had received vagal nerve stimulation, electroconvulsive therapy, transcranial magnetic stimulation, or deep brain stimulation were excluded from participation. Patients currently receiving stimulants, anticonvulsants, or mood stabilizers were excluded. Additionally, women who were pregnant or planning to become pregnant during screening were excluded.

### Treatment

In both studies, patients were allowed to continue their antidepressant treatment regimen per their treating physician/clinician during the study. Adjustment to the antidepressant treatment regimen (if applicable) by means of dose changes, switches in the antidepressant used, augmentation of the current antidepressant, or combining two antidepressants, was allowed as clinically indicated, irrespective of the relapse status, based on the judgment of the treating physician. No treatment type or dose was initiated, specified, or changed based on this observational study protocol.

The study protocols/amendments were approved by the Institutional Review Board (IRB) or an independent research ethics board at each study center. The studies were conducted in accordance with the ethical principles originating in the Declaration of Helsinki, the International Conference on Harmonization (ICH) Good Clinical Practice guidelines, and applicable regulatory requirements and in compliance with the protocol. All patients provided written informed consent prior to study participation. The OBSERVEMDD study was approved by the respective local or central IRBs overseeing the clinical sites participating in the study. These included the following IRBs: Southern Illinois University School of Medicine; Behavioral Research Specialists, LLC; University of Iowa, Dept of Psychiatry, College of Medicine; Southwest Family Medicine Associates; Weill Cornell Medical College; CNSHealth Care; North County Clinical Research; University of Kansas School of Medicine, Psychiatry and Behavioral Sciences; Medical University of South Carolina; Psychiatry and Health Behavior/Medical College of Georgia; University of Michigan Medical School; University of Cincinnati, Dept of Psychiatry and Behavioral Neuroscience; Sharp Mesa Vista Hospital; Psychiatric & Behavioral Solutions, LLC; UMass Medical School; Stanford Department of Psychiatry; Baylor College of Medicine/Harris Health System; The University of Alabama at Birmingham; California Neuroscience Research Medical Group Inc; Uptown Research Institute, LLC; Perelman School of Medicine of the University of Pennsylvania; Rush University Medical Center, Treatment Research Center, Dept of Psychiatry; Hartford Hospital; Olympian Clinical Research; and Psychiatric Medicine Associates, LLC.

The CBN-WELL study was approved by the University Health Network Research Ethics Board; The University of British Columbia Office of Research Ethics; Conjoint Health Research Ethics Board Research Services Office; Hamilton Integrated Research Ethics Board; and Queen's University Health Sciences and Affiliated Teaching Hospitals Research Ethics Board (HSREB).

Both studies consisted of a screening phase up to 2 weeks, followed by an observational phase of variable duration with regular clinic visits (bimonthly: every other month) (Supplement 3, Fig. S1). These patients were longitudinally followed for ≥ 1 year or until the first relapse episode. At baseline, patients completed assessments corresponding to MDD symptoms, anxiety, anhedonia, sleep disturbance, energy/motivation, functional/disability, health-related quality of life, mood-related cognition, pain, stress/resilience, and healthcare utilization (Supplements 1 and 2). All participants were given a study-specific smartphone, which they used to complete the above-mentioned assessments at and between clinic visits at varying frequencies ranging from weekly to bimonthly (Supplements 1 and 2). Motor activity and sleep parameters were objectively captured using actigraphy via a wrist-worn device (OBSERVEMDD: Actiwatch [Philips Respironics, USA]; CBN-WELL: ActiGraph GT9X Link [ActiGraph, USA]) throughout the study period. The participants received reminders to complete questionnaires using the provided smartphones. During clinic visits, clinicians also reminded patients of the importance of completing self-report measures between visits.

**Figure 1:**
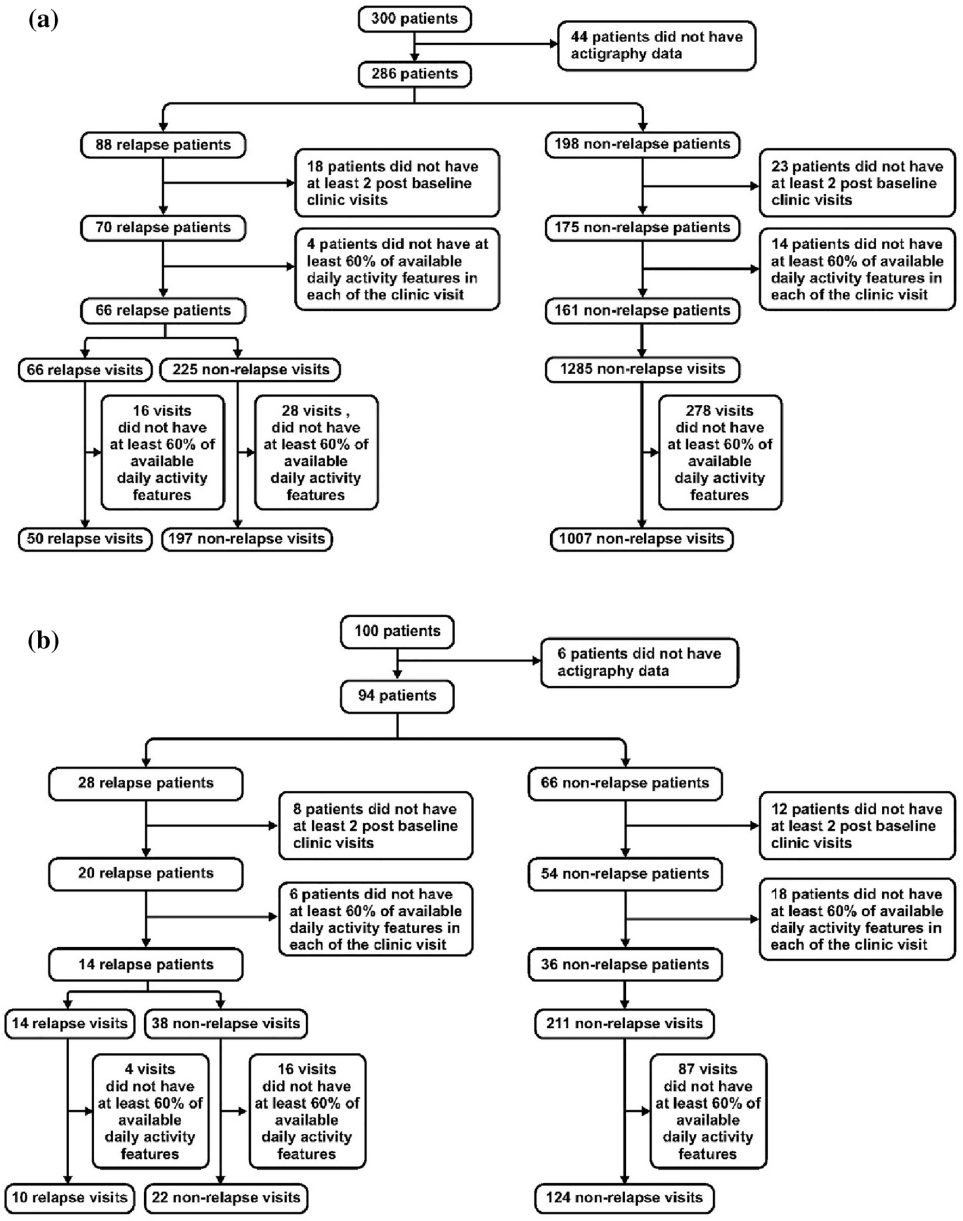
Patient selection (**a**) OBSERVEMDD and (**b**) CBN-WELL.

Throughout the study period, the patients were evaluated by their physician in bimonthly outpatient clinic visits, during which treatment and changes to the treatment regimen were determined per the clinician’s judgment (without any restriction imposed by study participation). Each clinic visit was labelled as a relapse or non-relapse visit based on pre-specified clinical relapse criteria as shown in Table 1. As clinic visits occurred at bimonthly intervals, the relapse versus non-relapse status at the current clinic visit was assumed to have been continuous since the time of the previous clinic visit. The participants included in the final analysis are shown in Fig. 1.

**Table 1:** Relapse definition.

Relapse definitions (any of the following)
MADRS^37^ total score ≥ 22 at a study visit, and a symptom worsening was confirmed during a subsequent Relapse Verification visit over an approximately 1- to 2-week interval If a patient received a MADRS rating ≥ 22 at a study visit (scheduled or unscheduled), an additional visit (i.e., the Relapse Verification visit) was scheduled within 1 to 2 weeks • Patients whose MADRS rating was ≥ 22 at the relapse verification visit were considered to have relapsed OR • CGI-S^38^ change from baseline ≥ 2 at the relapse verification visit or medication changes happened during + or − 14 days from the study visit were considered to have relapsed
Hospitalization for worsening of depression
Suicidal ideation with intent, or suicidal behavior
Investigator’s decision (crisis event at the therapy visit, change of medication due to worsening of depression)

CGI-S, the clinical global impression–severity scale; MADRS, montgomery and asberg depression rating scale; crisis event–increased anxiety and depression, feeling sad and crying every day.

In OBSERVEMDD, 88 patients had a relapse, and 198 patients completed prospective follow-up without experiencing a relapse. For each patient with a relapse who was included in the analysis, the data acquired prior to the first relapse episode were analyzed. Activity data were available from 197 relapse-free visits among patients who were going to eventually experience a relapse and 1007 visits from patients who remained stable throughout the follow-up duration. The number of available relapse clinic visits with available activity data was 50 leading to a total number of 1254 (1204 non-relapse and 50 relapse) available clinic visits for evaluation.

In CBN-WELL study, 28 patients had a relapse, and 66 patients completed prospective follow-up without experiencing a relapse episode. Activity data were available from 22 relapse-free visits among patients who were going to eventually experience a relapse and 124 visits from patients who remained stable throughout the follow-up duration. The number of available relapse clinic visits with available activity data was 10 leading to a total number of 156 (146 non-relapse and 10 relapse) available clinic visits for evaluation. Additionally, in both studies, all the evaluated patients had at least 2 months period of continuous activity data with no relapses which were used for the initial training of the N-of-1 model.

The relapses were defined as shown in Table 1 and the distribution of the different relapse criteria is shown in Supplement 3, Table S1. For relapse identified by MADRS, the first instance when the MADRS ≥ 22 (with sustained symptom worsening observed in the relapse verification visit) was considered as the relapse onset time. However, for other relapse categories, the onset time corresponded to the time the clinicians observed the relapse or recurrence. For each non-relapse patient considered in the analysis, the data acquired through the last available clinic visit were analyzed.

### Personalized (N-of-1) relapse prediction model framework

**Figure 2:**
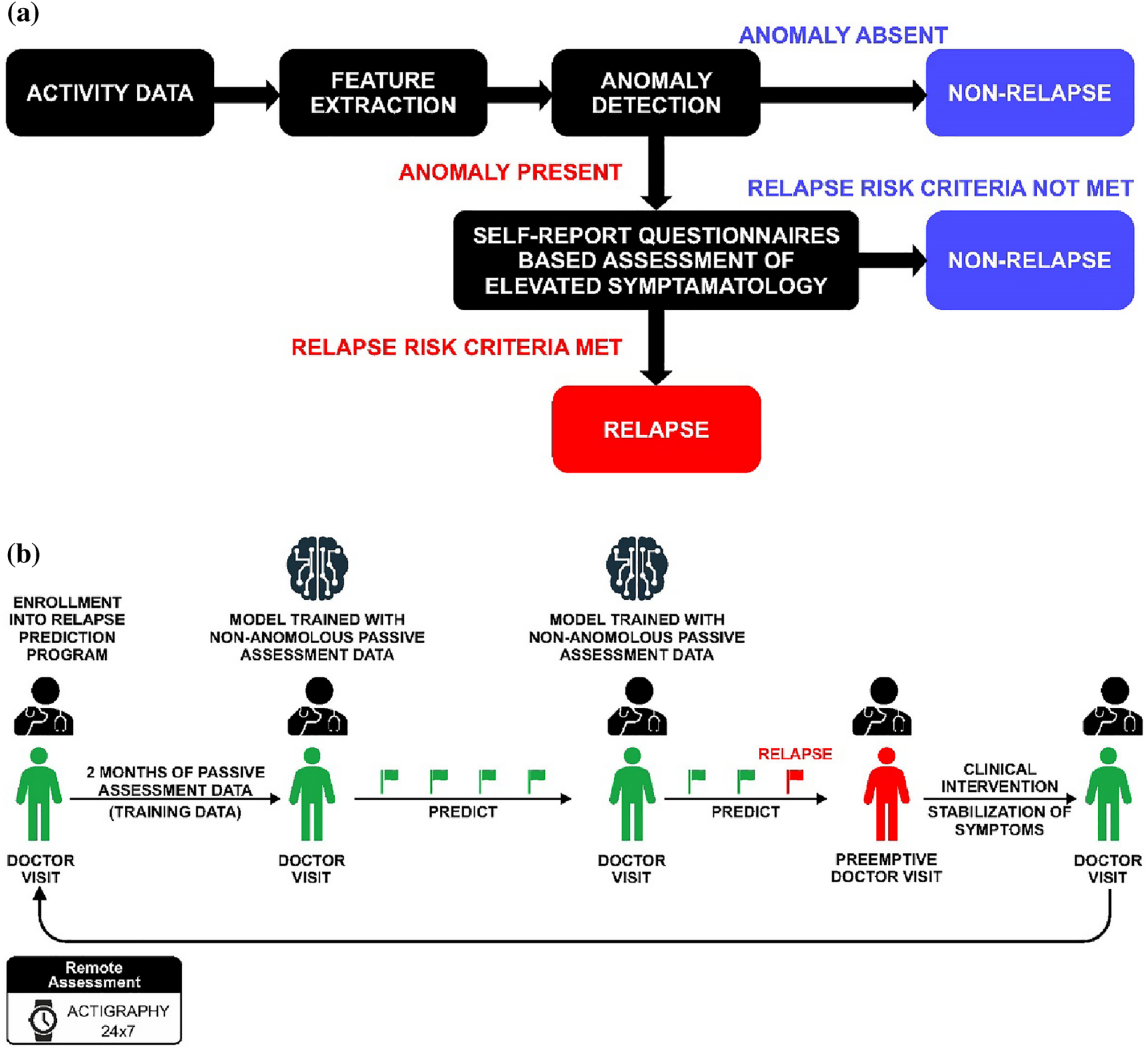
(**a**) Personalized relapse prediction framework and (**b**) Proposed deployment workflow of the personalized relapse prediction model. (**a**) Relapse risk criteria, elevated self-reported symptomatology assessments (core symptoms of depression (VQIDS-SR5) and anxiety (GAD-7)) (b) Model trained with non-anomalous passive assessment data, LSTM EncDec-AD; Flag, represents the personalized framework’s prediction of relapse status (green flag → non-relapse, red flag → relapse); Preemptive doctor visit, a clinic visit scheduled preemptively based on the personalized framework’s prediction of relapse in prospective deployment.

The proposed personalized relapse prediction framework is shown in Fig. 2a. The personalized framework referred to as “Passive + Active” was evaluated in patients with MDD through assessment of self-reported symptoms (measured weekly) and monitoring of continuous activity via an actigraphy device for ≥ 1 year or until the first relapse episode. The different stages of the framework are discussed in the sections below.

### Feature extraction

The motor activity was collected continuously using an activity monitor (Philips Actiwatch, and ActiGraph GT9X Link) worn on the nondominant wrist. The device measures acceleration in the direction parallel to the face of the device with a continuous sampling of 32 Hz and 30 Hz respectively. With a built-in data processor, the raw acceleration data were integrated into proprietary counts in 15-s epochs and 1-min epoch activity date, respectively, that reflect the movement amplitude. To have a similar resolution across devices, a minute resolution activity counts data stream was created by summing the 15-s epoch within each minute resulting in 1440 activity counts data points per day.

All actigraphy features were computed for each day with the last 7 days of continuous activity counts, with a minimum requirement of at least 2 days of continuous activity counts for computing the fractal features. The activity counts were further subjected to signal quality checks^11,24^ to detect isolation of outlying spikes with amplitude varying at least 10 standard deviations (SD) from the global mean levels and sequence of zeros with duration > 60 min.

The identified data points or segments were labelled as gaps and handled appropriately in feature computation. The different features computed were:

#### Detrended fluctuation analysis

Detrended fluctuation analysis (DFA) examines the multiscale correlations of activity fluctuations at multiple time scales quantifying fractal regulation which reflects the complexity of physiological control^24^. In this study, DFA was used to compute the scaling characteristics of motor activity fluctuations over a range of time scales from minutes to hours. A detailed description of DFA computation is provided elsewhere^11,14,15,24^. The DFA provides a fluctuation amplitude $F\left(n\right)$ as a function of time scales $n$. For long range-correlated data $F\left(n\right)$ follows a power law $F\left(n\right) \sim {n}^{\alpha }$, where scaling or fluctuation exponent ($\alpha$) quantifies the multiscale correlations as follows: $\alpha =$ 0.5 signifies no correlation in the fluctuation (“white noise”); $\alpha >$ 0.5 signifies positive correlations (i.e., large values are more likely to be followed by large values and vice versa) in the fluctuation; $\alpha <$ 0.5 signifies negative correlations (i.e., large values are more likely to be followed by small values and vice versa) in the fluctuation. Many physiological outputs under healthy conditions exhibit fluctuation exponent ($\alpha )$ values close to 1.0, putatively reflecting their regulation by complex underlying control mechanisms^14,25,26^.

A second order polynomial function was used to detrend the data to eliminate the effect of possible linear trends in the original data^11,14,15^. To ensure reliable estimation of $F\left(n\right)$ at a time scale $n$, the most recent continuous days of activity (at least 2 consecutive days) with no gaps > 72 min (5% of 1440 min of activity counts in a day) was used for each day. Fluctuation exponent ($\alpha$)^11,14,15^, was computed at two different time scales as ${\alpha }_{1}$ for 10 min (i.e., 10 data points with epoch length of 1 min) to 90 min and ${\alpha }_{2}$ during 120 min to 600 min, respectively to capture the distinct regions of activity dynamics. A reduction in fluctuation exponent, ${\alpha }_{1}$, and ${\alpha }_{2}$ (from values of 1 towards 0.5) reflects reduced complexity in the underlying dynamics. A reduced ${\alpha }_{1}$ has been associated with a dysregulation in higher brain activities including mood and cognitive function^11^and a reduced ${\alpha }_{2}$ has been associated with a circadian dysfunction^11^.

#### Multifractal detrended fluctuation analysis (MFDFA)

Many physiological time series, in addition to exhibiting long range correlations, also exhibit a special class of complex processes, namely, multifractal which require many exponents to characterize their scaling properties^16,18,27,28^. In Kantelhardt et al.^17^, the DFA has been modified to characterize q-order moments using which multifractal spectrum is calculated. The fluctuation functions obtained for higher moments (q) followed a power law: ${F}_{q}(s)\sim {s}^{h(q)}$, where $h(q)$ was defined as the generalized Hurst exponent and q corresponds to different moments. A monofractal time series exhibits equal q-order Hurst exponents while the multifractal time series exhibits q-order Hurst exponents that were significantly diversified indicating the local periods of small and large fluctuations^27^. A detailed description of MFDFA computation is found elsewhere^17,27,29^. The values of q varied from -5 to 5 in increments of 0.1 and as in DFA, $s$ varied from 10 to 600 min. A reduction in the width of the multifractal spectrum reflects reduced complexity in the underlying dynamics^16^.

#### Sample entropy

To quantify complexity of activity time series, a modified version of Sample Entropy (SaEn)^30^ with the time delay ($\delta$) was used. As the activity time series exhibit long range correlations, SaEn was applied to z-normalized increment of the data (first difference, i.e., $x\left(t+1\right)-x\left(t\right))$, the $\delta$ was set to unity as it becomes an anti-correlated process. The dimension of the state space (m) was set to 2 and radius (r) was set as 20% of the SD of the z-normalized increment of the data.

Each day of activity data was partitioned evenly into four epochs: morning (6 a.m. to 12 p.m.), afternoon (12 p.m. to 6 p.m.), evening (6 p.m. to 12 a.m.), and night (12 a.m. to 6 a.m.). SaEn was calculated for each day and for each epoch as the median SaEn value across the last 7 days of activity counts (without any identified gaps). A reduction in sample entropy indicates decreased complexity in underlying dynamics.

**Figure 3:**
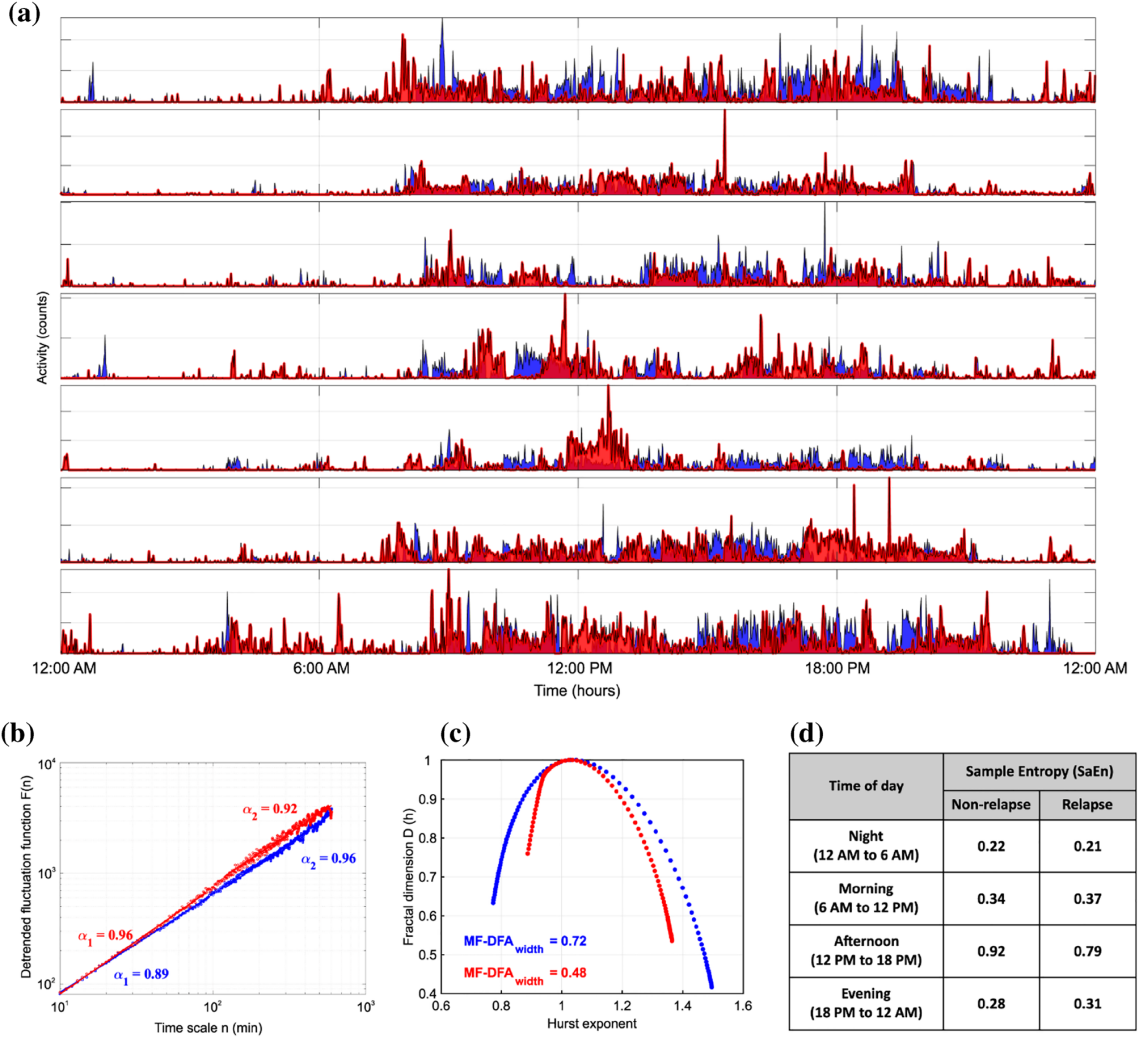
Extraction of entropy and fractal features from activity data corresponding to a week for a relapse patient. The blue lines correspond to non-relapse sections and red lines correspond to relapse sections. (**a**) Representative motor activity data (counts) corresponding to a non-relapse and relapse clinic visit for a patient. (**b**) DFA results for the non-relapse and relapse sections. The DFA fluctuation function $F(n)$ at different time $n$ are plotted in a log–log scale. $F(n)$ is fitted using a linear polynomial in two regions, 10 min to 90 min, and 120 to 600 min. The slopes of the fitting lines in the two regions are denoted by ${\alpha }_{1}$ and ${\alpha }_{2}$, respectively. For better visualization and comparison of the two signals, *F(n)* for relapse sections has been vertically shifted. (c) MFDFA results for non-relapse and relapse sections. MFDFA width calculated from the multifractal spectrum is shown for non-relapse and relapse sections. (d) Sample Entropy (SaEn) values for non-relapse and relapse sections. DFA, detrended fluctuation analysis; MF-DFA, multifractal detrended fluctuation analysis.

The fractal and entropy features extracted for representative relapse and non-relapse sections of a relapse patient are shown in Fig. 3.

The clinical relevance, risk factors of relapse and inter-individual variability of actigraphy features are discussed in Supplement 3 (Section V). To quantify the clinical relevance and identify the risk factors of relapse, the association between actigraphy features and depression outcome (relapse and symptom severity [MADRS and VQIDS-SR5]) was assessed using linear mixed effect models (logistic regression models for relapse and linear models for symptom severity) in both studies. The average marginal effect (AME) was used to quantify the effect of actigraphy features on depression. A marginal effect is the partial derivative of the regression equation with respect to each variable in the model for each unit in the data and AME is the mean of these partial derivatives. For relapse, the AME represents the percentage points increase in the probability of relapse per SD difference in the actigraphy feature. For depression severity, the AME represents the increase in MADRS or VQIDS-SR5 total score per SD difference in the actigraphy feature.

To quantify the inter-individual variability of actigraphy features and their association with core symptoms of depression, Spearman correlation was computed between actigraphy features and VQIDS-SR5 collected every week for each patient. The AME and inter-individual variability analysis are explained in detail in Supplement 3 (Section V).

### Anomaly detection

A LSTM EncDec-AD, proposed by Malhotra et al.^22,23^, was used to predict anomalies in the daily multi-features timeseries derived from activity data. Supplement 3, Fig. S2 shows the schematics of EncDec-AD architecture and Table S2 shows the LSTM EncDec model parameters. The LSTM EncDec-AD was trained only on the non-relapse daily multi-features timeseries with target time series being the input. The missing data in the multi-features timeseries were imputed with zeros. The reconstruction error at any future time instance was used to compute the likelihood of anomaly at that time point. A detailed description of LSTM EncDec-AD and its implementation can be found elsewhere^22,23^. The error vectors for time point ${t}_{i}$ is represented as ${e}^{(i)}$= $\left|{x}^{(i)}-{\widehat{x}}^{(i)}\right|$. The error vectors corresponding to non-relapse sections were used to compute the $\mu$ and $\Sigma$ of a Normal distribution $\mathcal{N} (\mu ,\Sigma )$ using Maximum Likelihood Estimation. An anomaly score or the Mahalanobis distance^31^, ${a}^{(i)}$ = $\sqrt{{{(e}^{(i)}-\mu )}^{T}{\Sigma }^{-1}{(e}^{(i)}-\mu )}$ was computed from the error vector corresponding to future time instance. The model has been trained continuously on non-relapse sections in 14-day segments on the z-normalized multivariate actigraphy daily features and produced the anomaly scores on unseen test data corresponding to the subsequent clinic visit.

#### Adaptive anomalous instances detection using dynamic thresholds

In the next step, instances of anomalies present in the anomaly score were detected using a modified version of the unsupervised anomaly scoring algorithm proposed by Hundman et al.^32^. The details of the different steps involved in the procedure are provided in Supplement 3.

### Assessment of elevated symptomatology with self-report questionnaires (relapse risk criteria)

The anomalous instances were determined in the test data in each 14-days block advancing every day. Each day was labelled as anomaly if there were anomalous instances identified within the last 7 days. This was done to align actigraphy-derived anomalous instances with self-report symptomatology questionnaires which have a recall period encompassing the previous 7 days. The daily anomaly data were then down sampled to weekly resolution corresponding to the self-report symptomatology questionnaires assessment frequency.

For each week detected as anomaly based on the activity data, two nearest consecutive surveys (core symptoms of depression [VQIDS-SR5] and anxiety [GAD-7]), corresponding to current week and the subsequent week were evaluated to ensure that anomaly in passive data was accompanied by symptom exacerbation. Based on discussions with clinicians, the criteria to assess the symptom exacerbation was defined a priori as the presence of moderate to severe symptomatology score in either of the surveys (VQIDS-SR5, GAD-7) or two mild symptomatology scores in both the surveys during the consecutive weeks.

The moderate to severe score for VQIDS-SR5 was set to ≥$6$ corresponding to QIDS-SR16 score of ≥ 11 (equivalent to MADRS score ≥ 19)^3,33,34^ and the mild score for VQIDS-SR5 was set to $5$ corresponding to QIDS-SR16 score of $9$ (equivalent to MADRS score of 16)^34,35^. The moderate to severe score range and the mild range are GAD-7 ≥ 10 and 10 > GAD-7 ≥ 5, respectively as reported in Spitzer et al ^36^.

### Continuous evaluation of the personalized relapse prediction framework

The continuous evaluation for the personalized relapse prediction framework for a single informative patient is shown in Supplement 3, Fig. S5. As mentioned before, each clinic visit was labelled as a relapse or non-relapse visit based on pre-specified clinical relapse criteria (Table 1). As clinic visits occurred at bimonthly intervals, the relapse versus non-relapse status at the current clinic visit was assumed to have been continuous since the time of the previous clinic visit. The number of available clinic visits (relapse and non-relapse) that were evaluated in both studies is shown in Supplement 3, Fig. S6. For each patient, there was an additional 2-month period of continuous activity data (Passive) with no relapses (between baseline and first clinic visit) which were used for the initial training of the N-of-1 LSTM EncDec-AD model. The different steps involved in the framework’s validation are discussed below:

1. The trained model was evaluated on the actigraphy features corresponding to the subsequent clinic visit (testing data) to obtain an anomaly score (Mahalanobis distance, ${a}^{(i)}$ = $\sqrt{{{(e}^{(i)}-\mu )}^{T}{\Sigma }^{-1}{(e}^{(i)}-\mu )}$ where. $e$ represents the reconstruction error in the testing data; the $\mu$ and $\Sigma$ represents the mean and covariance of the multivariate Normal distribution $\mathcal{N} (\mu ,\Sigma )$ of the actigraphy features in the training data).
2. The anomalous instances detected in the anomaly score are then investigated for an accompanied symptom exacerbation in self-reported assessments (Active) of core symptoms of depression (VQIDS-SR5) and anxiety (GAD-7).
3. If both passive and active were anomalous, the flag for impending relapse was raised, after which the framework stopped making further predictions until the patient was assessed for relapse in the subsequent clinic visit.
4. If either passive or passive combined with active were not anomalous, the framework continued to make further predictions until the patient was assessed for relapse in the subsequent clinic visit.
5. If the subsequent clinic visit was determined to be non-relapse, the LSTM EncDec-AD model was retrained with the additional 2 months period of continuous multivariate activity features, and the process repeats for every clinic visit for that patient.

A schematic representation of the deployment workflow of the proposed framework is shown in Fig. 2b**.** The proposed ‘Passive + Active’ modeling framework was compared with the ‘Active’ component, to understand the relative value of using passive actigraphy data, over and above relying solely on self-reported data**.** The ‘Active’ framework used the self-reports to assess the symptom exacerbation every week to raise a flag for the risk of imminent relapse.

## Results

### Clinical relevance of actigraphy features, risk factors of relapse and inter-individual variability

Among the actigraphy features, an increase in the ‘median of sample entropy during afternoon’ by one SD was associated with a 0.99% (2.1–0.12) decrease in the probability of relapse and 0.68 (1.12–0.23) unit decrease in depression severity (Supplement 3 Section V Fig. S7a–c). Furthermore, an increase of one SD in ‘variance of sample entropy during afternoon’ was associated with a 2.29% (4.89–0.31) decrease in the probability of relapse and 0.35 (0.67–0.03) unit decrease in depression severity (Supplement 3 Section V Fig. S7b–d).

The AME analysis of the self-reported symptom severity (VQIDS-SR5) (Supplement 3 Section V Fig. S8) showed similar trends with actigraphy features as observed with AME analysis for depression clinical outcome. A one SD increases in sample entropy during morning, afternoon, evening and DFA ${\alpha }_{1}$ was associated with 0.07-unit reduction in depression severity. Though there is a significant association between actigraphy features and depression outcome (relapse, symptom severity) at a population level, there was considerable inter-induvial variability as shown in Supplement 3 Fig. S9 necessitating the development of a personalized relapse prediction framework.

### Relapse prediction framework performance

The proposed personalized relapse prediction framework was evaluated using longitudinal data from 277 patients with MDD (OBSERVEMDD: 227, CBN-WELL: 50). Patients’ baseline characteristics and distribution of all bimonthly clinic visits that were evaluated for relapse and non-relapse patients are summarized in Table 2 and Supplement 3 Fig. S6, respectively. There was no statistical difference between baseline and demographic characteristics of relapsed and non-relapsed patients.

**Table 2:** Baseline demographic characteristics.

Characteristic	OBSERVEMDD			CBN-WELL		
	Relapse	Non-Relapse	Statistical Test	Relapse	Non-Relapse	Statistical Test
	(N = 66)	(N = 161)	*P* value	(N = 14)	(N = 36)	*P* value
Age (years), mean (SD)	46 (11.47)	43 (13.16)	0.13	44.14 (14.27)	39.94 (12.49)	0.33
Age category (years), n (%)						
Age 18–44	28 (42)	82 (50.93)	0.31	7 (50)	23 (63.9)	0.56
Age ≥ 45	38 (58)	79 (49.06)		7 (50)	13 (36.1)	
Sex, n (%)						
Men	18 (28)	48 (29.81)	0.82	5 (35.7)	22 (38.9)	0.19
Women	48 (72)	113 (70.19)		9 (64.3)	14 (61.1)	
Race, n (%)						
White	50 (75.7)	129 (80.12)	0.79	NA	NA	NA
Black or African American	11 (16.6)	23 (14.29)		NA	NA	
Asian	1 (1.5)	5 (3.1)		NA	NA	
Native Hawaiian or other Pacific Islander	1 (1.5)	1 (0.62)		NA	NA	
Unknown	2 (3)	2 (1.24)		NA	NA	
Other	1 (1.5)	1 (0.62)		NA	NA	
Ethnicity, n (%)						
Hispanic or Latino	4 (6)	7 (4.34)	0.07	0 (0)	3 (8.3)	NA
Not Hispanic or Latino	60 (90.9)	154 (95.65)		14 (100)	33 (91.67)	
Not reported	2 (3)	0 (0)		0 (0)	0 (0)	
Weight (kg), mean (SD)	87.15 (26.89)	87.55 (22.28)	0.52	90.93 (23.29)	80.58 (20.36)	0.14
BMI (kg/m^2^), mean (SD)	30.51 (8.65)	30.97 (7.92)	0.56	32.11 (7.29)	27.41 (7.09)	0.13

BMI, body mass index; SD, standard deviation.

Although some patients experienced multiple relapses during the study period, only data up until the first relapse have been analyzed and no training or predictions were performed after the first relapse. The performance of the relapse prediction framework across two studies are shown in Table 3.

**Table 3:** Relapse prediction framework performance on unseen test data with provider burden, and patient burden.

Dataset	Prediction performance							
	Framework	SEN (%)	SPEC (%)	BAC (%)	PPV (%)	NPV (%)	FPR (%)	FAR (%, per patient-year)
OBSERVEMDD	Active	76	68	72	9	98.6	32	31, 2.35
	Passive + Active	66	81	73.5	12.5	98.3	19	18.5, 1.8
CBN-WELL	Active	90	60	75	13.2	98.9	40	37.8, 3
	Passive + Active	70	72	71	14.6	97.2	28	26.2, 2.3

Dataset	Provider burden							
	Framework		Total number of patient days of observation (N)		Total number of preemptive visits (N)		Provider burden (per patient-year)	
OBSERVEMDD	Active		59,606		422		2.58	
	Passive + Active		47,378		265		2.04	
CBN-WELL	Active		7196		68		3.45	
	Passive + Active		6487		48		2.7	

Dataset	Patient burden							
	Framework				Total number of self-report assessments required (N, %)			
OBSERVEMDD	Active				8515, 100			
	Passive + Active				3240, 38.1			
CBN-WELL	Active				1028, 100			
	Passive + Active				484, 47			

BAC, balanced accuracy; FAR, false alarm rate; NPV, negative predictive value; PPV, positive predictive value; SEN, sensitivity; SPEC, specificity.

Provider burden was estimated based on the total number of preemptive visits (True Positive + False Positives) to the total number of patient days of observation; for instance, 2.58 relapse alarm/patient/year is obtained by 422/59,606. Patient burden was estimated based on the total number of self-report assessments required to be completed by the patient.

Since the “Active” framework was based on self-reports collected on a weekly basis, we defined it as the maximal patient burden (100%) and estimated the relative patient burden in “Active + Passive” framework as a fraction of the total self-reports used in that framework.

### Relapse prediction framework performance in OBSERVEMDD

The proposed personalized framework (‘Passive + Active’) achieved a balanced accuracy of 73.5% (sensitivity, 66%; specificity, 81%) with an overall false alarm rate (FAR) of 18.5% (1.8 false alarm/patient/year) and false positive rate (FPR) of 19%. The median time of detection (TOD) was 21 days (interquartile range: 7–28) in advance of next clinic visit. The overall observed prevalence rate of relapse was 29.1% on the population level (66/227 patients) and 3.99% on the clinic visit level (50/1254 clinic visits). For this prevalence, the model achieved a positive predictive value (PPV) of 12.5%, negative predictive value (NPV) of 98.3% (Table 3). In comparison, the ‘Active’ framework achieved a balanced accuracy of 72% (sensitivity, 76%; specificity, 68%), PPV of 9%, NPV of 98.6% and FAR of 31% (2.35 false alarm/patient/year), FPR of 32% leading to an increase of 12.5% in FAR and an increase of 13% in FPR compared to ‘Passive + Active’.

The provider and patient burden for ‘Passive + Active’ and ‘Active’ frameworks were also estimated to better understand the relative value of using passive data and its impact on deployment (Table 3). The patient burden here refers the time and effort required to fill out self-reported questionnaires and provider burden refers the time and effort by the provider to follow-up on alarm raised by the prediction framework.

The provider burden of ‘Active’ framework and ‘Passive + Active’ framework was 2.58 and 2.04 relapse alarm/patient/year, respectively (Table 3). The ‘Passive + Active’ framework yields a reduction in patient burden by 61.9% over the ‘Active’ only framework (Table 3).

### Relapse prediction framework performance in CBN-WELL

The proposed personalized framework (‘Passive + Active’) achieved a balanced accuracy of 71% (sensitivity, 70%; specificity, 72%) with an overall FAR of 26.2% (2.3 false alarm/patient/year) and FPR of 28%. The median TOD was 14 days (interquartile range: 14–26.25) in advance of next clinic visit. The overall observed prevalence rate of relapse and recurrence in CBN-WELL was 28% on the population level (14/50 patients) and 6.41% on the clinic visit level (10/156 clinic visits). For this prevalence, the model achieved a PPV of 14.6%, NPV of 97.2% (Table 3). In comparison, the ‘Active’ framework achieved a balanced accuracy of 75% (sensitivity, 90%; specificity, 60%), PPV of 13.2%, NPV of 98.9% and FAR of 37.8% (3 false alarm/patient/year), FPR of 40% leading to an increase of 11.6% in FAR and an increase of 12% in FPR compared to ‘Passive + Active’.

The provider burden of ‘Active’ framework and ‘Passive + Active’ framework was 3.45 and 2.7 relapse alarm/patient/year, respectively (Table 3). The “Passive + Active” framework yields a reduction in patient burden by 53% over the “Active” only framework (Table 3).

In addition, the performances of the framework were assessed across sex and age ranges (18–44 years and 45–64 years) and the results were generalizable across comparisons in two studies. The median balanced accuracy of the framework was 71% (interquartile range: 70%—75%) with median FAR of 22.2% (interquartile range: 20.3%–25.2%) leading to a median provider burden of 2.35 alarm/patient/year (interquartile range: 2.15–2.65) with a reduced patient burden (median: 50% [interquartile range: 44%–53.2%]) requiring only 50% of the self-reported assessments compared to completing surveys on a standardized periodic basis (every week in this case). The detailed results are provided in Supplement 3 (Section VI, Tables S3–S6).

## Discussion

In this study, the predictive ability of a personalized (N-of-1) relapse prediction framework was demonstrated using a combination of passive (actigraphy) and active (self-reported symptoms) measures in 277 MDD patients from two independent, prospectively followed cohorts from the OBSERVEMDD and CBN-WELL studies.

The proposed framework predicted the majority of the relapses that occurred in the study populations and provided 2–3 weeks lead-in time for possible relapse preemption with minimal patient and provider burden. The N-of-1 relapse prediction framework should be inherently generalizable, since patients own data are being used to make predictions. In fact, this framework produced comparable performance in the two independent cohorts with activity data collected using two different wearable devices. To the best of our knowledge, this is the first study to report implementation of an N-of-1 relapse prediction framework using a unique combination of active and passive data to continuously predict the relapse in MDD population. This is also the first study to report replicable results in two independent cohorts to continuously predict relapse in MDD population using longitudinal data collection ($\ge$ 1 year). This approach could afford a potentially significant clinical opportunity towards predictive, proactive, and personalized patient care utilizing digital correlates of symptom evolution.

Predicting relatively rare events, such as relapse in a heterogeneous disease like MDD, faces the dual challenge of the paucity of available training data for relapse episodes and the limitations on generalizability across individual participants. Furthermore, if the predictive model is based on self-reported data that is frequently collected over a long period and has a high FAR, it could lead to higher patient and provider burden during clinical care.

Our modeling approach addresses the challenge of generalizability by developing a personalized (N-of-1) relapse prediction framework using the anomalies detected in passive digital biomarkers, such as actigraphy patterns in which data from each individual is used to train the LSTM EncDec-AD. Since the LSTM EncDec-AD trains on prior data of an individual, our framework does not rely on the same features to generalize across all individuals rather it looks at multivariate anomaly across the features on an individual basis. We see that as a particular strength of this approach. Through this framework, the generalizability across individuals and different populations has been achieved by learning multivariate anomaly patterns pertaining to significant features for each individual, which need not be identical, but they do share commonalities. One of the prerequisites for building such personalized models is longitudinal passive data collection and regular clinic assessments. The two studies that we have utilized are the first of its kind in MDD to acquire passive data across 277 patients with $\ge$ 1 year of follow-up with regular clinic assessments which enabled the development of personalized (N-of-1) relapse prediction models.

These anomalies detected from actigraphy data using the personalized (N-of-1) models are then assessed for accompanied self-reported symptom exacerbation, providing additional context to the actigraphy triggered anomalies to determine the risk of an imminent relapse. The number of self-reported symptoms assessed corresponds to a total of 12 questions (VQIDS-SR5 and GAD-7 items), which were only assessed during anomalous instances identified in actigraphy data, thus reducing the patient burden (compared to completing surveys on a standardized periodic basis).

In addition, the features derived from actigraphy data quantify the fractal motor activity regulation^11^, circadian regulation^11^, and entropic signatures^19^ reflecting the complexity of physiological control. A degradation in these metrics corresponds to reduced complexity in the system making it less adaptable to perturbations and more vulnerable to catastrophic events such as relapse^24^. These signatures were also observed in our work, by the negative associations between actigraphy features (sample entropy during morning, afternoon, and evening time periods and DFA ${\alpha }_{1}$) and increasing levels of depression severity. This relationship indicated that with increasing levels of depression severity, the activity patterns become more monotonous (less variable), as reflected by decreased fractal regulation and entropy features.

Furthermore, these features are less affected by uncontrolled daily schedules and environmental conditions compared to actigraphy-derived measures of sleep. Also, actigraphy features used in our framework were derived from activity counts rather than proprietary data streams to keep open the possibility of a device-agnostic, scalable deployment as demonstrated in our evaluation with two different wearable devices with access to activity counts.

Patients who are deemed to be at high risk for relapse by their clinicians after being treated for MDD could be enrolled in a relapse risk monitoring program using the personalized prediction framework described above. In the proposed framework, the actigraphy data were collected continuously, and the relapse status was determined by the clinician during the bimonthly visit. If the visit was determined to be a non-relapse, then all actigraphy data until the current clinic visit since the enrollment visit were used to train the EncDec-AD model. The trained model was used to detect anomalous instances in the subsequent clinic visit actigraphy data. The detected anomalous instances were then assessed for accompanied self-reported symptomatology exacerbations to raise flag for impending relapse. This process continued with every non-relapse clinical visit and the EncDec-AD got retrained and the updated model detected anomaly in the subsequent clinic visit activity data, which were then assessed for accompanied self-reported symptomatology exacerbations. For example, once a personalized framework predicted relapse risk, the framework would stop making further predictions and could lead to a preemptive appointment or a phone call from the clinician’s office to check on the patient’s symptoms when deployed in clinic care setting. Based on clinician’s judgment, an early intervention could be administered if deemed needed, which could eventually prevent an impending relapse and lead to better patient outcomes. Once the patient was stabilized, the framework could continue to train and predict future risk of relapses.

The proposed framework achieved a high NPV indicating a negative result is highly predictive of non-relapse. It also achieved a balanced accuracy and actionable time frame (2–3 weeks) better than the recent prior research^2^ on relapse prediction, leading to a more reliable continuous relapse prediction framework in MDD.

The PPV must be interpreted in the context of low prevalence of relapse events (28% to 29.1% on the population level and 3.99% to 6.41% on the clinic visit level) and the cost of an undetected relapse being relatively high in terms of patient morbidity, psychosocial burden when compared the cost of intervention (e.g., bringing the patient in for an earlier clinic visit). In addition, the provider burden of the proposed predictive framework (≤ 2.7 relapse alarm/patient/year across both studies) is relatively low which coupled with the high NPV, provides promise for this predictive framework’s utility as a tool for clinical resource management.

As observed, the performance of the personalized (N-of-1) relapse prediction framework is comparable in both studies except for the decrease in specificity. The paucity of longitudinal training data across patients quantified with > 2 clinic visits (N_>2_) in CBN-WELL (N_>2_ = 26) compared to OBSERVEMDD (N_>2_ = 169) may have contributed to decreased specificity, as the specificity of the N-of-1 approach improves with continuous training on more longitudinal data.

This study has limitations that may confound some of its findings. The proposed personalized framework uses self-reported symptom assessments for final determination of relapse risk. Though the self-rated symptom severity scales have been clinically validated, there could be scenarios where the patient (due to symptom burden or impaired insight) may have difficulties in completing the self-report assessments, which could undermine the performance of the personalized relapse prediction framework. One possibility to mitigate this would be to replace the self-reported assessments with other objective active digital biomarkers capturing different MDD symptom domains to further assess the relapse risk status. Additionally, the anomaly detection algorithm currently requires actigraphy data obtained using a wearable actigraphy device, which may limit participation and adherence. However, the proposed relapse prediction framework only requires a minimum of 2 consecutive days of continuous activity data collection, potentially easing its uptake.

In conclusion, we have presented a generalizable framework to predict and thus preempt relapses using an efficient combination of passive and active patient generated data. The anomaly detection component that utilizes passive data could be customized for predicting events in other neuropsychiatric disorders (e.g., bipolar disorder or schizophrenia) by including other relevant passive sensing modalities. This could be combined with disease-specific self-reported assessments or other relevant active data streams to predict relapse in those disorders.

### Supplementary Information

Supplementary Information 1.

Supplementary Information 2.

Supplementary Information 3.

## Data Availability

The data sharing policy of Janssen Pharmaceutical Companies of Johnson & Johnson is available at https://www.janssen.com/clinical-trials/transparency. Although these data are not currently publicly available for sharing based on its proprietary nature. The request for sharing can be sent to the Corresponding Author (Srinivasan Vairavan, PhD; Email: svairava@its.jnj.com) and will be evaluated on an individual basis.

## Code availability

Based on the proprietary nature of modifications made to the code to accommodate the passive data, it may not be made available in all cases. Requests for access to source code would require evaluation on an individual basis.
