# Supplementary material for: Personalized relapse prediction in patients with major depressive disorder using digital biomarkers
Source: Sci Rep. 2023 Oct 30;13:18596. doi: 10.1038/s41598-023-44592-8 (PMC10616277; doi:10.1038/s41598-023-44592-8)
Supplement: Supplementary file 1 — Supplementary Information 1. [file 41598_2023_44592_MOESM1_ESM.pdf]

Janssen Research & Development *

Clinical Protocol

A Prospective, Longitudinal, Observational Study to Evaluate Potential Predictors of Relapse in Subjects With Major Depressive Disorder Who Have Responded to Antidepressant Treatment

Protocol OBSERVEMDD0001

*Janssen Research & Development is a global organization that operates through different legal entities in various countries. Therefore, the legal entity acting as the sponsor for Janssen Research & Development studies may vary, such as, but not limited to Janssen Biotech, Inc.; Janssen Products, LP; Janssen Biologics, BV; Janssen-Cilag International NV; Janssen, Inc; Janssen Infectious Diseases BVBA; Janssen R&D Ireland; or Janssen Research & Development, LLC. The term "sponsor" is used throughout the protocol to represent these various legal entities; the sponsor is identified on the Contact Information page that accompanies the protocol.

Status: Approved
Date: 4 September 2014
Prepared by: Janssen Research & Development, LLC
EDMS number: EDMS-ERI-82446171, 1.0

GCP Compliance: This study will be conducted in compliance with Good Clinical Practice, and applicable regulatory requirements.

Confidentiality Statement
The information in this document contains trade secrets and commercial information that are privileged or confidential and may not be disclosed unless such disclosure is required by applicable law or regulations. In any event, persons to whom the information is disclosed must be informed that the information is privileged or confidential and may not be further disclosed by them. These restrictions on disclosure will apply equally to all future information supplied to you that is indicated as privileged or confidential.

TABLE OF CONTENTS

TABLE OF CONTENTS 2

LIST OF ATTACHMENTS 4

LIST OF IN-TEXT TABLES AND FIGURES 4

SYNOPSIS 5

TIME AND EVENTS SCHEDULE 13

TIME AND EVENTS SCHEDULE:SUBJECT-COMPLETED ASSESSMENTS 16

ABBREVIATIONS 20

1. INTRODUCTION 21
1.1. Background 21
1.2. Overall Rationale for the Study 21

2. OBJECTIVES 22

3. STUDY DESIGN AND RATIONALE 23
3.1. Overview of Study Design 23
3.2. Study Design Rationale 24

4. SUBJECT POPULATION 33
4.1. Inclusion Criteria 33
4.2. Exclusion Criteria 33

5. TREATMENT ALLOCATION AND BLINDING 34

6. DOSAGE AND ADMINISTRATION 34

7. ANTIDEPRESSANT TREATMENT COMPLIANCE 34

8. PRESTUDY AND CONCOMITANT THERAPY 35

9. STUDY EVALUATIONS 35
9.1. Study Procedures 35
9.1.1. Overview 35
9.1.2. Screening Phase 37
9.1.2.1. Early Trauma Inventory Self-Report – Short Form 37
9.1.2.2. Cumulative Illness Rating Scale 38
9.1.2.3. MINI International Neuropsychiatric Interview 38
9.1.2.4. Massachusetts General Hospital Antidepressant Treatment Response Questionnaire 38
9.1.3. Observational Phase 38
9.2. Clinical Assessments 39
9.2.1. Evaluations 39
9.2.1.1. Montgomery Asberg Depression Rating Scale 39
9.2.1.2. Clinical Global Impression - Severity 40
9.2.1.3. Snaith Hamilton Pleasure Scale 40
9.2.1.4. Quick Inventory of Depressive Symptomatology-Self Report-16-Item 40
9.2.1.5. Seven-Item Generalized Anxiety Disorder Scale 41
9.2.1.6. Perceived Stress Scale 41
9.2.1.7. Medical Outcomes Study Sleep Scale, Revised, 12-Items 41
9.2.1.8. EuroQol Group; 5 Dimension; 5 Level 41
9.2.1.9. World Health Organization Disability Assessment Schedule 2.0 42
9.2.1.10. Recent Life Change Stress Test 42

9.2.1.11. The Pain Frequency, Intensity, and Burden Scale 42
9.2.1.12. Healthcare Resource Use Questionnaire 43
9.2.1.13. Depression Implicit Association Test 43
9.2.1.14. Assessment of Speech and Voice 43
9.2.1.15. Actigraphy 44
9.2.1.16. First Symptom of Relapse – Self-Assessment 44
9.2.2. Endpoints 44
9.3. Fluid Biomarkers 45
9.4. Genetic and Epigenetic (DNA) and Transcriptomic (RNA) Evaluations 45
9.5. Healthcare Resource Utilization 46
9.6. Safety Evaluations 46
9.7. Sample Collection and Handling 47

10. SUBJECT COMPLETION/WITHDRAWAL 48
10.1. Completion 48
10.2. Withdrawal From the Study 48

11. STATISTICAL METHODS 49
11.1. Subject Information 49
11.2. Sample Size Determination 49
11.3. Analysis 50
11.4. Analyses of Fluid Biomarkers and Genetic, Epigenetic, and Transcriptomics Data 51
11.5. Healthcare Resource Utilization Analyses 51
11.6. Safety Analyses 51
11.7. Adjudication Committee 52

12. MANAGEMENT AND REPORTING OF ADVERSE EVENTS/ADVERSE REACTIONS 52
12.1. Definitions 52
12.1.1. Adverse Event Definitions and Classifications 52
12.1.2. Attribution and Severity Definitions 54
12.2. Special Situations 55
12.3. Pregnancy 56
12.4. Pharmacovigilance and Reporting Procedures 56
12.4.1. Reporting Procedures for Adverse Events and Pregnancies or Pregnancies in Partners for Janssen Products 56
12.4.2. Reporting Procedures for Serious Adverse Events and Pregnancies or Pregnancies in Partners for Non-Janssen Medicinal Products 57
12.5. Product Quality Complaint Handling 57

13. STUDY-SPECIFIC MATERIALS 58

14. ETHICAL ASPECTS 58
14.1. Study-Specific Design Considerations 58
14.2. Regulatory Ethics Compliance 59
14.2.1. Investigator Responsibilities 59
14.2.2. Independent Ethics Committee or Institutional Review Board 59
14.2.3. Informed Consent 61
14.2.4. Privacy of Personal Data 61
14.2.5. Long-Term Retention of Samples for Additional Future Research 62
14.2.6. Country Selection 62

15. ADMINISTRATIVE REQUIREMENTS 62
15.1. Protocol Amendments 62
15.2. Regulatory Documentation 63
15.2.1. Regulatory Approval/Notification 63
15.2.2. Required Prestudy Documentation 63
15.3. Subject Identification, Enrollment, and Screening Logs 64
15.4. Source Documentation 64
15.5. Case Report Form Completion 65

15.6. Data Quality Assurance/Quality Control 66
15.7. Record Retention 66
15.8. Monitoring 67
15.9. Study Completion/Termination 67
15.9.1. Study Completion 67
15.9.2. Study Termination 67
15.10. On-Site Audits 68
15.11. Use of Information and Publication 68

REFERENCES 70

INVESTIGATOR AGREEMENT 108

LAST PAGE 108

LIST OF ATTACHMENTS

Attachment 1: Patient Adherence to Antidepressant Medication Questionnaire 74
Attachment 2: Early Trauma Inventory Self Report-Short Form 75
Attachment 3: Cumulative Illness Rating Scale 76
Attachment 4: Massachusetts General Hospital Antidepressant Treatment Response Questionnaire 77
Attachment 5: Montgomery Asberg Depression Rating Scale 79
Attachment 6: Clinical Global Impression – Severity 84
Attachment 7: Snaith Hamilton Pleasure Scale 85
Attachment 8: Quick Inventory of Depressive Symptomatology-Self Report-16-Item 87
Attachment 9: Generalized Anxiety Disorder 7-Item Scale 89
Attachment 10: Perceived Stress Scale 90
Attachment 11: Medical Outcomes Study Sleep Scale, Revised, 12 Items 91
Attachment 12: EuroQol Group: 5 Dimension, 5 Level 93
Attachment 13: World Health Organization Disability Assessment Schedule 96
Attachment 14: Recent Life Change Stress Test (Baseline) 98
Attachment 15: Recent Life Change Stress Test (Since Last Visit) 100
Attachment 16: Pain, Frequency, Intensity, and Burden Scale 102
Attachment 17: First Symptom of Relapse - Self-Assessment 103
Attachment 18: Concise Health Risk Tracking Scale (Baseline) 104
Attachment 19: Concise Health Risk Tracking Scale (Since Last Visit) 106

LIST OF IN-TEXT TABLES AND FIGURES

TABLES

Table 1: STAR*D Study: Remission Status at Follow-Up Entry and Relapse Rates for Participants Entering Follow-Up From Each Treatment Step 27
Table 2. Clinical Assessments, by Parameter and Domain 29
Table 3. Volume of Blood to Be Collected From Each Subject 37
Table 4. Precision Level and Confidence Intervals for Sample Sizes of 300 to 500 Subjects and Relapse Rates of 30% to 50% 50

FIGURES

Figure 1. Schematic Overview of Study OBSERVEMDD0001 24
Figure 2. Width of the 95% Confidence Interval for Different Expected Proportions and Total N 50

SYNOPSIS

A Prospective, Longitudinal, Observational Study to Evaluate Potential Predictors of Relapse in Subjects With Major Depressive Disorder Who Have Responded to Antidepressant Treatment

Major depressive disorder (MDD) is a chronic disorder with recurrent episodes. Relapse (within the same episode) or recurrence (start of a new episode) typically occurs gradually over weeks. There is limited information known about precursors to, or predictors of, relapse or recurrence. The course of improvement from an acute depressive episode to remission is better known from short-term studies. However, imminent precursors to relapse are not as well known, since patients often seek medical attention only after relapse has occurred.

There is very limited knowledge with regard to changes in MDD patients during continuation/ maintenance treatment that may be indicative of early stages of a relapse prodrome or impending relapse. Few studies have examined markers that may change or appear during continuation/maintenance treatment that may be more immediate precursors to relapse. If markers of a likely relapse within a period of weeks can be identified, then clinicians might conduct further clinical evaluation and adjust treatment as appropriate for patients who exhibit such markers of impending relapse. In this study, analyses will attempt to identify changes/markers of relapse within several weeks to 1 month; various time frames will be explored in predictive modeling, and final models will be considered based on performance. Identification of markers of impending MDD relapse may enable strategies to intercept disease worsening, eg, (re)introduction of rapidly acting antidepressants or other appropriate treatment interventions.

OBJECTIVES

Primary Objective

The objective of this study is to identify if there are self-reported or objective measures related to mood parameters that can predict near-term relapse (within 1 month or at another identified time point before meeting the criteria for relapse) or early symptomatic changes indicative of relapse prodrome in MDD. Domains to be assessed include mood and anxiety symptoms, stress, sleep, motor activity, pain, health, functioning, and quality of life, implicit cognitive processes, compliance with the oral antidepressant treatment regimen, and speech and voice characteristics.

This is an exploratory prospective study to derive a predictive model(s) of relapse in MDD. Rather than testing of specific hypotheses about particular variables, a range of variables will be measured and evaluated as potential predictors of relapse, including depressive and anxiety symptoms, health, functioning, and quality of life, sleep, behavior/activity, and speech, observed either via remote monitoring or obtained at regular study visits.

OVERVIEW OF STUDY DESIGN

This is a prospective, multicenter, longitudinal, single-cohort, observational study in subjects with MDD who have responded to, and are continuing to respond to and receive, an oral antidepressant treatment regimen. Approximately 330 subjects will be enrolled in order to result in 150 subjects who relapse. The study will consist of 2 phases: a screening phase of up to 2 weeks, and an observational phase of variable duration, with study visits every 8 weeks until relapse has been observed in 150 subjects. The total study duration for each subject will therefore be variable. Subjects who relapse will continue in the observational phase, with study visits every 8 weeks, until relapse has been observed in 150 subjects (ie, the intent is to follow all relapsed and non-relapsed subjects in the observational phase). Relapse will only be counted once for any given subject. The end of the study will be defined as the date of the last visit of the last subject participating in the study, once relapse has been observed in 150 subjects.

Once relapse has been observed in 150 subjects, all subjects will conclude study participation with an end-of-study visit conducted at the next scheduled study visit (ie, 8-week interval), and will then be referred for further follow-up with their treating physician.

To be eligible to enter the study, a subject must have met Diagnostic and Statistical Manual of Mental Disorders (5th edition) (DSM-5) diagnostic criteria for nonpsychotic, recurrent MDD within the past year (ie, the start of the current major depressive episode (MDE) must be ≤1 year before screening), but must not currently meet criteria for MDD; have a Montgomery Asberg Depression Rating Scale (MADRS) total score ≤14; have evidence of recent response (within the past 3 months) to an oral antidepressant treatment regimen (taken at an optimal dosage and for an adequate duration); and be currently taking and responding to an oral antidepressant treatment regimen. A change in the oral antidepressant treatment regimen since the time of achieving response in the current episode will be allowed. All antidepressant medication taken for the current MDE will be captured on the Massachusetts General Hospital Antidepressant Treatment Response Questionnaire (MGH-ATRQ), with corroboration from medical and pharmacy records, if available.

After consenting to participate in the study, subjects will undergo a brief diagnostic interview (MINI International Neuropsychiatric Interview; MINI - DSM-5) to confirm that the diagnosis of recurrent MDD was present prior to their antidepressant response, and to determine if there are other psychiatric conditions present.

At baseline, subjects will complete assessments of mood, as well as other assessments as outlined in the Time and Events Schedule. All participants will receive a study-specific smartphone (LogPad®), which they will use to complete assessments at and between study visits. Motor activity and sleep parameters will be captured using actigraphy via a wrist-worn device throughout the study period. Subjects will receive reminders to complete questionnaires via the study-provided smartphone. Clinicians will also remind subjects at study visits of the importance of completing self-report measures between visits. Subjects will continue to participate in treatment as directed by their clinician, and will return for visits to the clinic every 8 weeks.

An Adjudication Committee will be commissioned for this study, to adjudicate any cases of relapse where it is unclear if relapse occurred or where further discussion is needed.

SUBJECT POPULATION

The study will include men and women between 18 and 64 years of age, inclusive, who have met DSM-5 criteria for diagnosis of nonpsychotic, recurrent MDD within the past year (ie, the start of the current MDE must be ≤1 year before screening) who have demonstrated an adequate response within the past 3 months, and are currently maintaining this response, to an oral antidepressant treatment regimen of adequate dosage and duration. At screening, subjects are required to have a MADRS total score ≤14. Subjects with a history of drug or alcohol use, with a severity of at least moderate or severe, according to DSM-5 criteria, within 6 months before screening will be excluded. Subjects will also be excluded with any of the following DSM-5 psychiatric diagnoses: MDD with psychotic features (lifetime), bipolar disorder (including lifetime diagnosis), schizophrenia, or schizoaffective disorder.

DOSAGE AND ADMINISTRATION

Study subjects will continue to receive their oral antidepressant treatment regimen per their treating physician/clinician during the study. Adjustment to the oral antidepressant treatment regimen (if applicable) will be allowed as clinically indicated, including if a subject relapses, and will be documented in the case report form. The investigator will be required to document why a change was made to the oral antidepressant treatment regimen.

CLINICAL ASSESSMENTS

The measures selected for this study represent symptom and behavior domains identified as potential predictors of relapse. Given the exploratory nature of this study, a comprehensive set of assessments is included, with the objective of developing the most robust predictive models of impending relapse.

The primary measure to evaluate depressive symptoms, including whether relapse has occurred, will be the MADRS total score. The MADRS is a 10-item clinician-rated scale, designed to measure depression severity and detects changes due to antidepressant treatment. The MADRS evaluates apparent sadness, reported sadness, inner tension, sleep, appetite, concentration, lassitude, interest level, pessimistic thoughts, and suicidal thoughts.

Other measures will include:

- The Clinical Global Impression – Severity (CGI-S) is a physician-rated scale that is designed to rate the severity of the subject's illness at the time of assessment, relative to the clinician's past experience with subjects who have the same diagnosis and improvement with treatment.
- The Snaith-Hamilton Pleasure Scale (SHAPS) is a brief 14-item patient-reported assessment scale for estimation of the degree to which a person is able to experience pleasure or the anticipation of a pleasurable experience. The SHAPS covers 4 domains of hedonic experience: interest/pastimes, social interaction, sensory experience, and food/drink.
- The Quick Inventory of Depressive Symptomatology-Self Report-16-Item (QIDS-SR_{16}) is a patient-reported measure designed to assess the severity of depressive symptoms. The QIDS-SR_{16} assesses all the criterion symptom domains designated by the DSM-5 to diagnose a major depressive episode. This assessment can be used to screen for depression, although it has been used predominantly as a measure of symptom severity.
- The 7-item Generalized Anxiety Disorder Scale (GAD-7) is a validated, brief 7-item self-report assessment of anxiety.
- The Perceived Stress Scale (PSS) is a 10-item, self-reported, unidimensional instrument developed to measure perceived stress in response to situations in a person's life. The PSS is a widely used scale for measuring the perception of stress. It is a measure of the degree to which situations in one's life are appraised as stressful. Items are designed to tap how unpredictable, uncontrollable, and overloaded respondents find their lives. The scale also includes a number of direct queries about current levels of experienced stress. Moreover, the questions are of a general nature, and hence are relatively free of content specific to any subpopulation group.
- The Medical Outcomes Study Sleep Scale, Revised (MOS Sleep-R) is a subject-completed scale containing 12 items that addresses various dimensions of sleep. The instrument yields 6 subscales: sleep disturbance, snoring, shortness of breath or headache, sleep adequacy, sleep somnolence, and sleep quantity.
- The EuroQol Group; 5-Dimension; 5-Level (EQ-5D-5L) is a standardized 2-part instrument for use as a measure of health outcome, primarily designed for self-completion by respondents. It essentially consists of the EQ-5D descriptive system and the EQ visual analogue scale (EQVAS). The EQ-5D-5L descriptive system comprises the following 5 dimensions: mobility, self-care, usual activities, pain/discomfort, and anxiety/depression.
- The World Health Organization Disability Assessment Schedule 2.0 (WHODAS 2.0) is a generic instrument developed by the WHO to provide a standardized method for measuring health and disability across cultures. The WHO-DAS 2.0 is a 12-item self-administered scale measuring the amount of difficulty a subject has completing specific activities. The scale assesses the following 6 domains: cognition, mobility, self-care, getting along (interacting with other people), life activities, and participation.

- The Recent Life Changes Stress Test (RLCST) is a subject-completed scale that measures the number of life change events, such as marriage or death of a family member, the subject has experienced or expects to experience in the near future. Each item has been constructed to contain life events whose advent is either indicative of or requires a significant change in the ongoing life pattern of the individual.
- The Pain Frequency, Intensity, and Burden Scale (P-FIBS) is a 4-item brief self-reported measure developed to assess pain in research settings or as a screening tool in clinical settings. Burden of pain is assessed with 2 items, 1 assessing the extent to which pain interferes with daily life and 1 assessing the use of medications or other treatment to manage pain.
- The Healthcare Resource Use Questionnaire (HRUQ) is a clinician-administered assessment, developed by Janssen Research & Development, of the utilization of health care services, including hospitalizations, emergency room visits, day hospital attendance, outpatient visits, and work productivity.
- The First Symptom of Relapse – Self-Assessment (FSR-SA) is a single-item assessment that will be completed by all subjects who relapse, with the objective of providing additional information from the subject’s perspective about potential predictors of relapse.
- The Harvard/Project Implicit Mental Health (PIMH) depression-specific Implicit Association Test (IAT) will be used in this study. Association tests are designed to assess automatic cognitive processes; subjects are asked to categorize a concept (eg, “me” vs “not me”) with an attribute (eg, “logical”) by responding to stimulus words (ie, stimulus words including synonyms as well as antonyms of the attribute being tested). Reaction time is measured; faster responding is interpreted to represent stronger automatic associations between concept and attribute.
- Speech and voice characteristics will be assessed using speech diary recordings via a study-specific smartphone (LogPad®). Subjects will use a software application on the smartphone to record speech at 2-week intervals. The application will prompt each subject to describe:
  - His or her physical condition during the past 2 weeks
  - His or her mental condition during the past 2 weeks
  - How his or her physical and mental feelings have affected his or her ability to function during the past 2 weeks
  - The event during the past 2 weeks that made him or her the happiest
- Activity and sleep parameters will be measured using an Actiwatch® Spectrum wrist actigraphy device. Actigraphy measures will include:
  - Sleep duration
    - Total sleep time per night (hours)
  - Sleep pattern
    - Time of sleep onset (time of day)
    - Time of sleep end (time of day)
  - Sleep quality
    - Mean activity during rest period (counts/minute)
    - Sleep fragmentation (%)
    - Sleep efficiency (%)
    - Actigraphic estimate of wake after sleep onset (minutes)

- Daytime activity
    - Mean daytime activity (counts/minute)
    - Peak daytime activity (counts/minute)
- Antidepressant medication adherence will be assessed in 2 ways:
    - The Patient Adherence to Antidepressant Medication Questionnaire (PAQ) is a brief, 2-item self-report measure to assess how often the subject has taken, and whether he/she has made any changes to, his/her antidepressant treatment regimen in the last week.
    - The investigator will document the subject's antidepressant medication prescription history (eg, original date filled, quantity, refill frequency).

Primary Endpoints

The objective of this exploratory, prospective cohort trial is to identify if there are self-reported or objective measures that can predict near-term relapse (approximately 1 month, or at another identified time point, prior to meeting the criteria for clinical relapse). Subjects will be followed in the study until relapse has been observed in 150 subjects. Subjects may participate in the study for a variable duration until the target number of relapses has occurred. Once relapse has been observed in 150 subjects, all subjects will conclude study participation with an end-of-study visit conducted at the next scheduled study visit (ie, 8-week interval), and will then be referred for further follow-up with their treating physician. Relapse will be analyzed as the dependent variable in statistical analyses (ie, modeling re: predictors of relapse).

Subjects who relapse will continue to be followed in the study, ie, the intent is to continue to follow all relapsed and non-relapsed subjects. Relapse will only be counted once for any given subject.

Criteria

Relapse will be defined as any of the following:

- MADRS total score ≥22 for at least 2 consecutive weeks. If this criterion is met at a study visit (scheduled or unscheduled), an additional visit (ie, the Relapse Verification visit) will be scheduled within 1 to 2 weeks to verify the relapse.
- Hospitalization for worsening of depression
- Suicidal ideation with intent, or suicidal behavior
- Other (investigator will be asked to describe)

FLUID BIOMARKER EVALUATIONS

Blood samples will be collected to study the potential association of biomarkers with relapse in MDD, using the Human Discovery MAP™ (Multi-Analyte Panel), a panel of approximately 250 proteins involved in key biologically relevant pathways associated with major depression, including neurotrophic and inflammatory factors. In addition, analysis of specific biomarker analytes may include (but not limited to) growth factors such as brain-derived neurotrophic factor (BDNF), cytokines and markers of the inflammatory pathway, and endocrine and metabolic markers.

GENETIC AND EPIGENETIC (DNA) AND TRANSCRIPTOMICS (RNA) EVALUATIONS

The DNA (genetics and epigenetics) and RNA (transcriptomics) component of the study is mandatory. Blood samples for DNA (genetic and epigenetic) and RNA (transcriptomics) will be used for exploratory research related to MDD and relapse. They may also be used to develop tests/assays related to MDD and relapse. Genetic and epigenetic (DNA) and transcriptomics (RNA) research may consist of the analysis of

one or more candidate genes/transcripts or of the analysis of genetic markers throughout the genome (as appropriate) in relation to MDD clinical endpoints, including relapse.

SAFETY EVALUATIONS

Safety evaluations will include:

- Vital signs and weight
- Adverse event monitoring
- Concomitant medications
- Suicide assessment, as measured by the Concise Health Risk Tracking (CHRT) scale. The CHRT has 2 sections:
  - A 16-item subject self-reported screen for suicidal ideation and associated symptoms (Items 1 to 16)
  - A clinician-rated behavioral module, Suicidal Behavioral Evaluation, which covers all Columbia Classification Algorithm of Suicide Assessment (C-CASA) domains (Items 1 to 9) and involves actively querying subjects about the occurrence of suicidal thinking
- Urine pregnancy testing for female subjects of child-bearing potential
- Urine drug screen

STATISTICAL METHODS

Subject Information

For all enrolled subjects, descriptive statistics will be provided.

Sample Size Determination

Assumptions about observed relapses among subjects entering into the study were modeled from the 12-month naturalistic follow-up study of the Sequenced Treatment Alternatives to Relieve Depression (STAR*D) study. In the STAR*D follow-up study, approximately 30% of subjects who entered the 12-month follow-up study were observed to relapse within 6 months. Approximately 46% of subjects entering the follow-up study were observed to relapse within 12 months. Based on these data, for this study, a sample size of approximately 330 subjects is projected to result in 150 subjects who relapse.

A sample size of 330 subjects produces a 2-sided 95% confidence interval with a width equal to 0.108 when the relapse rate is 0.46. For a relapse rate of 0.3, the width of the confidence interval decreases to 0.098 (exact confidence limit according to Wilson).

A total of 150 subjects with observed relapse is a sufficient number of relapses to enable multivariate predictive models, and is based on the rate of relapse observed in the follow-up phase in the STAR*D study. Individual items/measures from self-report and clinician ratings, and summary data from actigraphy assessments (eg, hours of sleep per night), will be used in the development of predictive models. Given the heterogeneity of MDD, predictors of near-term relapse may differ across subgroups of patients; therefore, multiple predictive models of near-term relapse may be calculated across subsets of MDD subjects.

Analysis

Because of the exploratory nature of the study, no hypotheses will be tested. Predictive models of relapse will be developed, especially modeling of relapse using current/most recent assessment scores as covariates (and/or change scores calculated using current/most recent assessments). While the aim is for

prediction of relapse within 1 month or at another identified time point before meeting the criteria for relapse, the time frame for final models will be based on the actual data, and model development will consider various time frames in order to identify the best predictors.

Several exploratory methods will be used to quantify the association between potential risk factors and near-term relapse in subjects with depression.

Descriptive statistics for continuous factors will be presented for subjects by the outcome variable of relapse. Frequency tables of categorical factors will be presented, along with chi-square analyses.

A prediction rule will be developed based on the linear predictor in a logistics regression model in which the outcome variable is near-term relapse.

A non-parametric technique of recursive partitioning will be performed that results in a tree-structured association of covariates with an outcome. The advantage of this technique for the examination of association is the ability to examine conditional interactions in the data, and the resultant decision tree. Missing data are less problematic with this technique than with other multivariate methods because surrogate variables are identified.

Model development will utilize repetitive sampling, by which predictive models can be cross-validated within repeat subsamples.

Analyses of Fluid Biomarkers and Genetic, Epigenetic, and Transcriptomics Data

Changes in Human Discover MAP™ (Multi-Analyte Panel) biomarkers over time will be summarized for relapse (prior to and postrelapse) and nonrelapse groups. In addition, changes in specific biomarker analytes, which may include (but not limited to) growth factors such as brain-derived neurotrophic factor (BDNF), cytokines, and markers of the inflammatory pathway, and endocrine and metabolic markers, will be summarized for the relapse (prior to and postrelapse) and nonrelapse groups.

Genetic, epigenetic, and transcriptomics data will be compared between the relapse (prior to and postrelapse) and nonrelapse groups.

Results will be presented in a separate report.

Healthcare Resource Utilization Analyses

Healthcare resource utilization and work productivity data will be descriptively summarized.

Safety Analyses

The verbatim terms used in the case report form by investigators to identify adverse events will be coded using the Medical Dictionary for Regulatory Activities (MedDRA). All reported adverse events with onset during the observational phase will be included in the analysis. For each adverse event, the percentage of subjects who experience at least 1 occurrence of the given event will be summarized.

Summaries, listings, datasets, or subject narratives may be provided, as appropriate, for those subjects who die, who discontinue the study due to an adverse event, or who experience a severe or a serious adverse event.

Descriptive statistics of pulse/heart rate, blood pressure (systolic and diastolic), and weight values and changes from baseline will be summarized at each scheduled time point. The percentage of subjects with values beyond clinically important limits will be summarized.

Descriptive statistics of changes from baseline to end-of-study/early withdrawal will be summarized for physical examination. Descriptive statistics will be provided for height at screening.

Suicide-related thoughts and behaviors, based on data collected from the CHRT, will be summarized in incidence and shift tables. Separate endpoints for suicidal ideation and suicidal behavior will be defined and summarized descriptively.

TIME AND EVENTS SCHEDULE

Phase	Screening[a]	Observational [b]									
		Baseline[a]	Study Visit [a]							Relapse Verification [c]	End-of-Study/Early Withdrawal [d]
Visit	1	2	3	4	5	6	7	8 [e]	X [e]	--	--
Week (start of)	-2	1	9	17	25	33	41	49	--	--	--
Day	-14	1	57	113	169	225	281	337	--		--
**Study Procedures**											
Screening/Administrative											
Informed consent	X										
Inclusion/exclusion criteria	X										
Medical history and demographics	X										
Personal and family psychiatric history	X										
ETISR-SF [f]	X										
CIRS	X										
MINI	X										
Prestudy therapy	X										
MGH-ATRQ	X										
Dispense smartphone (LogPad®) device and materials		X									
Dispense actigraphy (Actiwatch®) device and materials		X									
Safety Assessments											
Physical examination	X										X
Height	X										
Vital signs,[g] including weight	X	X	X	X	X	X	X	X	X	X	X
CHRT (baseline) [h]	X										
CHRT (since last visit) [h]		X	X	X	X	X	X	X	X	X	X
Urine drug screen[i]	X	X	X	X	X	X	X	X	X		X
Urine pregnancy[j]	X	X	X	X	X	X	X	X	X		X
Clinical Assessments (Clinician)											
MADRS [k]	X	X	X	X	X	X	X	X	X	X	X
CGI-S	X	X	X	X	X	X	X	X	X	X	X
Clinical Assessments (Subject) [f]											
SHAPS [f]		X	X	X	X	X	X	X	X		X
$QIDS\text{-}SR_{16}$ [f]		X	X	X	X	X	X	X	X	X	X

Phase	Screening[a]	Observational [b]									
		Baseline[a]	Study Visit [a]							Relapse Verification [c]	End-of-Study/Early Withdrawal [d]
Visit	1	2	3	4	5	6	7	8 [e]	X [e]	--	--
Week (start of)	-2	1	9	17	25	33	41	49	--	--	--
Day	-14	1	57	113	169	225	281	337	--		--
**Study Procedures**											
GAD-7 [f]		X	X	X	X	X	X	X	X	X	X
MOS Sleep-R [f]		X	X	X	X	X	X	X	X		X
WHODAS 2.0 [f]		X	X	X	X	X	X	X	X		X
EQ-5D-5L [f]		X	X	X	X	X	X	X	X		X
PSS [f]		X	X	X	X	X	X	X	X	X	X
RLCST (baseline) [f]	X										
RLCST (since last visit) [f]		X	X	X	X	X	X	X	X	X	X
P-FIBS [f]		X	X	X	X	X	X	X	X		X
FSR-SA [f]										X[l]	
Depression Implicit Association Test [f]		X	X	X	X	X	X	X	X		X
Speech and voice characteristics[f]		X	X	X	X	X	X	X	X	X	X
Download actigraphy data [m]		X	X	X	X	X	X	X	X	X	X
Healthcare Resource Utilization											
HRUQ [n]		X									
HRUQ (since last visit) [n]			X	X	X	X	X	X	X		X
Fluid Biomarkers											
Blood sample collection		X	X	X	X	X	X	X	X	X[l]	X
Genetics (DNA)											
Blood sample collection [o]		X									
Epigenetics (DNA) and Transcriptomics (RNA)											
Blood sample collection [o]		X								X[l]	X
Adherence to Oral Antidepressant Treatment Regimen											
PAQ [f]		X	X	X	X	X	X	X	X	X	X
Prescription history [p]		X		X		X		X	X	X	X
Ongoing Subject Review											
Concomitant therapy	X	X	X	X	X	X	X	X	X	X	X
Adverse events	X	X	X	X	X	X	X	X	X	X	X

Footnotes appear on the following page.

CGI-S=Clinical Global Impression of Severity. CHRT=Concise Health Risk Tracking. CIRS=Cumulative Illness Rating Scale. EQ-5D-5L=EuroQol Group; 5 dimension; 5 level. ETISR-SF=Early Trauma Inventory Self Report – Short Form. FSR-SA=First Symptom of Relapse – Self-Assessment. GAD-7=Generalized Anxiety Disorder 7-item scale. HRUQ=Health Resource Use Questionnaire. MADRS=Montgomery Asberg Depression Rating Scale. MGH-ATRQ=Massachusetts General Hospital Antidepressant Treatment Rating Questionnaire. MINI=MINI International Neuropsychiatric Interview. MOS Sleep-R=Medical Outcomes Study Sleep Scale, Revised, 12-items. PAQ=Patient Adherence to Antidepressant Medication Questionnaire. P-FIBS=Pain, Frequency, Intensity, and Burden Scale. PSS=Perceived Stress Scale. $QIDS-SR_{16}$=Quick Inventory of Depressive Symptomatology Self-Report. RLCST=Recent Life Changes Stress Test. SHAPS=Snaith Hamilton Pleasure Scale. WHODAS 2.0=World Health Organization Disability Assessment Schedule 2.0.

[a] If feasible, the screening and baseline visits can occur on the same day. Duplicate assessments will not be required if the screening and baseline visits occur on the same day.

[b] The observational phase will be of variable duration, with study visits every 8 weeks until relapse has been observed in 150 subjects. A visit window of ±3 days will be allowed. Refer to the separate Time and Events Schedule: Subject-Completed Assessments (below) for the timing of subject-completed assessments at and between study visits.

[c] If the relapse criterion of MADRS total score ≥22 is met at a study visit (scheduled or unscheduled), an additional visit (ie, Relapse Verification visit) will be scheduled within 1 to 2 weeks to verify the relapse. Subjects who relapse will continue in the observational phase, with study visits every 8 weeks and subject-completed assessments at the frequency shown in the Time and Events Schedule: Subject-Completed Assessments, until relapse has been observed in 150 subjects.

[d] Subjects will complete the end-of-study/early withdrawal visit upon completion of the study (once relapse has been observed in 150 subjects) or at early withdrawal.

[e] Subjects will continue to have study visits every 8 weeks after Week 49 until relapse has been observed in 150 subjects. Visit numbers will continue sequentially (eg, Visit 9, 10, 11, etc).

[f] Subject-reported assessment; some subject-reported assessments will be recorded between study visits. Refer to the Time and Events Schedule: Subject-Completed Assessments (below) for additional detail about the timing of subject-completed assessments at and between study visits.

[g] Vital signs will include blood pressure (supine) and heart rate.

[h] The CHRT includes a subject self-reported module and a clinician-rated behavioral module. At the screening visit, the CHRT baseline visit assessment will be used, which has a 7-day recall period. For all subsequent time points, the CHRT follow-up assessment, which has a recall period of since last visit, will be used.

[i] Urine drug screen to assess for potential substance abuse.

[j] Urine pregnancy test will be performed for all female subjects of childbearing potential.

[k] Administered using the Structured Interview Guide for Montgomery Asberg Depression Rating Scale (SIGMA).

[l] Subjects who relapse but do not require a Relapse Verification visit (ie, they meet relapse criteria other than that based on the MADRS total score) will complete the FSR-SA and have blood samples collected at their next scheduled visit.

[m] Continuous assessment during the observational phase; data from the device will be downloaded at each study visit.

[n] At baseline, subjects will be asked to use a recall period of the prior 6 months; at subsequent study visits, subjects will be asked to recall the period since the last visit.

[o] The DNA (genetics and epigenetics) and RNA (transcriptomics) component of the study is mandatory. The blood sample for genetics should be collected at the specified time point; however, if necessary, it may be collected at a later time point without constituting a protocol deviation. A blood sample for genetic analysis (DNA) will be taken at baseline. Blood samples for epigenetics (DNA) and transcriptomics (RNA) will be taken at baseline, at the Relapse Verification visit, and at end-of-study/early withdrawal.

[p] For assessment of medication adherence, the investigator will document the subject's antidepressant medication prescription history (eg, original date filled, quantity, refill frequency).

TIME AND EVENTS SCHEDULE:SUBJECT-COMPLETED ASSESSMENTS

Phase	Screening[a]	Observational [b]																
Visit	1	Baseline[a] 2								3								4
Week (start of)	-2	1	2	3	4	5	6	7	8	9	10	11	12	13	14	15	16	17
Day	-14	1	8	15	22	29	36	43	50	57	64	71	78	85	92	99	106	113
**Study Procedures**																		
Screening/Administrative																		
ETISR-SF	X																	
Safety Assessments																		
CHRT (subject module) (baseline) [c]	X																	
CHRT (subject module) (since last visit) [c]		X								X								X
Clinical Assessments																		
SHAPS		X								X								X
QIDS-SR$_{16}$		X	X	X	X	X	X	X	X	X	X	X	X	X	X	X	X	X
GAD-7		X	X	X	X	X	X	X	X	X	X	X	X	X	X	X	X	X
MOS Sleep-R		X	X	X	X	X	X	X	X	X	X	X	X	X	X	X	X	X
WHODAS 2.0		X				X				X				X				X
EQ-5D-5L		X		X		X		X		X		X		X		X		X
PSS		X				X				X				X				X
RLCST (baseline)	X																	
RLCST (since last visit)		X								X								X
P-FIBS		X								X								X
Depression Implicit Association Test		X								X								X
Speech and voice characteristics		X		X		X		X		X		X		X		X		X
Adherence to Oral Antidepressant Treatment Regimen																		
PAQ		X	X	X	X	X	X	X	X	X	X	X	X	X	X	X	X	X

Phase	Observational [b]																							
Visit								5								6								7
Week (start of)	18	19	20	21	22	23	24	25	26	27	28	29	30	31	32	33	34	35	36	37	38	39	40	41
Day	120	127	134	141	148	155	162	169	176	183	190	197	204	211	218	225	232	239	246	253	260	267	274	281
**Study Procedures**																								
Safety Assessments																								
CHRT (subject module) (since last visit) [c]								X								X								X
Clinical Assessments																								
SHAPS								X								X								X
QIDS-SR_{16}	X	X	X	X	X	X	X	X	X	X	X	X	X	X	X	X	X	X	X	X	X	X	X	X
GAD-7	X	X	X	X	X	X	X	X	X	X	X	X	X	X	X	X	X	X	X	X	X	X	X	X
MOS Sleep-R	X	X	X	X	X	X	X	X	X	X	X	X	X	X	X	X	X	X	X	X	X	X	X	X
WHODAS 2.0				X				X				X				X				X				X
EQ-5D-5L		X		X		X		X		X		X		X		X		X		X		X		X
PSS				X				X				X				X				X				X
RLCST (since last visit)								X								X								X
P-FIBS								X								X								X
Depression Implicit Association Test								X								X								X
Speech and voice characteristics		X		X		X		X		X		X		X		X		X		X		X		X
Adherence to Oral Antidepressant Treatment Regimen																								
PAQ	X	X	X	X	X	X	X	X	X	X	X	X	X	X	X	X	X	X	X	X	X	X	X	X

Phase	Observational [b]										
										Relapse Verification [d]	End-of-Study/ Early Withdrawal [e]
Visit								8 [f]	X [f]	--	--
Week (start of)	42	43	44	45	46	47	48	49	--	--	--
Day	288	295	302	309	316	323	330	337	--	--	--
**Study Procedures**											
Safety Assessments											
CHRT (subject module) (since last visit) [c]								X	X		X
Clinical Assessments											
SHAPS								X	X		X
QIDS-SR_{16}	X	X	X	X	X	X	X	X	X	X	X
GAD-7	X	X	X	X	X	X	X	X	X	X	X
MOS Sleep-R	X	X	X	X	X	X	X	X	X		X
WHODAS 2.0				X				X	X		X
EQ-5D-5L		X		X		X		X	X		X
PSS				X				X	X	X	X
RLCST (since last visit)								X	X	X	X
P-FIBS								X	X		X
FSR-SA										X [g]	
Depression Implicit Association Test								X	X		X
Speech and voice characteristics		X		X		X		X	X	X	X
Adherence to Oral Antidepressant Treatment Regimen											
PAQ	X	X	X	X	X	X	X	X	X	X	X

Footnotes appear on the next page.

CHRT=Concise Health Risk Tracking. CIRS=Cumulative Illness Rating Scale. EQ-5D-5L=EuroQol Group; 5 dimension; 5 level. ETISR-SF=Early Trauma Inventory Self Report – Short Form. FSR-SA=First Symptom of Relapse – Self-Assessment. GAD-7=Generalized Anxiety Disorder 7-item scale. HRUQ=Health Resource Use Questionnaire. MOS Sleep-R=Medical Outcomes Study Sleep Scale, Revised, 12-items. PAQ=Patient Adherence to Antidepressant Medication Questionnaire. P-FIBS=Pain, Frequency, Intensity, and Burden Scale. PSS=Perceived Stress Scale. $QIDS\text{-}SR_{16}$=Quick Inventory of Depressive Symptomatology Self-Report. RLCST=Recent Life Changes Stress Test. SHAPS=Snaith Hamilton Pleasure Scale. WHODAS 2.0=World Health Organization Disability Assessment Schedule 2.0.

[a] If feasible, the screening and baseline visits can occur on the same day. Duplicate assessments will not be required if the screening and baseline visits occur on the same day.

[b] The observational phase will be of variable duration, with study visits every 8 weeks until relapse has been observed in 150 subjects. A visit window of ±3 days will be allowed for study visits. A window of ±1 day will be allowed for subject-completed assessments between study visits.

[c] The CHRT includes a subject self-reported module and a clinician-rated behavioral module. At the screening visit, the CHRT baseline visit assessment will be used, which has a 7 day recall period. For all subsequent time points, the CHRT follow-up assessment, which has a recall period of since last visit, will be used.

[d] If the relapse criterion of MADRS total score ≥22 is met at a study visit (scheduled or unscheduled), an additional visit (ie, Relapse Verification visit) will be scheduled within 1 to 2 weeks to verify the relapse. Subjects who relapse will continue in the observational phase, with study visits every 8 weeks and subject-completed assessments at the frequency shown, until relapse has been observed in 150 subjects.

[e] Subjects will complete the end-of-study/early withdrawal visit upon completion of the study (once relapse has been observed in 150 subjects) or at early withdrawal.

[f] Subjects will continue to have study visits every 8 weeks after Week 49 until relapse has been observed in 150 subjects. Visit numbers will continue sequentially (eg, Visit 9, 10, 11, etc). Subject assessments will continue to be administered at the same frequency during the additional weeks.

[g] Subjects who relapse but do not require a Relapse Verification visit (ie, they meet relapse criteria other than that based on the MADRS total score) will complete the FSR-SA at their next scheduled visit.

ABBREVIATIONS

ADR	adverse drug reaction
CGI-S	Clinical Global Impression of Severity
CHRT	Concise Health Risk Tracking
CIRS	Cumulative Illness Rating Scale
CRF	case report form
DSM-5	Diagnostic and Statistical Manual of Mental Disorders (5th edition)
eDC	electronic data capture
EQ-5D-5L	EuroQol Group; 5 dimension; 5 level
ETISR-SF	Early Trauma Inventory Self Report – Short Form
FSR-SA	First Symptom of Relapse – Self-Assessment.
GAD-7	Generalized Anxiety Disorder 7-item scale
GCP	Good Clinical Practice
HRUQ	Health Resource Use Questionnaire
IAT	Implicit Association Test
ICF	informed consent form
ICH	International Conference on Harmonisation
IEC	Independent Ethics Committee
IRB	Institutional Review Board
MADRS	Montgomery Asberg Depression Rating Scale
MDD	major depressive disorder
MDE	major depressive episode
MedDRA	Medical Dictionary for Regulatory Activities
MGH-ATRQ	Massachusetts General Hospital Antidepressant Treatment Response Questionnaire
MINI	MINI International Neuropsychiatric Interview
MOS Sleep-R	Medical Outcomes Study Sleep Scale, Revised, 12-items
QIDS-SR_{16}	Quick Inventory of Depressive Symptomatology Self-Report Scale
PAQ	Patient Adherence to Antidepressant Medication Questionnaire.
P-FIBS	Pain, Frequency, Intensity, and Burden Scale
PRO	patient-reported outcome(s)
PQC	product quality complaint
PSS	Perceived Stress Scale
RLCST	Recent Life Changes Stress Test.
SHAPS	Snaith Hamilton Pleasure Scale
SIGMA	Structured Interview Guide for Montgomery Asberg Depression Rating Scale
STAR*D	Sequenced Treatment Alternatives to Relieve Depression
WHODAS 2.0	World Health Organization Disability Assessment Schedule 2.0

1. INTRODUCTION

1.1. Background

Major depressive disorder (MDD) is one of the leading causes of disability in adults worldwide (measured as years lived with disability),[69] with a lifetime prevalence of approximately 15% in the general adult population, and is associated with significant morbidity and mortality. Although a number of treatments are available for MDD, unfortunately response to treatment is often poor. As such, the disorder has a significant negative impact on functioning, even in treated patients.

In most cases, MDD is a chronic disorder with recurrent episodes. Relapse (within the same episode) or recurrence (start of a new episode) typically occurs gradually over weeks. There is limited information known about precursors to, or predictors of, relapse or recurrence. The course of improvement from an acute depressive episode to remission is better known from short-term studies. However, imminent precursors to relapse are not as well known, since patients often seek medical attention only after relapse has occurred. In current clinical practice, clinicians monitor a patient's symptoms over time and use this information as a guide to their clinical management, making changes in a patient's treatment regimen as needed. Using this reactive approach, the treating clinician is often unaware of early changes in the patient's symptomatology. Relapse or recurrence is frequently only detected after a patient's depressive symptoms have worsened enough to warrant an assessment at the office or clinic.

There is very limited knowledge with regard to changes in MDD patient status during continuation/maintenance treatment that may be indicative of early stages of a relapse prodrome or impending relapse. While, for example, residual symptoms present at exit from antidepressant treatment studies for an acute major depressive episode (MDE) have been studied as predictors of relapse at any point during 1-year follow-up maintenance antidepressant treatment,[44] few studies have examined markers that may change or appear during continuation/maintenance treatment that may be more immediate precursors to relapse. If markers of a likely relapse within a period of weeks can be identified, then clinicians might conduct further clinical evaluation and adjust treatment as appropriate for patients who exhibit such markers of impending relapse. In this study, analyses will attempt to identify changes/markers of relapse within several weeks to 1 month; various time frames will be explored in predictive modeling, and final models will be considered based on performance. Identification of markers of impending MDD relapse may enable strategies to intercept disease worsening, eg, (re)introduction of rapidly acting antidepressants or other appropriate treatment interventions.

The term "sponsor" used throughout this document refers to the entities listed in the Contact Information page(s), which will be provided as a separate document.

1.2. Overall Rationale for the Study

The purpose of the current prospective, longitudinal, observational study is to determine whether there are measures predictive of relapse in MDD. The study will assess several parameters that may predict relapse (within 1 month or at another identified time point before meeting the criteria for relapse) in subjects with MDD who, within 3 months before entering the study, have

responded to and are currently continuing to maintain their response to, an oral antidepressant treatment regimen. A change in the oral antidepressant treatment regimen since achieving response in the current episode will be allowed. Statistical models of near-term relapse (within 1 month or at another identified time point before meeting the criteria for relapse) and/or symptomatic change indicative of relapse prodrome in MDD will be developed. If predictive modeling analytics are shown to be able to identify changes that are reliable signals of relapse within 1 month or at another identified time point, then patients who may potentially benefit from earlier clinical intervention can be more reliably identified. To this end, such predictive models of relapse could be used to inform the design and/or selection of clinical assessments in future MDD studies. In addition, model outputs may contribute to the design of tools for clinicians and patients to track treatment response.

The study will be observational in nature, and the intent is that participants will be treated for their depression as per standard of care during the study. Assessments will include self-reported ratings of mood, anxiety, social and occupational function, health-related quality of life, and sleep, and objective assessments of motor activity, sleep, and speech and voice parameters. Self-report and behavioral (speech) data will be captured via applications on a study-specific smartphone (LogPad®) provided to subjects, and activity and objective sleep measurements will be recorded via a wrist actigraphy device (Actiwatch®). Assessment of subject's adherence to their oral antidepressant treatment regimen will be assessed by a subject self-assessment and by the investigator's documenting the subject's antidepressant medication prescription history (eg, original date filled, quantity, refill frequency), since this is an important factor to consider when assessing prediction of relapse. Information on concomitant therapy will be captured at each study visit. In addition, if a change is made to the subject's antidepressant medication, this must be documented, along with the reason for the change. Blood samples will be collected to explore if there are any associated biomarkers that may predict relapse.

2. OBJECTIVES

Primary Objective

The objective of this study is to identify if there are self-reported or objective measures related to mood parameters that can predict near-term relapse (within 1 month or at another identified time point before meeting the criteria for relapse) or early symptomatic changes indicative of relapse prodrome in MDD. Domains to be assessed include mood and anxiety symptoms, stress, sleep, motor activity, pain, health, functioning, and quality of life, implicit cognitive processes, compliance with the oral antidepressant treatment regimen, and speech and voice characteristics.

This is an exploratory prospective study to derive a predictive model(s) of relapse in MDD. Rather than testing of specific hypotheses about particular variables, a range of variables will be measured and evaluated as potential predictors of relapse, including depressive and anxiety symptoms, health, functioning, and quality of life, sleep, adherence to antidepressant treatment regimen, behavior/activity, and speech, observed either via remote monitoring or obtained at regular study visits.

3. STUDY DESIGN AND RATIONALE

3.1. Overview of Study Design

This is a prospective, multicenter, longitudinal, single-cohort, observational study in subjects with MDD who have responded to, and are continuing to respond to and receive, an oral antidepressant treatment regimen. Approximately 330 subjects will be enrolled in order to result in 150 subjects who relapse. The study will consist of 2 phases: a screening phase of up to 2 weeks, and an observational phase of variable duration, with study visits every 8 weeks until relapse has been observed in 150 subjects. The total study duration for each subject will therefore be variable. Subjects who relapse will continue in the observational phase, with study visits every 8 weeks, until relapse has been observed in 150 subjects (ie, the intent is to follow all relapsed and non-relapsed subjects in the observational phase). Relapse will only be counted once for any given subject. The end of the study will be defined as the date of the last visit of the last subject participating in the study, once relapse has been observed in 150 subjects.

Once relapse has been observed in 150 subjects, all subjects will conclude study participation with an end-of-study visit conducted at the next scheduled study visit (ie, 8-week interval), and will then be referred for further follow-up with their treating physician.

To be eligible to enter the study, a subject must have met Diagnostic and Statistical Manual of Mental Disorders (5th edition) (DSM-5) diagnostic criteria for nonpsychotic, recurrent MDD within the past year (ie, the start of the current MDE must be ≤1 year before screening), but must not currently meet criteria for MDD; have a MADRS total score ≤14; and have evidence of recent response (within the past 3 months) to an oral antidepressant treatment regimen (taken at an optimal dosage and for an adequate duration), and be currently taking an oral antidepressant treatment regimen. A change in the oral antidepressant treatment regimen since achieving response in the current episode will be allowed. All antidepressant medication taken for the current MDE will be captured on the Massachusetts General Hospital Antidepressant Treatment Response Questionnaire (MGH-ATRQ), with corroboration from medical and pharmacy records, if available.

After consenting to participate in the study, subjects will undergo a brief diagnostic interview (MINI International Neuropsychiatric Interview; MINI - DSM-5) to confirm that the diagnosis of MDD was present prior to their antidepressant response, and to determine if there are other psychiatric conditions present.

At baseline, subjects will complete assessments of mood, as well as other assessments as outlined in the Time and Events Schedule. All participants will receive a study-specific smartphone (LogPad®), which they will use to complete assessments at and between study visits. Motor activity and sleep parameters will be captured using actigraphy via a wrist-worn device throughout the study period. Subjects will receive text reminders to complete questionnaires via the study-provided smartphone. Clinicians will also remind subjects at study visits of the importance of completing self-report measures. Subjects will continue to participate in treatment as directed by their clinician, and will return for visits to the clinic every 8 weeks.

Relapse criteria are defined in Section 9.2.2. Subjects who relapse will continue in the observational phase until relapse has been observed in 150 subjects. Subjects will continue to participate in routine study visits at 8-week intervals as well as continuing assessments between study visits using self-report measures of symptoms, antidepressant medication adherence, functioning, health-related quality of life, and sleep. Appropriate adjustment in antidepressant treatment will be made by the investigator as needed.

An Adjudication Committee will be commissioned for this study, to adjudicate any cases of relapse where it is unclear if relapse occurred or where further discussion is needed. Refer to Section 11.7, Adjudication Committee, for details.

Blood samples will be collected from all subjects (where local regulations permit) to allow for genetic, epigenetic, and transcriptomic research to help understand MDD and factors relating to relapse.

A diagram of the study design is provided in Figure 1.

Figure 1. Schematic Overview of Study OBSERVEMDD0001

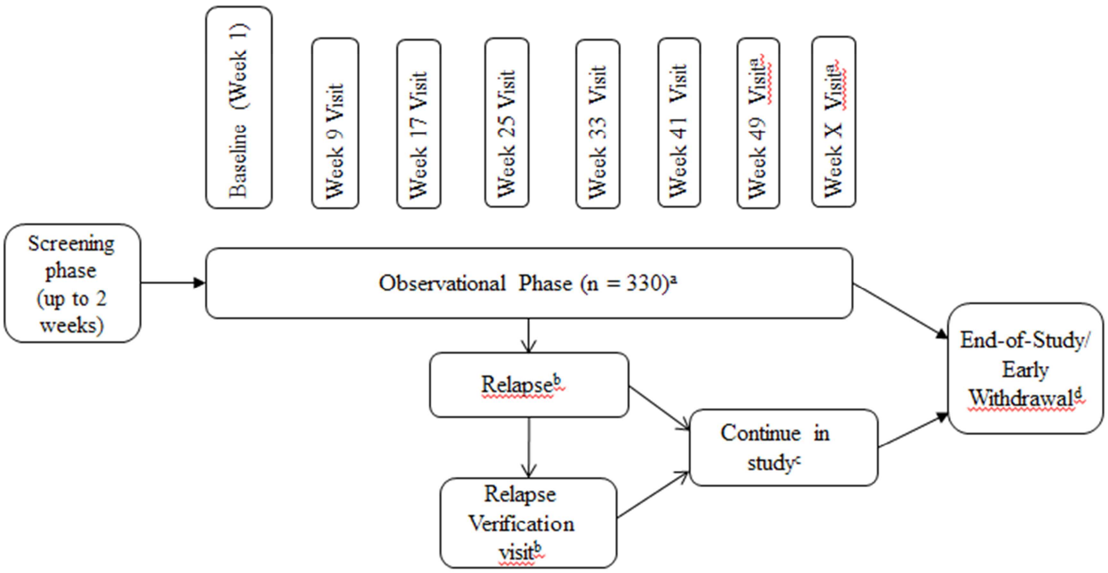

[a] The observational phase will be of variable duration, with study visits every 8 weeks until relapse has been observed in 150 subjects. Subjects will continue to have study visits every 8 weeks after Week 49 until relapse has been observed in 150 subjects.
[b] If the relapse criterion of MADRS total score ≥22 is met at a study visit (scheduled or unscheduled), an additional visit (ie, Relapse Verification visit) will be scheduled within 1 to 2 weeks to verify the relapse.
[c] Subjects who relapse will continue in the observational phase, with study visits every 8 weeks and subject-completed assessments at the frequency shown in the Time and Events Schedule: Subject-Completed Assessments, until relapse has been observed in 150 subjects.
[d] Subjects will complete the end-of-study/early withdrawal visit upon completion of the study (once relapse has been observed in 150 subjects) or at early withdrawal.

3.2. Study Design Rationale

This study is a prospective, multicenter, longitudinal, single-cohort, observational study of subjects 18 to 64 years of age, inclusive, with a history of nonpsychotic, recurrent MDD who have recently (within the prior 3 months) responded to, and are continuing to respond to and

receive, an oral antidepressant treatment regimen. The requirement of having a MADRS total score ≤14 ensures that a subject's level of depressive symptoms at screening are consistent with response.

The study is intended to provide information on potential predictors of near-term relapse (within 1 month or at another identified time point before meeting the criteria for relapse) in MDD. The duration of 1 month was chosen as it is considered to be a clinically reasonable timeframe; if clinicians could detect deterioration during this time frame, it could potentially avert relapse. However, as discussed in Section 11.3, this potentially can be adjusted based on the actual data. If the data suggest, for example, that one can predict relapse within 6 weeks or 8 weeks, this information will be ascertained. Analysis of study data will include the development of predictive models of clinical relapse, with the goal to enable better identification of MDD patients who may be at increased risk of near-term relapse.

The subject population is intended to be representative of MDD patients who have recently responded to antidepressant treatment and are being treated with continuation antidepressant medications. In order to decrease heterogeneity of the target population, the duration of the current MDE must not exceed 1 year (ie, the start of the current MDE must be ≤1 year before screening).

Approximately 330 subjects will be enrolled in the study and will be followed until relapse has been observed in 150 subjects. The observational phase will be of variable duration, with study visits every 8 weeks until relapse has been observed in 150 subjects. At that time, all ongoing subjects will return for an end-of-study visit, and will then be referred for further follow-up with their treating physician. The number of subjects to be enrolled is believed to be sufficient to obtain the required number of subjects (ie, 150) who relapse, and is based on the rate of relapse observed in the naturalistic follow-up phase in the Sequenced Treatment Alternatives to Relieve Depression (STAR*D) study.[52]

The STAR*D study consisted of a broadly representative adult outpatient sample of subjects with nonpsychotic MDD who received one (N=3,671) to four (N=123) successive acute treatment steps.[52] Subjects were initially treated with citalopram; those who did not achieve remission or were unable to tolerate the medication were encouraged to advance to other medications sequentially until treatment response was attained. Those with an acceptable benefit, preferably symptom remission, from any particular step and tolerated acute treatment could enter a longer-term, 12-month naturalistic follow-up phase, as could those with at least a meaningful improvement and acceptable tolerability. Inclusion and exclusion criteria for the present study are modeled after the STAR*D criteria.

A score of ≤5 on the Quick Inventory of Depressive Symptomatology Self-Report Scale ($QIDS\text{-}SR_{16}$) (equivalent to ≤7 on the 17-item Hamilton Rating Scale for Depression; $HRSD_{17}$) defined remission; a $QIDS\text{-}SR_{16}$ total score of ≥11 ($HRSD_{17}$ ≥14) defined relapse. The $QIDS\text{-}SR_{16}$ remission rates were 36.8%, 30.6%, 13.7%, and 13.0% for the first, second, third, and fourth acute treatment steps, respectively. The overall cumulative remission rate was 67%. Overall, those who required more treatment steps had higher relapse rates during the naturalistic

follow-up phase. In addition, lower relapse rates were found among participants who were in remission at follow-up entry than for those who were not after the first 3 treatment steps (Table 1).

Table 1: STAR*D Study: Remission Status at Follow-Up Entry and Relapse Rates for Participants Entering Follow-Up From Each Treatment Step

Treatment Step and Remission Status at Follow-Up Entry	Number Entering Follow-Up Phase	Remission Rate at Follow-Up Entry (%) [a]	$QIDS\text{-}SR_{16}$ Score at Entry [b]	Number With at Least One Postbaseline Contact [c]	Relapse Rate (%) [d]	Months to Relapse (of Those Relapsing)
Step 1 (N=3,671) [e]	1,475	73.7	4.0	1,133	40.1	4.1
In remission	1,085		2.7	841	33.5	4.4
Not in remission	388		7.7	290	58.6	3.6
Step 2 (N=1,439) [e]	622	61.8	5.1	479	55.3	3.9
In remission	383		3.0	291	47.4	4.5
Not in remission	237		8.3	186	67.7	3.2
Step 3 (N=390) [e]	102	34.7	6.8	79	64.6	3.1
In remission	35		3.3	28	42.9	3.9
Not in remission	66		8.6	50	76.0	3.0
Step 4 (N=123) [e]	49	30.6	8.3	38	71.1	3.3
In remission	15		3.3	14	50.0	2.5
Not in remission	34		10.5	24	83.3	3.5

[a] All treatment step pairwise comparisons significant at $p<0.0001$ except for Step 3 versus Step 4 ($p<0.63$).
[b] All treatment step pairwise comparisons significant at $p<0.0001$ except for Step 3 versus Step 4 ($p<0.05$).
[c] Subjects who made at least one call to the interactive voice response system.
[d] Proportion of subjects relapsing as a percentage of those who made at least one postbaseline call to the interactive voice response system. Treatment step pairwise comparisons showed only Step 1 to be significantly different from the rest ($p<0.0001$).
[e] N's represent the number of subjects who entered the step.
From Rush 2006.[52]

In the current study, subjects will participate in frequent assessments using self-report measures of symptoms, antidepressant medication adherence, functioning, health-related quality of life, and sleep. Clinician ratings of symptom severity will be collected at routine study visits (8-week intervals). The selection of parameters, and their associated frequency of administration, was based on review of the literature and clinical judgment, in order to include those parameters (and assess them at an appropriate frequency) that would be considered to be most relevant to this patient population (see Selection of Clinical Assessments, below, for a more detailed discussion of the parameters chosen for this study). The interval of 8 weeks for study visits is considered to be consistent with standard clinical practice. Additional measures will include ongoing assessment of activity and sleep quantity via actigraphy. Subjects will provide speech and voice samples for evaluation using the LogPad. In addition, since antidepressant treatment compliance is an important factor to consider, subjects' adherence to their oral antidepressant treatment regimen will be assessed with a self-report assessment of medication adherence, completed weekly, and with documentation by the investigator, at each study visit, of the subject's antidepressant medication prescription history (eg, original date filled, quantity, refill frequency).

All of the parameters included are hypothesized to potentially change with worsening of clinical status within 1 month or at another identified time point before meeting the criteria for relapse. Relapse will be defined using multiple criteria, including clinician rating (MADRS), hospitalization, and suicidal ideation or behavior (see Section 9.2.2). The relapse criteria used are consistent with criteria used in clinical studies in this patient population.[40,50]

Subjects who relapse will continue in the observational phase, with study visits every 8 weeks, until relapse has been observed in 150 subjects. Appropriate adjustment in treatment will be made by the investigator as needed and subjects will continue to participate in frequent assessments using self-report measures of symptoms, antidepressant medication adherence, functioning, health-related quality of life, and sleep as well as attending routine study visits at 8-week intervals.

The primary analysis will include multivariate modeling of relapse, especially models using data from assessments performed approximately 1 month before meeting the criteria for relapse. A specific aim of the study is to develop an algorithm(s) to predict near-term clinical relapse in MDD, by which patients who may be at increased risk of near-term relapse might be identified. A sample size of 330 subjects is projected to result in relapse in 150 subjects.[52] This is a sufficient number of relapses to enable multivariate predictive models.

Selection of Clinical Assessments

Selection of the clinical assessments included in the study was guided by clinical judgment and literature review of the parameters likely to change as precursors of clinical relapse, as shown in Table 2. The table provides the specific parameters and domains to be assessed, with the instrument/measure to be used, the frequency, and the rationale.

Table 2. Clinical Assessments, by Parameter and Domain

Domain	Rationale for Assessment	Instrument/Measure	Timing	Comments
MDD symptoms:	Changes in symptoms may be markers of relapse or relapse prodrome	MADRS	Study visits (8-week intervals)	MADRS provides clinician ratings of MDD symptoms and severity, and will be used as part of the relapse criteria.
		CGI-S	Study visits (8-week intervals)	CGI-S is a low-burden assessment and provides an overall clinician rating of severity.
		$QIDS\text{-}SR_{16}$ [a]	Weekly	$QIDS\text{-}SR_{16}$ is the primary patient-reported measure of depression symptoms.
Anxiety symptoms	Anxiety symptoms are frequently associated symptoms of MDD, and require further study for prognostic relevance.[52]	GAD-7 [a]	Weekly	GAD-7 is the primary patient-reported measure of anxiety symptoms.
Sleep disturbance	Sleep disturbances and/or changes in sleep quantity/quality may be particularly likely to predict impending relapse.[44] Self-report and objective measures of sleep quantity/quality may be poorly correlated.[26]	MOS Sleep-R [a]	Weekly	MOS Sleep-R will provide more detailed information on subjective sleep behavior. The instrument yields 6 subscales: sleep disturbance, snoring, shortness of breath or headache, sleep adequacy, sleep somnolence, and sleep quantity.
		Actigraphy	Continuous monitoring	Objective data on sleep quantity and quality will be measured via actigraphy.
Anhedonia	Anhedonia is among the criterion symptoms for MDD, may differentiate among MDD subtypes, and is predictive of antidepressant response.[57,73]	SHAPS [a]	Study visits (8-week intervals)	The SHAPS is a brief, self-report measure providing information on a key symptom of depression – ie, loss of pleasure. The SHAPS covers 4 domains of hedonic experience: interest/pastimes, social interaction, sensory experience, and food/drink.
Antidepressant medication adherence	Antidepressant adherence is a predictor of time to relapse in MDD.[1]	Antidepressant medication prescription history	Study visits (16-week intervals)	The investigator will document the subject's antidepressant medication prescription data. This information will be used in calculating the medication possession ratio (MPR), a method of assessing medication adherence.[24]
		PAQ [a]	Weekly	The PAQ is a brief, 2-item questionnaire that provides information from the subject regarding medication compliance.

Table 2. Clinical Assessments, by Parameter and Domain

Domain	Rationale for Assessment	Instrument/Measure	Timing	Comments
Speech and voice parameters	Speech rate, voice markers correlate with MDD severity.[61]	Speech and voice samples	2-week intervals	Collected via LogPad.
Mood-related cognition	"Automatic evaluative biases" towards negative self-categorization in subjects with a history of MDD predict recurrence of MDEs.[14]	Depression IAT	Study visits (8-week intervals)	Primary score(s) = response latency; accurate scoring of latency requires that IAT be administered in controlled environment (ie, clinic visits).
Functioning/ disability	MDD is associated with considerable disability and disruption of life activities; level of disability increases with symptom severity.[3] As rapid-acting antidepressants become available, functional changes may be seen in shorter timeframes than previously explored.	WHODAS 2.0 [a]	4-week intervals	The WHODAS 2.0 assesses the following 6 domains: cognition, mobility, self-care, getting along (interacting with other people), life activities, and participation.
Health-related quality of life	Quality of life is differentially responsive to antidepressant treatment during acute vs. maintenance/ continuation treatment.[46]	EQ-5D-5L [a]	2-week intervals	The EQ 5D 5L is a brief scale that is used increasingly by stakeholders for health economic assessment. It provides a descriptive system comprising the following 5 dimensions: mobility, self-care, usual activities, pain/discomfort, and anxiety/depression.
Pain	Pain severity predicts antidepressant outcomes.[2]	P-FIBS [a]	Study visits (8-week intervals)	Pain is a common associated symptom of MDD. The P-FIBS is a brief, low-burden assessment.
Additional subject perspective	Subject self-insights regarding the first sign, symptom, or indication that may precede relapse	FSR-SA [a]	At Relapse Verification visit[b]	
Healthcare utilization	Increased healthcare utilization may precede MDD relapse. Healthcare utilization is often evaluated by stakeholders for health economic assessment.	HRUQ	Study visits (8-week intervals)	

Table 2. Clinical Assessments, by Parameter and Domain

Domain	Rationale for Assessment	Instrument/Measure	Timing	Comments
Stress/resilience	Life stress, and perceived stressors, have been implicated in MDD onset and recurrence.[43]	PSS [a]	4-week intervals	In addition to evaluating levels of stress, captured data on stressors may be informative.
		RLCST [a]	Study visits (8-week intervals)	

CGI-S=Clinical Global Impression of Severity. CHRT=Concise Health Risk Tracking. CIRS=Cumulative Illness Rating Scale. EQ-5D-5L=EuroQol Group; 5 dimension; 5 level. ETISR-SF=Early Trauma Inventory Self Report – Short Form. FSR-SA=First Symptom of Relapse – Self-Assessment. GAD-7=Generalized Anxiety Disorder 7-item scale. hMAP=Human Discovery Multi-Analyte Panel. HRUQ=Health Resource Use Questionnaire. MADRS=Montgomery Asberg Depression Rating Scale. MGH-ATRQ=Massachusetts General Hospital Antidepressant Treatment Rating Questionnaire. MINI=MINI International Neuropsychiatric Interview. MOS Sleep-R=Medical Outcomes Study Sleep Scale, Revised, 12-items. PAQ=Patient Adherence to Antidepressant Medication Questionnaire. P-FIBS=Pain, Frequency, Intensity, and Burden Scale. PSS=Perceived Stress Scale. QIDS-SR_{16}=Quick Inventory of Depressive Symptomatology Self-Report. RLCST=Recent Life Changes Stress Test. SHAPS=Snaith Hamilton Pleasure Scale. WHODAS 2.0=World Health Organization Disability Assessment Schedule 2.0.

[a] Subject-reported assessment; some subject-reported assessments will be recorded between study visits.

[b] Subjects who relapse but do not require a Relapse Verification visit (ie, they meet relapse criteria other than that based on the MADRS total score) will complete the FSR-SA at their next scheduled visit.

Fluid Biomarker Collection

Blood samples will be collected to allow for the exploratory pharmacodynamic evaluation using the Human Discovery MAP™ (Multi-Analyte Panel), a panel of approximately 250 proteins involved in key biologically relevant pathways associated with major depression, including neurotrophic and inflammatory factors. In addition, analysis of specific biomarker analytes may include (but not limited to) growth factors such as brain-derived neurotrophic factor (BDNF), cytokines and markers of the inflammatory pathway, and endocrine and metabolic markers.

The goal of the research is to identify potential candidate biomarkers that may have utility in evaluating prognosis of disease progression and predicting relapse.[29] There is evidence that certain biomarkers may have predictive value in the relapse of MDD,[53,54] including serum BDNF (protein),[38] inflammation markers,[12,28,58,66] and a glial marker.[55]

Genetic and Epigenetic (DNA) and Transcriptomic (RNA) Collection

It is recognized that genetic variation can be an important contributory factor to interindividual differences in drug distribution and response and can also serve as a marker for disease susceptibility and prognosis. The goal of the genetic and epigenetic component is to collect DNA to allow the identification of genetic and epigenetic factors that may influence the potential for relapse and to identify those associated with MDD. Similarly, the goal of the transcriptomic component is to collect RNA to allow for the identification of transcripts that may influence the potential for relapse. [48]

The heritability of MDD is estimated to be 31% to 42%,[60] implicating the role of genetic variants in MDD disease susceptibility. Candidate variants such as variants from NR2B,[74] GRIK4,[45] HTR2A,[33] BDNF,[20,25,27] SLC6A4,[27] P2RX7,[32] IL6,[8] and IL6R[67] have been reported for either disease susceptibility or antidepressant treatment response, although a robust genetic marker has yet to be unequivocally demonstrated. Likewise, genome-wide association studies[13,21,31,64] and, increasingly, sequencing approaches have been used to identify genetic variants of interest using a hypothesis-free approach. Collection of blood samples will enable the study of both genetic variability and epigenetic changes,[16,17] and their relationship with MDD and antidepressant treatment response, including relapse/recurrence.

Healthcare Resource Utilization

Continued response to the current antidepressant medication(s) is expected to result in low utilization of services, whereas impending relapse is expected to result in higher utilization of healthcare services (such as outpatient visits, emergency room visits, or hospitalization), and potentially impaired work productivity, as assessed periodically using the Health Resource Utilization Questionnaire (HRUQ). The HRUQ includes information regarding utilization of healthcare services since the last clinic visit, including the timing of services, enabling changes in level and quantity of services to be considered as a variable in predictive models.

4. SUBJECT POPULATION

The inclusion and exclusion criteria for enrolling subjects in this study are described in the following 2 subsections. If there is a question about the inclusion or exclusion criteria below, the investigator should consult with the appropriate sponsor representative before enrolling a subject in the study.

4.1. Inclusion Criteria

Each potential subject must satisfy all of the following criteria to be enrolled in the study.

1. Subject must be a man or woman 18 to 64 years of age, inclusive.

2. Subject must have met DSM-5 criteria for diagnosis of nonpsychotic MDD within the past year (ie, the start of the current MDE must be ≤1 year before screening), as confirmed using the MINI International Neuropsychiatric Interview (MINI – DSM-5). MDD must be recurrent, rather than a single episode.

3. In the current MDE, subject must have responded to, and must be continuing to receive and respond to, an oral antidepressant treatment regimen (given at an adequate dosage and duration based on the MGH-ATRQ), within the past 3 months. A change in the oral antidepressant treatment regimen since the time of achieving response in the current episode will be allowed.

4. Subject must have a MADRS total score ≤14.

5. Subject must be willing and able to complete self-reported assessments via a study-specific smartphone (LogPad®), and must be willing to wear an Actiwatch for the duration of the study.

6. Each subject must sign an informed consent form (ICF) indicating that he or she understands the purpose of and procedures required for the study and is willing to participate in the study.

4.2. Exclusion Criteria

Any potential subject who meets any of the following criteria will be excluded from participating in the study.

1. Subject has any of the following DSM-5 psychiatric diagnoses: MDD with psychotic features (lifetime), bipolar disorder (including lifetime diagnosis), schizophrenia, or schizoaffective disorder.

2. Subject has a history of drug or alcohol use, with a severity of at least moderate or severe, according to DSM-5 criteria, within 6 months before screening.

3. Subject has received an investigational drug (including investigational vaccines) or used an invasive investigational medical device within 90 days before screening or is currently enrolled in an investigational study.

4. Subject is currently receiving or has received vagal nerve stimulation (VNS), electroconvulsive therapy (ECT), or transcranial magnetic stimulation (TMS) for the current MDE.

5. Subject is currently receiving stimulants, anticonvulsants, or mood stabilizers for treatment of his or her MDD.

6. Subject is a woman who is pregnant, or planning to become pregnant, while enrolled in this study.

7. Subject has any condition for which, in the opinion of the investigator, participation would not be in the best interest of the subject (eg, compromise the well-being) or that could prevent, limit, or confound the protocol-specified assessments.

8. Subject is an employee of the investigator or study site, with direct involvement in the proposed study or other studies under the direction of that investigator or study site, or is a family member of an employee or the investigator.

NOTE: Investigators should ensure that all study enrollment criteria have been met at screening. If a subject's status changes (including receipt of additional medical records) after screening but before the baseline visit such that he or she no longer meets all eligibility criteria, then the subject should be excluded from participation in the study. Section 15.4, Source Documentation, describes the required documentation to support meeting the enrollment criteria.

5. TREATMENT ALLOCATION AND BLINDING

Subjects will receive no study-specific treatment in this unblinded, non-interventional, observational, naturalistic study.

6. DOSAGE AND ADMINISTRATION

Study subjects will continue to receive their oral antidepressant treatment regimen per their treating physician/clinician during the study. Adjustment to the oral antidepressant treatment regimen (if applicable) will be allowed as clinically indicated, including if a subject relapses, and will be documented in the case report form. The investigator will be required to document why a change was made to the oral antidepressant treatment regimen.

7. ANTIDEPRESSANT TREATMENT COMPLIANCE

Subjects' adherence to their oral antidepressant treatment regimen will be assessed in 2 ways:

- The Patient Adherence to Antidepressant Medication Questionnaire (PAQ) is a brief, 2-item self-report scale, developed at the University of Texas Southwestern Medical Center,[41] to

assess how often the subject has taken, and whether he/she has made any changes to, his/her antidepressant treatment regimen in the last week. The total score will be calculated by adding response choices for questions 1c through 1f, with 0=adherent and 1 or more=non-adherent. An example of the PAQ is provided in Attachment 1.

- The investigator will document the subject's antidepressant medication prescription history (eg, original date filled, quantity, refill frequency). This information will be used in calculating the medication possession ratio (MPR), a method of assessing medication adherence.[24]

8. PRESTUDY AND CONCOMITANT THERAPY

Prestudy therapies administered up to 30 days before screening must be recorded in the CRF.

All antidepressant therapies, including adjunctive treatment for MDD, used and stopped >30 days prior to screening, either in the current or prior (if available, known from the subject's psychiatric history or verbal report) depressive episodes that are not continuing at screening, must be recorded in the CRF.

Antidepressant medication(s) taken for the current MDE and associated treatment response will be documented in the MGH-ATRQ and in appropriate sections of the CRF.

Concomitant medications are allowed to manage concurrent general medical conditions or antidepressant side effects (eg, sexual dysfunction). Anxiolytics and sedative hypnotics (including trazodone 200 mg/day for sleep) will be allowed for sleep.

Concomitant therapies must be recorded throughout the study beginning with the screening visit. Concomitant therapies should also be recorded 30 days after the end of the study only in conjunction with new or worsening adverse events or serious adverse events that meet the criteria outlined in Section 12.1.1. Adverse Event Definitions and Classifications.

All therapies (prescription or over-the-counter medications, including vaccines, vitamins, herbal supplements; non-pharmacologic therapies such as psychotherapy, acupuncture, special diets, exercise regimens) must be recorded in the CRF. Recorded information will include a description of the type of the drug therapy, treatment period, dosing regimen, route of administration, and its indication. Any change to the subject's antidepressant medication must be documented, along with the reason for the change. Modification of an effective preexisting therapy should not be made for the explicit purpose of entering a subject into the study.

9. STUDY EVALUATIONS

9.1. Study Procedures

9.1.1. Overview

The Time and Events Schedule summarizes the frequency and timing of efficacy, biomarker, pharmacogenomic, medical resource utilization, and safety measurements applicable to this study. In addition, the Time and Events Schedule: Subject-Completed Assessments provides the timing for subject-completed assessments at and between study visits.

All visit-specific patient-reported outcome (PRO) assessments should be conducted/completed before any tests, procedures, or other consultations for that visit to prevent influencing subject perceptions. Subject-reported assessments should be completed in the sequence provided on the SitePad and LogPad:

- Screening (SitePad):
  - ETISR-SF
  - CHRT (subject module)
- Observational phase (SitePad):
  - SHAPS
  - P-FIBS
  - Recent Life Changes Stress Test (RLCST)
  - Depression IAT
  - CHRT (subject module)
- Observational phase (LogPad):
  - QIDS-SR_{16}
  - GAD-7
  - MOS Sleep-R
  - WHODAS 2.0
  - EQ-5D-5L
  - PSS
  - PAQ
- Relapse Verification visit (SitePad):
  - QIDS-SR_{16}
  - RLCST
  - FSR-SA (subjects who relapse but do not require a Relapse Verification visit (ie, they meet relapse criteria other than that based on the MADRS total score) will complete the FSR-SA at their next scheduled visit)
- Relapse Verification visit (LogPad):
  - GAD-7
  - PSS
  - PAQ

Healthcare resource utilization data will be collected for this study. Refer to Section 9.5 for details.

The total blood volume for the study will be approximately 250 mL for a subject in the study for 1 year (70 mL for genetics, epigenetics, and transcriptomics, and 180 mL for biomarkers (see Table 3).

Table 3. Volume of Blood to Be Collected From Each Subject

Type of Sample	Volume per Sample (mL)	No. of Samples per Subject	Total Volume of Blood (mL)[a]
Biomarkers[b,c]	10	18	180
Genetic (DNA) sample [b.d]	10	1	10
Epigenetic (DNA) sample [b.d]	10	3	30
Transcriptomics (RNA) sample[b]	10	3	30
Approximate total			250

Note: Estimates based on study participation of 1 year.

[a] Calculated as number of samples multiplied by amount of blood per sample.

[b] Repeat or unscheduled samples may be taken for safety reasons or technical issues with the samples.

[c] For biomarker analyses, two 10-mL blood samples will be taken (for serum and plasma preparation) at each scheduled time point as shown in the Time and Events Schedule.

[d] For genetic, epigenetic, and transcriptomics research, a 10-mL blood sample for each will be collected at the time points as shown in the Time and Events Schedule.

9.1.2. Screening Phase

Prior to conducting any study procedure, the investigator (or designated study personnel) will review and explain the written informed consent form (ICF) to each subject. After signing the ICF, the subject will be screened within 2 weeks before Day 1 to determine eligibility for study participation. If feasible, the screening and baseline visits can occur on the same day. Duplicate assessments will not be required if the screening and baseline visits occur on the same day.

All adverse events and special reporting situations, whether serious or non-serious, will be reported from the time a signed and dated ICF is obtained until completion of the subject's last study-related procedure (which may include contact for follow-up of safety).

Screening evaluations will be performed as per the Time and Events Schedule.

9.1.2.1. Early Trauma Inventory Self-Report – Short Form

Information about early childhood trauma may provide additional important information about factors relating to the development of a range of adverse mental health outcomes, including MDD. The Early Trauma Inventory Self-Report – Short Form (ETISR-SF) is a 27-item subject-reported assessment of childhood trauma (before 18 years of age), which is derived from the original 62-item ETI-SR questionnaire. The instrument is simple to administer and has been shown to have acceptable validity and internal consistency.[4,5] The questionnaire includes questions (yes, no) divided into 4 domains (general traumas, physical punishment, emotional abuse, and sexual events), and is scored by counting the number of positive responses. An example of the ETISR-SF is provided in Attachment 2.

9.1.2.2. Cumulative Illness Rating Scale

The Cumulative Illness Rating Scale (CIRS) is a clinician-administered assessment for the presence of chronic medical conditions. The scale is adapted from one developed and used in the geriatric population,[37] and it has been used in other large studies in non-geriatric MDD subjects, such as the STAR*D study.[52] For each category, 0=no problem; 1=mild problem; 2=problem of moderate severity requiring active therapy; 3=severe or constant disability; 4=extremely severe or urgent clinical problem.[37] An example of the CIRS is provided in Attachment 3.

9.1.2.3. MINI International Neuropsychiatric Interview

Subjects will undergo a brief diagnostic interview (MINI International Neuropsychiatric Interview; MINI - DSM-5) to confirm that the diagnosis of MDD was present prior to their antidepressant response, and to determine if there are other psychiatric conditions present.[34]

9.1.2.4. Massachusetts General Hospital Antidepressant Treatment Response Questionnaire

The MGH-ATRQ evaluates the adequacy of duration and dosage of all antidepressant medications used for the current MDE. In addition, the MGH-ATRQ assesses the degree of improvement (in the most efficacious or in all trials during the current MDE, depending on the version of the instrument), on a scale from 0% (not improved at all) to 100% (completely improved). The MGH-ATRQ will be completed by the clinician, in collaboration with the subject. An example of the MGH-ATRQ is provided in Attachment 4.

9.1.3. Observational Phase

At baseline, eligible subjects will complete assessments including clinician- and subject-administered assessments of mood, as well as several other assessments as outlined in the Time and Events Schedule. All subjects will receive a study-specific smartphone (LogPad®), which they will use to complete assessments at and between study visits. Subjects will continue in the study until relapse has been observed in 150 subjects. Subjects will participate in frequent assessments using self-report measures of symptoms, antidepressant medication adherence, functioning, health-related quality of life, and sleep. Clinician ratings of symptom severity will be collected at routine study visits (8-week intervals); medication adherence (prescription history) will also be assessed by the investigator at each study visit. Subjects will provide speech and voice samples for evaluation. Subjects will wear an Actiwatch Spectrum wrist actigraphy device, which will provide data about activity levels and sleep.

Relapse will be defined using multiple criteria, including clinician rating (MADRS), hospitalization, and suicidal ideation and behavior (see Section 9.2.2). If the MADRS criterion is met at a study visit (scheduled or unscheduled), an additional visit (ie, the Relapse Verification visit) will be scheduled within 1 to 2 weeks to verify the relapse. The relapse criteria used are consistent with criteria used in clinical studies in this patient population.

Subjects who relapse will continue in the observational phase until relapse has been observed in 150 subjects. Appropriate adjustment in treatment will be made by the investigator as needed and if willing, subjects will continue to participate in frequent assessments using self-report measures

of symptoms, antidepressant medication adherence, functioning, health-related quality of life, and sleep as well as attending routine study visits at 8-week intervals.

Self-report and speech/voice behavioral data will be transmitted to a study data file via a study-specific smartphone (LogPad®). Actigraphy data will be downloaded and transmitted to the study data file at study visits. As the study is observational in nature, these data will not be made available to the study site staff during the study visits.

End-of-Study/Early Withdrawal

The study will end once relapse has been observed in 150 subjects. At that time, all subjects will conclude study participation with an end-of-study visit conducted at the next scheduled study visit (ie, 8-week interval), and will then be referred for further follow-up with their treating physician. Subjects withdrawing early from the study will be asked to return to the clinic to complete an end-of-study/early withdrawal visit unless they withdraw consent to participate in the study.

Subjects who relapse during the observational phase of the study will not be considered as early withdrawals as long as they remain in the study until relapse has been observed in 150 subjects.

9.2. Clinical Assessments

9.2.1. Evaluations

Every effort should be made to ensure that all clinician-administered assessments are completed by the same individual who made the initial baseline determinations.

The measures selected for this study represent symptom and behavior domains identified as potential predictors of relapse. Given the exploratory nature of this study, a comprehensive set of assessments is included, with the objective of developing the most robust predictive models of impending relapse.

9.2.1.1. Montgomery Asberg Depression Rating Scale

The primary measure to evaluate depressive symptoms, including whether relapse has occurred, will be the MADRS total score.

The MADRS is a clinician-rated scale, designed to measure depression severity and detects changes due to antidepressant treatment.[39] The test consists of 10 items, each of which is scored from 0 (item not present or normal) to 6 (severe or continuous presence of the symptoms), for a total possible score of 60. Higher scores represent a more severe condition. The MADRS evaluates apparent sadness, reported sadness, inner tension, sleep, appetite, concentration, lassitude, interest level, pessimistic thoughts, and suicidal thoughts. The test exhibits high inter-rater reliability.

The structured interview guide for the MADRS (SIGMA) will be used for each administration.[71] Using structured interview guides has previously been shown to increase the reliability of given

scales. The typical recall period for the MADRS is 7 days. An example of the MADRS is provided in Attachment 5.

9.2.1.2. Clinical Global Impression - Severity

The CGI-S is a physician-rated scale that is designed to rate the severity of the subject's illness at the time of assessment, relative to the clinician's past experience with subjects who have the same diagnosis and improvement with treatment.[18] Considering total clinical experience, a subject is assessed on the severity of mental illness at the time of rating according to: 0=not assessed; 1=normal (not at all ill); 2=borderline mentally ill; 3=mildly ill; 4=moderately ill; 5=markedly ill; 6=severely ill; 7=among the most extremely ill patients. An example of the CGI-S is provided in Attachment 6.

9.2.1.3. Snaith Hamilton Pleasure Scale

The Snaith-Hamilton Pleasure Scale (SHAPS) is a brief 14-item patient-reported assessment scale for estimation of the degree to which a person is able to experience pleasure or the anticipation of a pleasurable experience. The items relate to experiences likely to be encountered by most people. The SHAPS covers 4 domains of hedonic experience: interest/pastimes, social interaction, sensory experience, and food/drink. Each of these domains is based on only a few items, and it is not currently recommended that subscores be used.[57] An example of the SHAPS is provided in Attachment 7.

9.2.1.4. Quick Inventory of Depressive Symptomatology-Self Report-16-Item

The QIDS-SR_{16} is a patient-reported measure designed to assess the severity of depressive symptoms.[51,62] The QIDS-SR_{16} assesses all the criterion symptom domains designated by the DSM-5 to diagnose a major depressive episode. This assessment can be used to screen for depression, although it has been used predominantly as a measure of symptom severity. Subjects provide responses to each item of this instrument with a 4-point Likert scale, with scores ranging from 0 to 3 for each item. The 7-day period prior to assessment is the usual recall period for assessing symptom severity. The scoring system of the QIDS converts responses to the 16 separate items into the 9 DSM-5 symptom criterion domains. The 9 domains comprise: 1) sad mood; 2) concentration; 3) self-criticism; 4) suicidal ideation; 5) interest; 6) energy/fatigue; 7) sleep disturbance (initial, middle, and late insomnia or hypersomnia); 8) decrease or increase in appetite or weight; and 9) psychomotor agitation or retardation. The total score is obtained by adding the scores for each of the 9 symptom domains of the DSM-5 MDD criteria[51]: 4 items are used to rate sleep disturbance (early, middle, and late insomnia plus hypersomnia); 2 items are used to rate psychomotor agitation and retardation; 4 items are used to rate appetite (increase or decrease and weight increase or decrease). One item is used to rate the remaining 6 domains (sad mood, interest, energy/fatigue, self-criticism, concentration, and suicidal ideation). For symptom domains that require more than 1 item, the highest score of the item relevant for each domain is taken. For example, if early insomnia is 0, middle insomnia is 1, late insomnia is 3, and hypersomnia is 0, the sleep disturbance domain is rated 3. The total score ranges from 0 to 27. Using a scale of severity of depression of none, mild, moderate, severe, and very severe, corresponding QIDS-SR_{16} total scores are none, 1 to 5; mild, 6 to 10; moderate, 11 to 15; severe, 16 to 20; and very severe, 21 to 27.

The $QIDS\text{-}SR_{16}$ is sensitive to change with medications, psychotherapy, or somatic treatments. The psychometric properties of the $QIDS\text{-}SR_{16}$ have been established in various study samples, and are outlined on the developer's website.[65] An example of the $QIDS\text{-}SR_{16}$ is provided in Attachment 8.

9.2.1.5. Seven-Item Generalized Anxiety Disorder Scale

The GAD-7 is a validated, brief 7-item self-report assessment of anxiety. Each item is scored on a 4-point scale (0 to 3), with a total score range of 0 to 21.[59] The standard recall period used is 2 weeks, but in this study a 7-day recall period will be used. An example of the GAD-7 is provided in Attachment 9.

9.2.1.6. Perceived Stress Scale

The PSS is a 10-item, self-reported, unidimensional instrument developed to measure perceived stress in response to situations in a person's life.[7] Respondents report the prevalence of an item within the last month on a 5-point scale, ranging from "never" to "very often." The PSS is a widely used scale for measuring the perception of stress. It is a measure of the degree to which situations in one's life are appraised as stressful. Items are designed to tap how unpredictable, uncontrollable, and overloaded respondents find their lives. The scale also includes a number of direct queries about current levels of experienced stress. Moreover, the questions are of a general nature, and hence are relatively free of content specific to any subpopulation group. The questions in the PSS ask about feelings and thoughts during the last month. In each case, respondents are asked how often they felt a certain way. An example of the PSS is provided in Attachment 10.

9.2.1.7. Medical Outcomes Study Sleep Scale, Revised, 12-Items

The MOS Sleep-R is a subject-completed scale containing 12 items that addresses various dimensions of sleep. The instrument yields 6 subscales: sleep disturbance, snoring, shortness of breath or headache, sleep adequacy, sleep somnolence, and sleep quantity. Ten of the items are answered on 5-point Likert scales, where 1=all of the time and 5=none of the time. One item, on sleep latency, is answered on a 5 point Likert scale from 1=0-15 minutes to 5=more than 60 minutes. The final item, on the duration of sleep, allows the subject to write in the number of hours slept per night. The acute version to be used in this study has a recall period of the past week. Quantity of sleep is scored as the average number of hours slept per night. Other subscale scores are converted to a T-score with a mean of 50, SD of 10, and a range of 0 to 100, where higher scores indicate fewer sleep-related problems. The instrument has good data supporting its psychometric properties and development history.[49] An example of the MOS Sleep-R is provided in Attachment 11.

9.2.1.8. EuroQol Group; 5 Dimension; 5 Level

The EQ-5D-5L is a standardized 2-part instrument for use as a measure of health outcome, primarily designed for self-completion by respondents.[6,10,11,68] It essentially consists of the EQ-5D descriptive system and the EQ visual analogue scale (EQVAS). The EQ-5D-5L descriptive system comprises the following 5 dimensions: mobility, self-care, usual activities,

pain/discomfort, and anxiety/depression. Each dimension has 5 levels: no problems, slight problems, moderate problems, severe problems, and extreme problems. The descriptive system can be represented as a health state. The EQVAS self-rating records the respondent's own assessment of their health status. Subjects select an answer for each of the 5 dimensions considering the response that best matches their health "today." An example of the EQ-5D-5L is provided in Attachment 12.

9.2.1.9. World Health Organization Disability Assessment Schedule 2.0

The World Health Organization Disability Assessment Schedule (WHODAS 2.0) is a generic instrument developed by the WHO to provide a standardized method for measuring health and disability across cultures.[70] It was developed from a comprehensive set of International Classification of Functioning, Disability, and Health (ICF) items. The WHODAS 2.0 is a 12-item self-administered scale measuring the amount of difficulty a subject has completing specific activities. Each item is rated on a 5-item Likert scale, with responses ranging from 1=no difficulty to 5=extreme difficulty and a recall period of the past 30 days. The scale assesses the following 6 domains: cognition, mobility, self-care, getting along (interacting with other people), life activities, and participation. The 12-item WHODAS 2.0 is reported to be very good at assessing overall disability, with results supporting the appropriateness of the weights assigned to response option categories and showing an absence of sex differences in item functioning.[30] An example of the WHODAS 2.0 is provided in Attachment 13.

9.2.1.10. Recent Life Change Stress Test

The Recent Life Changes Stress Test (RLCST) is a subject-completed scale that measures the number of life change events, such as marriage or death of a family member, the subject has experienced or expects to experience in the near future.[36] Each item has been constructed to contain life events whose advent is either indicative of or requires a significant change in the ongoing life pattern of the individual. Events are scored based on the degree of meaningfulness to subjects, as identified during questionnaire development.[19] A higher score indicates a greater amount of meaningful life events. The baseline assessment for the RLCST will have a 1-year recall period; the since last visit version will have a recall period of the last 8 weeks. An example of the RLCST (Baseline) is provided in Attachment 14; an example of the RLCST (Since Last Visit) is provided in Attachment 15.

9.2.1.11. The Pain Frequency, Intensity, and Burden Scale

The Pain Frequency, Intensity, and Burden Scale (P-FIBS) is a 4-item brief self-reported measure developed to assess pain in research settings or as a screening tool in clinical settings. Each of the 4 items rated on a 9-point Likert scale (0 to 8), with lower scores indicating less pain or burden during the past week. The score is computed by summing responses to each item. Frequency and intensity of pain are measured with 1 item each. Burden of pain is assessed with 2 items, 1 assessing the extent to which pain interferes with daily life and 1 assessing the use of medications or other treatment to manage pain. The 4 items on the P-FIBS demonstrate high item-total correlations (range 0.70-0.85) with a high Cronbach's alpha (0.90). The P-FIBS demonstrated a strong negative correlation with the bodily pain sub-score of the Short Form Health Survey ($r=-0.76$, $p<0.0001$).[9] An example of the P-FIBS is provided in Attachment 16.

9.2.1.12. Healthcare Resource Use Questionnaire

The Healthcare Resource Use Questionnaire (HRUQ) is a clinician-administered assessment of medical resource utilization, developed by Janssen, of the utilization of health care services, including hospitalizations, emergency room visits, day hospital attendance, outpatient visits, and work productivity. There are 2 versions of the assessment, a baseline version (last 6 months), and a version that captures data since the last visit. At baseline, subjects will be asked to use a recall period of the prior 6 months; at subsequent study visits, subjects will be asked to recall the period since the last visit.

9.2.1.13. Depression Implicit Association Test

The Harvard/Project Implicit Mental Health (PIMH) depression-specific Implicit Association Test (IAT) will be used in this study. Association tests are designed to assess automatic cognitive processes[15]; subjects are asked to categorize a concept (eg, “me” vs “not me”) with an attribute (eg, “logical”) by responding to stimulus words (ie, stimulus words including synonyms as well as antonyms of the attribute being tested). Reaction time is measured; faster responding is interpreted to represent stronger automatic associations between concept and attribute. Versions of the test have been developed to evaluate positive vs negative implicit associations about self. Depressed subjects have been shown to exhibit a cognitive bias towards negative self-evaluation.[56] In a study comparing IAT performance of subjects who were formerly depressed (ie, previously met criteria for MDD, but did not meet MDD criteria on study enrollment) vs current MDD subjects and healthy controls, Gemar et al.[14] reported that “automatic evaluative biases” (ie, biases towards negative self-categorization) in subjects with a history of MDD were predictive of risk for recurrence of future MDEs.”

The PIMH Depression IAT contains 6 blocks of 20 words/comparisons in each block. It is computer-administered, and requires approximately 10 minutes to complete. It has been validated in subjects with MDD, including evaluation of the effects of a negative mood induction procedure on automatic associations between the self and mood state in subjects with a history of MDD.[35]

9.2.1.14. Assessment of Speech and Voice

Speech and voice characteristics will be assessed using speech diary recordings via a study-specific smartphone (LogPad®). Subjects will use a software application on the smartphone to record speech at 2-week intervals. The application will prompt each subject to describe:

- His or her physical condition during the past 2 weeks
- His or her mental condition during the past 2 weeks
- How his or her physical and mental feelings have affected his or her ability to function during the past 2 weeks
- The event during the past 2 weeks that made him or her the happiest

Speech samples will be downloaded to a secure server and scored for speech rate and vocal parameters via automated scoring software (MIT Lincoln Laboratory).[72] Speech rate and vocal acoustic features have been shown to predict depression severity and treatment response.[42,72]

9.2.1.15. Actigraphy

Actigraphy has been used in multiple studies in MDD to measure sleep, including sleep efficiency and sleep latency, and is sensitive to the differential effects of antidepressant treatment regimens on sleep symptoms.[23] Activity and sleep parameters will be measured using an Actiwatch® Spectrum wrist actigraphy device. Subjects will wear the device continuously. Data will be downloaded and transmitted electronically to a study datafile during study visits. Actigraphy measures will include:

- Sleep duration
  - Total sleep time per night (hours)
- Sleep pattern
  - Time of sleep onset (time of day)
  - Time of sleep end (time of day)
- Sleep quality
  - Mean activity during rest period (counts/minute)
  - Sleep fragmentation (%)
  - Sleep efficiency (%)
  - Actigraphic estimate of wake after sleep onset (minutes)
- Daytime activity
  - Mean daytime activity (counts/minute)
  - Peak daytime activity (counts/minute)

9.2.1.16. First Symptom of Relapse – Self-Assessment

In order to obtain additional information from the subject's perspective about possible predictors of relapse that is not captured by other measures included in the study, the subject will complete the First Symptom of Relapse – Self-Assessment (FSR-SA), a single-item assessment developed for this study, after the subject has been confirmed to have experienced a relapse (see Criteria in Section 9.2.2). The subject will be asked an open-ended question to report the first sign, symptom, or indication that he/she noticed that made the subject aware of this worsening of depression. An example of the FSR-SA is provided in Attachment 17.

9.2.2. Endpoints

The objective of this exploratory, prospective cohort trial is to identify if there are self-reported or objective measures that can predict near-term relapse (approximately 1 month, or at another identified time point, prior to meeting the criteria for clinical relapse). Subjects will be followed

in the study until relapse has been observed in 150 subjects. Subjects may participate in the study for a variable duration until the target number of relapses has occurred. Once relapse has been observed in 150 subjects, all subjects will conclude study participation with an end-of-study visit conducted at the next scheduled study visit (ie, 8-week interval), and will then be referred for further follow-up with their treating physician. Relapse will be analyzed as the dependent variable in statistical analyses (ie, modeling re: predictors of relapse).

Subjects who relapse will continue in the study, ie, the intent is to continue to follow all relapsed and non-relapsed subjects. Relapse will only be counted once for any given subject.

An Adjudication Committee will be commissioned for this study, to adjudicate any cases of relapse where it is unclear if relapse occurred or where further discussion is needed. Refer to Section 11.7, Adjudication Committee, for details.

Criteria

Relapse will be defined as any of the following:

- MADRS total score ≥22 for at least 2 consecutive weeks. If this criterion is met at a study visit (scheduled or unscheduled), an additional visit (ie, the Relapse Verification visit) will be scheduled within 1 to 2 weeks to verify the relapse.
- Hospitalization for worsening of depression
- Suicidal ideation with intent, or suicidal behavior
- Other (investigator will be asked to describe)

9.3. Fluid Biomarkers

Blood samples will be collected to study the potential association of biomarkers with relapse in MDD. The Human Discovery MAP™ (Multi-Analyte Panel), a panel of approximately 250 proteins involved in key biologically relevant pathways associated with major depression, including neurotrophic and inflammatory factors, will be used.

In addition, analysis of specific biomarker analytes may include (but not limited to) growth factors such as brain-derived neurotrophic factor (BDNF), cytokines and markers of the inflammatory pathway, and endocrine and metabolic markers. Blood samples (for serum and plasma preparation) will be collected at the frequency shown in the Time and Events Schedule.

9.4. Genetic and Epigenetic (DNA) and Transcriptomic (RNA) Evaluations

The DNA (genetics and epigenetics) and RNA (transcriptomics) component of the study is mandatory. Blood samples for DNA (genetic and epigenetic research) and RNA (transcriptomics) will be used for exploratory research related to MDD and relapse. They may also be used to develop tests/assays related to MDD and relapse. Genetic and epigenetic (DNA) and transcriptomic (RNA) research may consist of the analysis of one or more candidate genes/transcripts or of the analysis of genetic markers throughout the genome (as appropriate) in

relation to MDD clinical endpoints, including relapse. Blood samples for DNA and RNA will be collected at the frequency shown in the Time and Events Schedule.

9.5. Healthcare Resource Utilization

Healthcare resource utilization data, associated with medical encounters, as well as work productivity data, will be collected electronically, using the HRUQ (see Section 9.2.1.12), by the investigator and study-site personnel for all subjects throughout the study. The data collected may be used to conduct exploratory economic analyses and will include:

- Number and duration of medical care encounters, including hospitalizations, emergency room visits, day hospital attendance, and non-study-related clinic visits (MDD- and non-MDD-related)
- For hospitalization, data will include total days length of stay, including duration by wards, eg, intensive care unit
- Assessment of occupational status as well as illness-related loss of working days/loss of days at an educational institution

9.6. Safety Evaluations

Safety evaluations will include:

- Vital signs and weight
- Adverse event monitoring
- Concomitant therapy
- Suicide assessment, as measured by the CHRT
- Urine pregnancy testing for female subjects of child-bearing potential
- Urine drug screen

Any clinically relevant changes occurring during the study must be recorded on the Adverse Event section of the CRF.

Any clinically significant abnormalities persisting at the end of the study/early withdrawal will be followed by the investigator until resolution or until a clinically stable endpoint is reached.

The study will include the following evaluations of safety and tolerability according to the time points provided in the Time and Events Schedule:

Adverse Events

Adverse events will be reported by the subject (or, when appropriate, by a caregiver, surrogate, or the subject's legally acceptable representative) for the duration of the study. Adverse events will be followed by the investigator as specified in Section 12.4.1. Reporting Procedures for Adverse Events and Pregnancies or Pregnancies in Partners for Janssen Products Adverse Event Reporting.

Vital Signs (Pulse/Heart Rate, Blood Pressure)

Vital signs, including body weight, will be measured at each study visit.

Blood pressure and pulse/heart rate measurements will be assessed in a supine position with a completely automated device. Manual techniques will be used only if an automated device is not available.

Blood pressure and pulse/heart rate measurements should be preceded by at least 5 minutes of rest in a quiet setting without distractions (eg, television, cell phones).

Physical Examination

A physical examination will be performed at screening and at the end-of-study/early withdrawal visit. Height will be measured at screening only.

Suicide Assessment

Concise Health Risk Tracking Scale

The CHRT scale was developed by Trivedi et al.[63] as a tool to screen for suicidal ideation and related symptoms, as well as allowing for the prospective assessment of subjects for the occurrence of treatment-emergent suicidality. The CHRT has 2 sections:

- A 16-item subject self-reported screen for suicidal ideation and associated symptoms (Items 1 to 16)
- A clinician-rated behavioral module, Suicidal Behavioral Evaluation, which covers all Columbia Classification Algorithm of Suicide Assessment (C-CASA) domains[47] (Items 1 to 9) and involves actively querying subjects about the occurrence of suicidal thinking

At the screening visit, the CHRT baseline visit assessment will be used, which has a 7-day recall period. For all subsequent time points, the CHRT follow-up assessment, which has a recall period of since last visit, will be used. An example of the CHRT (Baseline) is provided in Attachment 18; an example of the CHRT (Since Last Visit) is provided in Attachment 19.

Urine Pregnancy Testing

Urine pregnancy test will be performed for all female subjects of childbearing potential.

Urine Drug Screen

The urine drug screen will include testing for barbiturates, methadone, opiates, cocaine, phencyclidine, amphetamine/methamphetamine, and cannabinoids.

9.7. Sample Collection and Handling

Venous blood samples for measuring biomarkers will be collected in all subjects. The actual dates and times of sample collection must be recorded in the CRF or laboratory requisition form.

For biomarker analyses, two 10-mL blood samples will be taken (for serum and plasma preparation) at each collection visit.

For genetic, epigenetic, and transcriptomics research, a 10-mL blood sample for each will be collected. Refer to the Time and Events Schedule for the timing and frequency of all sample collections.

Instructions for the collection, handling, storage, and shipment of samples are found in the laboratory manual that will be provided.

Subjects who withdraw from the study should have a final blood sample collected at the end-of-study/early withdrawal visit.

10. SUBJECT COMPLETION/WITHDRAWAL

10.1. Completion

A subject will be considered to have completed the study if he or she has completed assessments at the end of the observational phase (once relapse has been observed in 150 subjects). The study will end once relapse has been observed in 150 subjects. At that time, all ongoing subjects will conclude study participation with an end-of-study visit conducted at the next scheduled study visit (ie, 8-week interval), and will be referred for further follow-up with their treating physician. Subjects who prematurely discontinue from the study for any reason before completion of the observational phase will not be considered to have completed the study.

10.2. Withdrawal From the Study

A subject will be withdrawn from the study for any of the following reasons:

- Lost to follow-up
- Withdrawal of consent
- Death
- The investigator or sponsor believes it is in the best interest of the subject to discontinue from the study

If a subject is lost to follow-up, every reasonable effort must be made by the study site personnel to contact the subject and determine the reason for discontinuation/withdrawal. The measures taken to follow-up must be documented.

When a subject withdraws before completing the study, the reason for withdrawal is to be documented in the CRF and in the source document. If a subject withdraws from the study before the end of the observational phase, end-of-study assessments should be obtained.

Subjects who relapse will continue in the study until relapse has been observed in 150 subjects, ie, the intent is to continue to follow all relapsed and non-relapsed subjects. Relapse will only be counted once for any given subject.

Withdrawal From the Use of Samples in Future Research

The subject may withdraw consent for use of samples for research (refer to Section 14.2.5, Long-Term Retention of Samples for Additional Future Research). In such a case, samples will be

destroyed after they are no longer needed for the clinical study. Details of the sample retention for research are presented in the main ICF.

11. STATISTICAL METHODS

Statistical analysis will be done by the sponsor or under the authority of the sponsor. A general description of the statistical methods to be used to analyze the efficacy and safety data is outlined below. Specific details will be provided in the Statistical Analysis Plan.

11.1. Subject Information

For all enrolled subjects, descriptive statistics will be provided.

11.2. Sample Size Determination

Assumptions about observed relapses among subjects entering into the study were modeled from the 12-month naturalistic follow-up study of the STAR*D study (see description of the STAR*D study in Section 3.2).[52] In the STAR*D follow-up study, approximately 30% of subjects who entered the 12-month follow-up study were observed to relapse within 6 months. Approximately 46% of subjects entering the follow-up study were observed to relapse within 12 months. Based on these data, for this study, a sample size of approximately 330 subjects is projected to result in 150 subjects who relapse.

A sample size of 330 subjects produces a 2-sided 95% confidence interval with a width equal to 0.108 when the relapse rate is 0.46. For a relapse rate of 0.3, the width of the confidence interval decreases to 0.098 (exact confidence limit according to Wilson) (see Figure 2). Sample sizes from 300 to 500 subjects, and the confidence intervals for relapse rates of 30% to 50%, are provided in Table 4.

A total of 150 subjects with observed relapse is a sufficient number of relapses to enable multivariate predictive models, and is based on the rate of relapse observed in the follow-up phase in the STAR*D study.[52] Individual items/measures from self-report and clinician ratings, and summary data from actigraphy assessments (eg, hours of sleep per night), will be used in the development of predictive models. Given the heterogeneity of MDD, predictors of near-term relapse may differ across subgroups of patients; therefore, multiple predictive models of near-term relapse may be calculated across subsets of MDD subjects.

Figure 2. Width of the 95% Confidence Interval for Different Expected Proportions and Total N

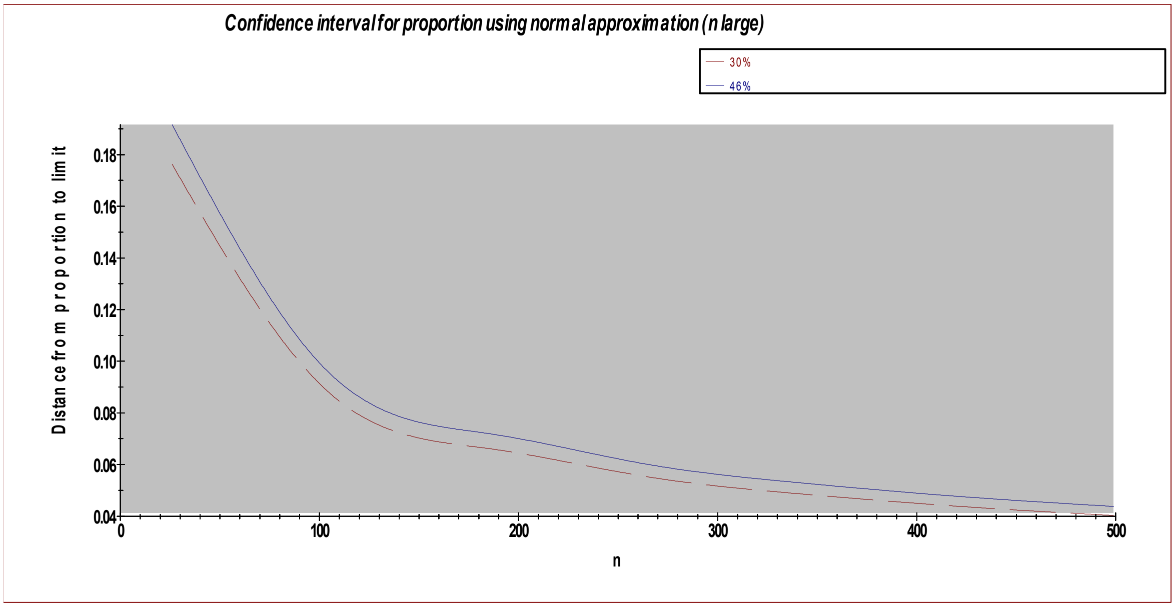

Table 4. Precision Level and Confidence Intervals for Sample Sizes of 300 to 500 Subjects and Relapse Rates of 30% to 50%

		Relapse Rate		
Sample Size	Expected Proportion	30%	40%	50%
300	Precision (%)	5.2	5.5	5.7
	CI (%)	(24.8, 35.2)	(34.2, 45.5)	(44.3, 55.7)
350	Precision (%)	4.8	5.1	5.2
	CI (%)	(25.2, 34.8)	(34.9, 45.1)	(44.8, 55.2)
400	Precision (%)	4.5	4.8	4.9
	CI (%)	(25.5, 34.5)	(35.2, 44.8)	(45.1, 54.9)
450	Precision (%)	4.2	4.5	4.6
	CI (%)	(25.8, 34.2)	(35.5, 44.5)	(45.4, 54.6)
500	Precision (%)	4.0	4.3	4.4
	CI (%)	(26.0, 34.0)	(35.7, 44.3)	(45.6, 54.4)

11.3. Analysis

The relapse criteria are defined in Section 9.2.2.

Because of the exploratory nature of the study, no hypotheses will be tested. Predictive models of relapse will be developed, especially modeling of relapse using current/most recent assessment scores as covariates (and/or change scores calculated using current/most recent assessments). While the aim is for prediction of relapse within 1 month or at another identified time point before meeting the criteria for relapse, the time frame for final models will be based on the actual data, and model development will consider various time frames in order to identify the best predictors.

Several exploratory methods will be used to quantify the association between potential risk factors and near-term relapse in subjects with depression.

Descriptive statistics for continuous factors will be presented for subjects by the outcome variable of relapse. Frequency tables of categorical factors will be presented, along with chi-square analyses.

A prediction rule will be developed based on the linear predictor in a logistics regression model in which the outcome variable is near-term relapse.

A non-parametric technique of recursive partitioning will be performed that results in a tree-structured association of covariates with an outcome. The advantage of this technique for the examination of association is the ability to examine conditional interactions in the data, and the resultant decision tree. Missing data are less problematic with this technique than with other multivariate methods because surrogate variables are identified.

Model development will utilize repetitive sampling,[22] by which predictive models can be cross-validated within repeat subsamples.

11.4. Analyses of Fluid Biomarkers and Genetic, Epigenetic, and Transcriptomics Data

Changes in Human Discover MAP™ (Multi-Analyte Panel) biomarkers over time will be summarized for relapse (prior to and postrelapse) and nonrelapse groups. In addition, changes in specific biomarker analytes, which may include (but not limited to) growth factors such as brain-derived neurotrophic factor (BDNF), cytokines and markers of the inflammatory pathway, and endocrine and metabolic markers, will be summarized for the relapse (prior to and postrelapse) and nonrelapse groups.

Genetic, epigenetic, and transcriptomics data will be compared between the relapse (prior to and postrelapse) and nonrelapse groups.

Results will be presented in a separate report.

11.5. Healthcare Resource Utilization Analyses

Healthcare resource utilization and work productivity data will be descriptively summarized.

11.6. Safety Analyses

Adverse Events

The verbatim terms used in the CRF by investigators to identify adverse events will be coded using the Medical Dictionary for Regulatory Activities (MedDRA). All reported adverse events with onset during the observational phase will be included in the analysis. For each adverse event, the percentage of subjects who experience at least 1 occurrence of the given event will be summarized.

Summaries, listings, datasets, or subject narratives may be provided, as appropriate, for those subjects who die, who discontinue the study due to an adverse event, or who experience a severe or a serious adverse event.

Vital Signs

Descriptive statistics of pulse/heart rate, blood pressure (systolic and diastolic), and weight values and changes from baseline will be summarized at each scheduled time point. The percentage of subjects with values beyond clinically important limits will be summarized.

Physical Examination

Descriptive statistics of changes from baseline to end-of-study/early withdrawal will be summarized for physical examination. Descriptive statistics will be provided for height at screening.

Suicidal Ideation and Behavior

Suicide-related thoughts and behaviors, based on data collected from the CHRT, will be summarized in incidence and shift tables. Separate endpoints for suicidal ideation and suicidal behavior will be defined and summarized descriptively.

11.7. Adjudication Committee

An Adjudication Committee will be established to monitor data on an ongoing basis, to adjudicate any cases of relapse where it is unclear if relapse occurred or where further discussion is needed. The committee will meet periodically to review interim data. The details will be provided in a separate Adjudication Committee charter.

The Adjudication Committee will consist of at least one medical expert in the relevant therapeutic area and at least one statistician. The Adjudication Committee responsibilities, authorities, and procedures will be documented in its charter.

12. MANAGEMENT AND REPORTING OF ADVERSE EVENTS/ADVERSE REACTIONS

Timely, accurate, and complete reporting and analysis of safety information from clinical studies are crucial for the protection of subjects, investigators, and the sponsor, and are mandated by regulatory agencies worldwide. All studies conducted by the sponsor or its affiliates will be conducted in accordance with established procedures and regulatory requirements worldwide to ensure appropriate reporting of safety information.

12.1. Definitions

12.1.1. Adverse Event Definitions and Classifications

Adverse Event

Although subjects will not receive any treatment as part of the study, adverse events will be recorded throughout the study. An adverse event is any untoward medical occurrence in a subject or clinical study subject. An adverse event does not necessarily have a causal relationship with any treatment taken. An adverse event can therefore be any unfavorable and unintended sign (including an abnormal finding or lack of expected pharmacological action), symptom, or disease temporally associated with the use of a medicinal (investigational or non-investigational)

product, whether or not related to that medicinal (investigational or non-investigational) product. (Definition per International Conference on Harmonisation [ICH])

This includes any occurrence that is new in onset or aggravated in severity from the baseline condition, or abnormal results of diagnostic procedures that are conducted per clinical practice.

Adverse Drug Reaction

An adverse drug reaction (ADR) is defined as a response to a medicinal (investigational or non-investigational) product that is noxious and unintended. The phrase "response to a medicinal product" means that a causal relationship between a medicinal product and an adverse event is at least a possibility; ie, the relationship cannot be ruled out.

An ADR, in contrast to an adverse event, is characterized by the fact that a causal relationship between the medicinal product and the occurrence is suspected. All adverse events judged by either the reporting investigator or the sponsor as having a reasonable causal relationship to a medicinal product qualify as ADRs.

Serious Adverse Event or Serious Adverse Drug Reaction

A serious adverse event (or serious ADR) based on ICH and EU Guidelines on Pharmacovigilance for Medicinal Products for Human Use is any untoward medical occurrence (or a "response to a medicinal product" as defined above) that:

- Results in death
- Is life-threatening
  (The subject was at risk of death at the time of the event. It does not refer to an event that hypothetically might have caused death if it were more severe.)
- Requires inpatient hospitalization or prolongation of existing hospitalization
- Results in persistent or significant disability/incapacity
- Is a congenital anomaly/birth defect
- Is a suspected transmission of any infectious agent via a medicinal product
- Is medically important*

*Medical and scientific judgment should be exercised in deciding whether other situations should be considered serious, such as important medical events that may not be immediately life-threatening or result in death or hospitalization but may jeopardize the subject or may require intervention to prevent one of the other outcomes listed above.

For reports of hospitalization, it is the sign, symptom, or diagnosis that led to hospitalization that is the serious event for which details must be provided. Any event requiring hospitalization (or prolongation of hospitalization) that occurs during the course of a subject's participation in a study must be reported as a serious adverse event, except hospitalizations for the following:

- Hospitalizations not intended to treat an acute illness or adverse event (eg, social reasons such as pending placement in long-term care facility)
- Surgery or procedure planned before entry into the study. Note: Hospitalizations that were planned before the start of data collection, and where the underlying condition for which the hospitalization was planned has not worsened, will not be considered serious adverse events. Any adverse event that results in a prolongation of the originally planned hospitalization is to be reported as a new serious adverse event.

The cause of death of a subject in a study, whether or not the event is expected or associated with any medicinal product (investigational or non-investigational), is considered a serious adverse event.

Unlisted (Unexpected) Adverse Event/Reference Safety Information

An adverse event is considered unlisted if the nature or severity is not consistent with the applicable product reference safety information. For a medicinal product(s) with a marketing authorization, the expectedness of an adverse event will be determined by whether or not it is listed in the applicable product information.

12.1.2. Attribution and Severity Definitions

An adverse event is considered not associated with the use of the treatment if the attribution is not related or doubtful according to the definitions listed below:

Not Related

An adverse event that is not related to the use of the treatment.

Doubtful

An adverse event for which an alternative explanation is more likely, eg, concomitant drug(s), concomitant disease(s), and/or the relationship in time suggests that a causal relationship is unlikely.

An adverse event is considered associated with the use of the treatment if the attribution is possible, probable, or very likely according to the definitions listed below:

Possible

An adverse event that might be due to the use of the treatment. An alternative explanation, eg, concomitant drug(s), concomitant disease(s), is inconclusive. The relationship in time is reasonable; therefore, the causal relationship cannot be excluded.

Probable

An adverse event that might be due to the use of the treatment. The relationship in time is suggestive (eg, confirmed by dechallenge). An alternative explanation is less likely, eg, concomitant drug(s), concomitant disease(s).

Very Likely

An adverse event that is listed as a possible adverse reaction and cannot be reasonably explained by an alternative explanation, eg, concomitant drug(s), concomitant disease(s). The relationship in time is very suggestive (eg, it is confirmed by dechallenge and rechallenge).

Severity

An assessment of severity grade will be made using the following general categorical descriptors:

Mild: Awareness of symptoms that are easily tolerated, causing minimal discomfort and not interfering with everyday activities.

Moderate: Sufficient discomfort is present to cause interference with normal activity.

Severe: Extreme distress, causing significant impairment of functioning or incapacitation. Prevents normal everyday activities.

The investigator should use clinical judgment in assessing the severity of events not directly experienced by the subject (eg, laboratory abnormalities).

12.2. Special Situations

Safety events of interest for a Janssen product that require reporting and/or safety evaluation include, but are not limited to:

- Drug exposure during pregnancy (maternal and paternal) (see Section 12.3)
- Overdose of a Janssen product
- Exposure to a Janssen product from breastfeeding
- Suspected abuse/misuse of a Janssen product
- Inadvertent or accidental exposure to a Janssen product
- Any failure of expected pharmacological action (ie, lack of effect) of a Janssen product
- Medication error involving a Janssen product (with or without patient exposure to the Janssen product, eg, name confusion)
- Suspected transmission of any infectious agent via administration of a medicinal product
- Unexpected therapeutic or clinical benefit from use of a Janssen product

These safety events may not meet the definition of an adverse event; however, from a policy perspective, they are treated in the same manner as adverse events. Special situations should be recorded on the Adverse Event page of the CRF. Any special situation that meets the criteria of a serious adverse event should be recorded on a Serious Adverse Event Report Form and be reported to the local sponsor within 24 hours of the study site's becoming aware of the event.

12.3. Pregnancy

All reports of pregnancy occurring in temporal association with the administration of a Janssen product during the study must be reported to the sponsor by the participating study-site personnel within 24 hours of their knowledge of the event using the appropriate pregnancy notification form. Abnormal pregnancy outcomes (eg, spontaneous abortion, stillbirth, and congenital anomaly) are considered serious adverse events and must be reported using the Serious Adverse Event Form. As this is an observational study, investigators should follow the guidance in the approved local labels of medications that the subject is taking regarding discontinuation of therapy in subjects who become pregnant during the study. If a subject becomes pregnant during the study, data specified in the Data Collection Schedule that is collected as part of the subject's standard-of-care will continue to be recorded in the CRF for the applicable time points.

Because the effect of the Janssen product on sperm may be unknown, pregnancies in partners of male patients exposed to the Janssen product will be reported by the participating site personnel within 24 hours of their knowledge of the event using the appropriate pregnancy notification form. Depending on local legislation this may require prior consent of the partner.

Follow-up information regarding the outcome of the pregnancy and any postnatal sequelae in the infant should be obtained where possible.

12.4. Pharmacovigilance and Reporting Procedures

The sponsor will provide appropriate pharmacovigilance training to the participating study-site personnel.

In this observational study, there is no Janssen product under study. All adverse events will be reported as described in the following sections from the first protocol procedure (informed consent) performed within the study to the last protocol assessment within the study.

The sponsor will assume responsibility for appropriate reporting of serious adverse events and significant safety information originating from the data collected for Janssen medicinal products to the Competent Authorities.

The sponsor will prepare a final report including all adverse events recorded in the CRF.

The names (and corresponding telephone numbers) of the individuals who should be contacted regarding product safety issues or and/or quality issues are listed on the Contact Information page(s), which are provided separately.

12.4.1. Reporting Procedures for Adverse Events and Pregnancies or Pregnancies in Partners for Janssen Products

All adverse events, whether serious or non-serious, related or not related, and special reporting situations are to be documented by the investigator and recorded in the CRF and in the subject's source records. If the subject is taking a Janssen product during the study, investigators must record in the CRF their opinion concerning the relationship of the adverse event to the Janssen product. Data collection should start from the first protocol procedure (informed consent)

performed within the study and will apply to all adverse events, whether serious or non-serious, that occur to the last protocol assessment within the study.

All (serious and non-serious) adverse events reported for a Janssen product should be followed-up in accordance with clinical practice. This follow-up should be recorded in the subjects' source records and documented according to sponsor instructions.

All serious adverse events and pregnancy exposures or pregnancies in partners for Janssen products should be reported directly by the investigator, within 24 hours of their knowledge of the event, to the pharmacovigilance team using an electronic Serious Adverse Event Form or a paper pregnancy notification form.

All follow-up information for serious adverse events that have not resolved by the end of the study, or by the time of subject withdrawal, must be reported directly by the investigator, within 24 hours of his/her becoming aware, to the Pharmacovigilance team by using an electronic or paper Serious Advent Report Form (or local equivalent).

When necessary, the sponsor will inform the local Competent Authorities following applicable requirements for expedited and aggregated reporting.

12.4.2. Reporting Procedures for Serious Adverse Events and Pregnancies or Pregnancies in Partners for Non-Janssen Medicinal Products

During the study, all adverse events, whether serious or non-serious, reported following administration of a non-Janssen medicinal product (ie, a product that is not marketed by Janssen) are to be recorded in the CRF. Wherever possible, the investigator should also document his/her opinion concerning the relationship of the adverse event to the respective medicinal product.

For serious adverse events and pregnancy exposures or pregnancies in partners following exposure to a non-Janssen medicinal product (ie, a product that is not marketed by Janssen), the investigator should notify the appropriate Competent Authority (or the manufacturer of that medicinal product in the absence of appropriate local legislation) as soon as possible.

12.5. Product Quality Complaint Handling

A product quality complaint (PQC) is defined as any suspicion of a product defect related to manufacturing, labelling, or packaging, ie, any dissatisfaction relative to the identity, quality, durability, or reliability of a product, including its labelling or package integrity. A PQC may have an impact on the safety and efficacy of the product. Timely, accurate, and complete reporting and analysis of PQC information from studies are crucial for the protection of subjects, investigators, and the sponsor, and are mandated by regulatory agencies worldwide. The sponsor has established procedures in conformity with regulatory requirements worldwide to ensure appropriate reporting of PQC information; all studies conducted by the sponsor or its affiliates will be conducted in accordance with those procedures.

Product Quality Complaints for Janssen Products

All initial PQCs involving a Janssen product must be reported to the sponsor by the participating site personnel within 24 hours after being made aware of the event. The names (and corresponding telephone numbers) of the individuals who should be contacted regarding PQCs for a Janssen product are listed on the Contact Information page(s), which are provided separately.

If the defect for a Janssen product is combined with a serious adverse event, the study-site personnel must report the PQC to the sponsor according to the serious adverse event reporting timelines (see Section 12.4.1). A sample of the suspected product should be maintained for further investigation if requested by the sponsor.

Product Quality Complaints for Non-Janssen Products

Product quality complaints involving a non-Janssen medicinal product should be reported to the identified contact or manufacturer, as necessary per local regulations.

13. STUDY-SPECIFIC MATERIALS

The investigator will be provided with the following supplies:

- Study-specific smartphones (LogPad®) (including subject-completed assessments), training materials, and/or manuals
- Study-specific SitePad® (including clinician- and subject-completed assessments), training materials, and/or manuals
- Actigraphy devices (Actiwatch®) and instructions
- Paper-based clinician-completed assessments, including MINI, MGH-ATRQ, and CIRS
- Electronic data capture (eDC) manual
- Laboratory manual, including sample collection instructions
- Sample ICF

14. ETHICAL ASPECTS

14.1. Study-Specific Design Considerations

Potential subjects will be fully informed of the risks and requirements of this observational study and, during the study, subjects will be given any new information that may affect their decision to continue participation. They will be told that their consent to participate in the study is voluntary and may be withdrawn at any time with no reason given and without penalty or loss of benefits to which they would otherwise be entitled. Only subjects who are fully able to understand the risks, benefits, and potential adverse events of the study, and provide their consent voluntarily, will be enrolled.

Given that this is an observational study, potential participants will be informed that there will be no intervention provided by the study and that they will be followed over time and receive their

ongoing treatment for depression from their treating physician. The informed consent form will make it clear that there is no expected direct benefit to the subject from their participation in the study; however, the data from this study may provide important information to guide the management and follow-up of other patients with MDD in the future. Though every effort has been made to reduce subject burden, it will also be emphasized during the consent process that there are many assessments included in the study, both at regular clinic visits and subject-completed assessments between visits. To reduce the associated inconvenience, assessments between visits are staggered. Table 2 in Section 3.2 provides information for the scales included and the frequency of assessment. In addition, every effort will be maintain to ensure there is no loss of privacy by entering data using the LogPad. No personal identifiers will be used, and study data will only be available to study personnel.

As described, subjects will be asked to complete various assessments between study visits as well as at regular study visits at the study site every 8 weeks. Subjects who relapse may continue to be followed in the study, after appropriate treatment has been initiated, as long as they are willing to continue their participation and it is determined to be clinically appropriate.

There are no specific risks associated with participating in the study or completing study assessments, and further information about specific tests will be provided in the informed consent form.

The total blood volume to be collected (250 mL for 1 year in the study) is considered to be safe and appropriate for the patient population.

14.2. Regulatory Ethics Compliance

14.2.1. Investigator Responsibilities

The investigator is responsible for ensuring that the study is performed in accordance with the protocol, current ICH guidelines on Good Clinical Practice (GCP), and applicable regulatory and country-specific requirements.

Good Clinical Practice is an international ethical and scientific quality standard for designing, conducting, recording, and reporting studies that involve the participation of human subjects. Compliance with this standard provides public assurance that the rights, safety, and well-being of study subjects are protected, consistent with the principles that originated in the Declaration of Helsinki, and that the study data are credible.

14.2.2. Independent Ethics Committee or Institutional Review Board

Before the start of the study, the investigator (or sponsor where required) will provide the IEC/IRB with current and complete copies of the following documents (as required by local regulations):

- Final protocol and, if applicable, amendments
- Sponsor-approved ICF (and any other written materials to be provided to the subjects)

- Sponsor-approved subject recruiting materials
- Information on compensation for study-related injuries or payment to subjects for participation in the study, if applicable
- Investigator's curriculum vitae or equivalent information (unless not required, as documented by the IEC/IRB)
- Information regarding funding, name of the sponsor, institutional affiliations, other potential conflicts of interest, and incentives for subjects
- Any other documents that the IEC/IRB requests to fulfill its obligation

This study will be undertaken only after the IEC/IRB has given full approval of the final protocol, amendments (if any, excluding the ones that are purely administrative, with no consequences for subjects, data or study conduct), the ICF, applicable recruiting materials, and subject compensation programs, and the sponsor has received a copy of this approval. This approval letter must be dated and must clearly identify the IEC/IRB and the documents being approved.

During the study the investigator (or sponsor where required) will send the following documents and updates to the IEC/IRB for their review and approval, where appropriate:

- Protocol amendments (excluding the ones that are purely administrative, with no consequences for subjects, data or study conduct)
- Revision(s) to ICF and any other written materials to be provided to subjects
- If applicable, new or revised subject recruiting materials approved by the sponsor
- Revisions to compensation for study-related injuries or payment to subjects for participation in the study, if applicable
- Summaries of the status of the study at intervals stipulated in guidelines of the IEC/IRB (at least annually)
- Reports of serious adverse events
- New information that may adversely affect the safety of the subjects or the conduct of the study
- Deviations from or changes to the protocol to eliminate immediate hazards to the subjects
- Report of deaths of subjects under the investigator's care
- Notification if a new investigator is responsible for the study at the site
- Any other requirements of the IEC/IRB

For all protocol amendments (excluding the ones that are purely administrative, with no consequences for subjects, data or study conduct), the amendment and applicable ICF revisions must be submitted promptly to the IEC/IRB for review and approval before implementation of the change(s).

At least once a year, the IEC/IRB will be asked to review and reapprove this study. The reapproval should be documented in writing (excluding the ones that are purely administrative, with no consequences for subjects, data, or study conduct).

At the end of the study, the investigator (or sponsor where required) will notify the IEC/IRB about the study completion (if applicable, the notification will be submitted through the head of investigational institution).

14.2.3. Informed Consent

Each subject must give written consent according to local requirements after the nature of the study has been fully explained. The ICF(s) must be signed before performance of any study-related activity. The ICF(s) that is/are used must be approved by both the sponsor and by the reviewing IEC/IRB and be in a language that the subject can read and understand. The informed consent should be in accordance with principles that originated in the Declaration of Helsinki, current ICH and GCP guidelines, applicable regulatory requirements, and sponsor policy.

Before enrollment in the study, the investigator or an authorized member of the study-site personnel must explain to potential subjects the aims, methods, reasonably anticipated benefits, and potential hazards of the study, and any discomfort participation in the study may entail. Subjects will be informed that their participation is voluntary and that they may withdraw consent to participate at any time. They will be informed that choosing not to participate will not affect the care the subject will receive. Finally, they will be told that the investigator will maintain a subject identification register for the purposes of long-term follow-up if needed and that their records may be accessed by health authorities and authorized sponsor personnel without violating the confidentiality of the subject, to the extent permitted by the applicable law(s) or regulations. By signing the ICF the subject is authorizing such access, and agrees to allow his or her study physician to recontact the subject for the purpose of obtaining consent for additional safety evaluations, if needed, and subsequent disease-related treatments.

The subject will be given sufficient time to read the ICF and the opportunity to ask questions. After this explanation and before entry into the study, consent should be appropriately recorded by means of the subject's personally dated signature. After having obtained the consent, a copy of the ICF must be given to the subject.

Where local regulations require, a separate ICF may be used for the required DNA component of the study.

14.2.4. Privacy of Personal Data

The collection and processing of personal data from subjects enrolled in this study will be limited to those data that are necessary to fulfill the objectives of the study.

These data must be collected and processed with adequate precautions to ensure confidentiality and compliance with applicable data privacy protection laws and regulations. Appropriate technical and organizational measures to protect the personal data against unauthorized disclosures or access, accidental or unlawful destruction, or accidental loss or alteration must be

put in place. Sponsor personnel whose responsibilities require access to personal data agree to keep the identity of subjects confidential.

The informed consent obtained from the subject includes explicit consent for the processing of personal data and for the investigator/institution to allow direct access to his or her original medical records (source data/documents) for study-related monitoring, audit, IEC/IRB review, and regulatory inspection. This consent also addresses the transfer of the data to other entities and to other countries.

The subject has the right to request through the investigator access to his or her personal data and the right to request rectification of any data that are not correct or complete. Reasonable steps will be taken to respond to such a request, taking into consideration the nature of the request, the conditions of the study, and the applicable laws and regulations.

Exploratory DNA and biomarker research is not conducted under standards appropriate for the return of data to subjects. In addition, the sponsor cannot make decisions as to the significance of any findings resulting from exploratory research. Therefore, exploratory research data will not be returned to subjects or investigators, unless required by law or local regulations. Privacy and confidentiality of data generated in the future on stored samples will be protected by the same standards applicable to all other clinical data.

14.2.5. Long-Term Retention of Samples for Additional Future Research

Samples collected in this study may be stored for up to 15 years (or according to local regulations) for additional research. Samples will only be used to understand MDD, and to develop tests/assays related to MDD or relapse. The research may begin at any time during the study or the post-study storage period.

Stored samples will be coded throughout the sample storage and analysis process and will not be labeled with personal identifiers. Subjects may withdraw their consent for their samples to be stored for research (refer to Section 10.2, Withdrawal From the Study (Withdrawal From the Use of Samples in Future Research).

14.2.6. Country Selection

This study will only be conducted in the United States.

15. ADMINISTRATIVE REQUIREMENTS

15.1. Protocol Amendments

Neither the investigator nor the sponsor will modify this protocol without a formal amendment by the sponsor. All protocol amendments must be issued by the sponsor, and signed and dated by the investigator. Protocol amendments must not be implemented without prior IEC/IRB approval, or when the relevant competent authority has raised any grounds for non-acceptance, except when necessary to eliminate immediate hazards to the subjects, in which case the amendment must be promptly submitted to the IEC/IRB and relevant competent authority. Documentation of amendment approval by the investigator and IEC/IRB must be provided to the

sponsor. When the change(s) involves only logistic or administrative aspects of the study, the IRB (and IEC where required) only needs to be notified.

During the course of the study, in situations where a departure from the protocol is unavoidable, the investigator or other physician in attendance will contact the appropriate sponsor representative (see Contact Information page(s) provided separately). Except in emergency situations, this contact should be made before implementing any departure from the protocol. In all cases, contact with the sponsor must be made as soon as possible to discuss the situation and agree on an appropriate course of action. The data recorded in the CRF and source documents will reflect any departure from the protocol, and the source documents will describe this departure and the circumstances requiring it.

15.2. Regulatory Documentation

15.2.1. Regulatory Approval/Notification

This protocol and any amendment(s) must be submitted to the appropriate regulatory authorities in each respective country, if applicable. A study may not be initiated until all local regulatory requirements are met. This observational study will use commercially available data collection devices and equipment. There are no investigational devices being used. Sample collection is not invasive, does not require an invasive sampling procedure that presents significant risk, and will not be used as a diagnostic procedure during the study. As such, an IDE/IND is not required in order to conduct clinical sample collection or clinical study execution.

15.2.2. Required Prestudy Documentation

The following documents must be provided to the sponsor before shipment of study materials to the study site:

- Protocol and amendment(s), if any, signed and dated by the principal investigator
- A copy of the dated and signed (or sealed, where appropriate per local regulations), written IEC/IRB approval of the protocol, amendments, ICF, any recruiting materials, and if applicable, subject compensation programs. This approval must clearly identify the specific protocol by title and number and must be signed (or sealed, where appropriate per local regulations) by the chairman or authorized designee.
- Name and address of the IEC/IRB, including a current list of the IEC/IRB members and their function, with a statement that it is organized and operates according to GCP and the applicable laws and regulations. If accompanied by a letter of explanation, or equivalent, from the IEC/IRB, a general statement may be substituted for this list. If an investigator or a member of the study-site personnel is a member of the IEC/IRB, documentation must be obtained to state that this person did not participate in the deliberations or in the vote/opinion of the study.
- Signed and dated statement of investigator (eg, Form FDA 1572), if applicable
- Documentation of investigator qualifications (eg, curriculum vitae)

- Completed investigator financial disclosure form from the principal investigator, where required
- Signed and dated clinical trial agreement, which includes the financial agreement
- Any other documentation required by local regulations

The following documents must be provided to the sponsor before enrollment of the first subject:

- Completed investigator financial disclosure forms from all subinvestigators
- Documentation of subinvestigator qualifications (eg, curriculum vitae)

15.3. Subject Identification, Enrollment, and Screening Logs

The investigator agrees to complete a subject identification and enrollment log to permit easy identification of each subject during and after the study. This document will be reviewed by the sponsor study-site contact for completeness.

The subject identification and enrollment log will be treated as confidential and will be filed by the investigator in the study file. To ensure subject confidentiality, no copy will be made. All reports and communications relating to the study will identify subjects by subject identification and date of birth. In cases where the subject is not randomized into the study, the date seen and date of birth will be used.

The investigator must also complete a subject screening log, which reports on all subjects who were seen to determine eligibility for inclusion in the study.

15.4. Source Documentation

At a minimum, source documentation must be available for the following to confirm data collected in the CRF: subject identification, eligibility, and study identification; study discussion and date of signed informed consent; dates of visits; results of safety and efficacy parameters as required by the protocol; record of all adverse events and follow-up of adverse events; concomitant medication; and date of study completion and reason for early withdrawal from the study, if applicable.

In addition, the author of an entry in the source documents should be identifiable.

At a minimum, the type and level of detail of source data available for a subject should be consistent with that commonly recorded at the study site as a basis for standard medical care. Specific details required as source data for the study will be reviewed with the investigator before the study and will be described in the monitoring guidelines (or other equivalent document).

The following data will be recorded directly into the CRF and will be considered source data:

- Race

- Personal and family psychiatric history

Subject- and clinician-completed scales and assessments designated by the sponsor (subject assessments: P-FIBS, Depression IAT, CHRT, SHAPS, $QIDS\text{-}SR_{16}$, GAD-7, MOS Sleep-R, speech and voice, WHODAS 2.0, EQ-5D-5L, PSS; clinician assessments: CHRT, MADRS, CGI-S, HRUQ) will be recorded directly into the SitePad and/or LogPad and will be considered source data. Data from these devices will be transferred directly to the vendor's database. These data will be available to the site manager and study site staff through the vendor's study-specific web portal.

The minimum source documentation requirements for Section 4.1, Inclusion Criteria, and Section 4.2, Exclusion Criteria, that specify a need for documented medical history are as follows:

- Referral letter from treating physician
- Complete history of medical notes at the site
- Discharge summaries

Inclusion and exclusion criteria not requiring documented medical history must be verified at a minimum by subject interview or other protocol-required assessment (eg, physical examination, laboratory assessment) and documented in the source documents.

15.5. Case Report Form Completion

Case report forms are provided for each subject in electronic format.

Electronic Data Capture (eDC) will be used for this study. The study data will be transcribed by study-site personnel from the source documents onto an electronic CRF, and transmitted in a secure manner to the sponsor within the timeframe agreed upon between the sponsor and the study site. The electronic file will be considered to be the CRF.

Worksheets may be used for the capture of some data to facilitate completion of the CRF. Any such worksheets will become part of the subject's source documentation. All data relating to the study must be recorded in CRFs prepared by the sponsor. Data must be entered into CRFs in English. Study site personnel must complete the CRF as soon as possible after a subject visit, and the forms should be available for review at the next scheduled monitoring visit.

All subjective measurements (eg, pain scale information or other questionnaires) will be completed by the same individual who made the initial baseline determinations whenever possible. The investigator must verify that all data entries in the CRFs are accurate and correct.

All CRF entries, corrections, and alterations must be made by the investigator or other authorized study-site personnel. If necessary, queries will be generated in the eDC tool. The investigator or study-site personnel must adjust the CRF (if applicable) and complete the query.

If corrections to a CRF are needed after the initial entry into the CRF, this can be done in 3 different ways:

- Study site personnel can make corrections in the eDC tool at their own initiative or as a response to an auto query (generated by the eDC tool).
- Study site manager can generate a query for resolution by the study-site personnel.
- Clinical data manager can generate a query for resolution by the study-site personnel.

15.6. Data Quality Assurance/Quality Control

Steps to be taken to ensure the accuracy and reliability of data include the selection of qualified investigators and appropriate study sites, review of protocol procedures with the investigator and study-site personnel before the study, periodic monitoring visits by the sponsor, and direct transmission of electronic patient-reported outcome (ePRO), Actiwatch, and central laboratory data into the sponsor's data base. Written instructions will be provided for collection, handling, storage, and shipment of samples.

Guidelines for CRF completion will be provided and reviewed with study-site personnel before the start of the study.

The sponsor will review CRFs for accuracy and completeness during on-site monitoring visits and after transmission to the sponsor; any discrepancies will be resolved with the investigator or designee, as appropriate. After upload of the data into the study database they will be verified for accuracy and consistency with the data sources.

15.7. Record Retention

In compliance with the ICH/GCP guidelines, the investigator/institution will maintain all CRFs and all source documents that support the data collected from each subject, as well as all study documents as specified in ICH/GCP Section 8, Essential Documents for the Conduct of a Clinical Trial, and all study documents as specified by the applicable regulatory requirement(s). The investigator/institution will take measures to prevent accidental or premature destruction of these documents.

Essential documents must be retained until at least 2 years after the last approval of a marketing application in an ICH region and until there are no pending or contemplated marketing applications in an ICH region or until at least 2 years have elapsed since the formal discontinuation of clinical development of the investigational product. These documents will be retained for a longer period if required by the applicable regulatory requirements or by an agreement with the sponsor. It is the responsibility of the sponsor to inform the investigator/institution as to when these documents no longer need to be retained.

If the responsible investigator retires, relocates, or for other reasons withdraws from the responsibility of keeping the study records, custody must be transferred to a person who will accept the responsibility. The sponsor must be notified in writing of the name and address of the

new custodian. Under no circumstance shall the investigator relocate or dispose of any study documents before having obtained written approval from the sponsor.

If it becomes necessary for the sponsor or the appropriate regulatory authority to review any documentation relating to this study, the investigator/institution must permit access to such reports.

15.8. Monitoring

The sponsor will perform on-site monitoring visits as frequently as necessary. The monitor will record dates of the visits in a study site visit log that will be kept at the study site. The first post-initiation visit will be made as soon as possible after enrollment has begun. At these visits, the monitor will compare the data entered into the CRFs with the hospital or clinic records (source documents). The nature and location of all source documents will be identified to ensure that all sources of original data required to complete the CRF are known to the sponsor and study-site personnel and are accessible for verification by the sponsor study-site contact. If electronic records are maintained at the study site, the method of verification must be discussed with the study-site personnel.

Direct access to source documentation (medical records) must be allowed for the purpose of verifying that the data recorded in the CRF are consistent with the original source data. Findings from this review of CRFs and source documents will be discussed with the study-site personnel. The sponsor expects that, during monitoring visits, the relevant study-site personnel will be available, the source documentation will be accessible, and a suitable environment will be provided for review of study-related documents. The monitor will meet with the investigator on a regular basis during the study to provide feedback on the study conduct.

15.9. Study Completion/Termination

15.9.1. Study Completion

The study is considered completed with the last visit for the last subject participating in the study. The final data from the study site will be sent to the sponsor (or designee) after completion of the final subject visit at that study site, in the time frame specified in the Clinical Trial Agreement.

15.9.2. Study Termination

The sponsor reserves the right to close the study site or terminate the study at any time for any reason at the sole discretion of the sponsor. Study sites will be closed upon study completion. A study site is considered closed when all required documents and study supplies have been collected and a study-site closure visit has been performed.

The investigator may initiate study-site closure at any time, provided there is reasonable cause and sufficient notice is given in advance of the intended termination.

Reasons for the early closure of a study site by the sponsor or investigator may include but are not limited to:

- Failure of the investigator to comply with the protocol, the requirements of the IEC/IRB or local health authorities, the sponsor's procedures, or GCP guidelines
- Inadequate recruitment of subjects by the investigator

15.10. On-Site Audits

Representatives of the sponsor's clinical quality assurance department may visit the study site at any time during or after completion of the study to conduct an audit of the study in compliance with regulatory guidelines and company policy. These audits will require access to all study records, including source documents, for inspection and comparison with the CRFs. Subject privacy must, however, be respected. The investigator and study-site personnel are responsible for being present and available for consultation during routinely scheduled study-site audit visits conducted by the sponsor or its designees.

Similar auditing procedures may also be conducted by agents of any regulatory body, either as part of a national GCP compliance program or to review the results of this study in support of a regulatory submission. The investigator should immediately notify the sponsor if he or she has been contacted by a regulatory agency concerning an upcoming inspection.

15.11. Use of Information and Publication

All information, including but not limited to information regarding antidepressant medications and methods to determine relapse in MDD or the sponsor's operations (eg, basic scientific data, prior clinical data) supplied by the sponsor to the investigator and not previously published, and any data, including genetic, epigenetic, transcriptomic, or exploratory biomarker research data, generated as a result of this study, are considered confidential and remain the sole property of the sponsor. The investigator agrees to maintain this information in confidence and use this information only to accomplish this study, and will not use it for other purposes without the sponsor's prior written consent.

The investigator understands that the information developed in the study will be used by the sponsor in connection with the continued development of antidepressant medications and methods to determine relapse in MDD, and thus may be disclosed as required to other clinical investigators or regulatory agencies. To permit the information derived from the clinical studies to be used, the investigator is obligated to provide the sponsor with all data obtained in the study.

The results of the study will be reported in a Clinical Study Report generated by the sponsor and will contain CRF data from all study sites that participated in the study, and direct transmission of ePRO, Actiwatch, and central laboratory data into the sponsor's database. Recruitment performance or specific expertise related to the nature and the key assessment parameters of the study will be used to determine a coordinating investigator. Results of pharmacogenomic or exploratory biomarker analyses performed after the Clinical Study Report has been issued will be reported in a separate report and will not require a revision of the Clinical Study Report. Study subject identifiers will not be used in publication of results. Any work created in connection with performance of the study and contained in the data that can benefit from

copyright protection (except any publication by the investigator as provided for below) shall be the property of the sponsor as author and owner of copyright in such work.

Consistent with Good Publication Practices and International Committee of Medical Journal Editors guidelines, the sponsor shall have the right to publish such primary (multicenter) data and information without approval from the investigator. The investigator has the right to publish study site-specific data after the primary data are published. If an investigator wishes to publish information from the study, a copy of the manuscript must be provided to the sponsor for review at least 60 days before submission for publication or presentation. Expedited reviews will be arranged for abstracts, poster presentations, or other materials. If requested by the sponsor in writing, the investigator will withhold such publication for up to an additional 60 days to allow for filing of a patent application. In the event that issues arise regarding scientific integrity or regulatory compliance, the sponsor will review these issues with the investigator. The sponsor will not mandate modifications to scientific content and does not have the right to suppress information. For multicenter study designs and substudy approaches, secondary results generally should not be published before the primary endpoints of a study have been published. Similarly, investigators will recognize the integrity of a multicenter study by not submitting for publication data derived from the individual study site until the combined results from the completed study have been submitted for publication, within 12 months of the availability of the final data (tables, listings, graphs), or the sponsor confirms there will be no multicenter study publication. Authorship of publications resulting from this study will be based on the guidelines on authorship, such as those described in the Uniform Requirements for Manuscripts Submitted to Biomedical Journals, which state that the named authors must have made a significant contribution to the design of the study or analysis and interpretation of the data, provided critical review of the paper, and given final approval of the final version.

Registration of Clinical Studies and Disclosure of Results

The sponsor will register and/or disclose the existence of and the results of clinical studies as required by law.

REFERENCES

1. Akerblad A-C, Bengtsson F, von Knorring, L, Ekselius, L. Response, remission and relapse in relation to adherence in primary care treatment of depression: a 2-year outcome study. Int Clin Psychopharmacol. 2006;21:117-124.
2. Bair MJ, Robinson RL, Eckert GJ, Stang PE, Croghan TW, Kroenke K. Impact of pain on depression treatment response in primary care. Psychosomat Med. 2004;66:17-22.
3. Birnbaum HG, Kessler RC, Kelley D, Ben-Hamadi R, Joish VN, Greenberg PE. Employer burden of mild, moderate, and severe major depressive disorder: mental health services utilization and costs, and work performance. Depression Anxiety. 2010;27:78-89.
4. Bremner JD, Vermetten E, Mazure CM. Development and preliminary psychometric properties of an instrument for the measurement of childhood trauma: The Early Trauma Inventory. Depress Anxiety. 2000;12:1-12.
5. Bremner JD, Bolus R, Mayer EA. Psychometric properties of the Early Trauma Inventory-Self Report. J Nerv Ment Dis. 2007;194:211-218.
6. Brooks R, with the EuroQol Group. EuroQol: the current state of play. Health Policy. 1996;37:53-72.
7. Cohen S, Kamarck T, Mermelstein R. A global measure of perceived stress. J Health Soc Behav. 1983;24(4):385-396.
8. Clerici M, Arioso B, Mundo E, et al. Cytokine polymorphisms in the pathophysiology of mood disorders. CNS Spectr. 2009;14(8):419-425.
9. dela Cruz AM, Bernstein IH, Greer TL, et al. Self-rated measure of pain frequency, intensity, and burden: psychometric properties of a new instrument for the assessment of pain. J Psychiatr Res., in press.
10. EuroQol Group. About EQ-5D. http://www.euroqol.org/about-eq-5d.html (accessed 18 June 2014).
11. EuroQol Group. EQ-5D-5L User Guide: basic information on how to use the EQ-5D-5L instrument. Version 2.0; October 2013. http://www.euroqol.org/fileadmin/user_upload/Documenten/PDF/Folders_Flyers/UserGuide_EQ-5D-5L_v2.0_October_2013.pdf (accessed 6 August 2014).
12. Felger JC, Lotrich FE. Inflammatory cytokines in depression: neurobiological mechanisms and therapeutic implications. Neuroscience. 2013;246:199-229.
13. Garriock HA, Kraft JB, Shyn SI, et al. A genome-wide association study of citalopram response in major depressive disorder. Biol Psychiatry. 2010;67:133-138.
14. Gemar MC, Sezal ZV, Sagrati S, Kennedy SJ. Mood-induced changes on the Implicit Association Test in recovered depressed patients. J Abnorm Psychol. 2001;110:282-289.
15. Greenwald AG, McGhee DE, Schwartz JL. Measuring individual differences in implicit cognition: the implicit association test. J Pers Soc Psychol. 1998;74:1464-1480.
16. Guintivano J, Arad M, Gould TD, Payne JL, Kaminsky ZA. Antenatal prediction of postpartum depression with blood DNA methylation biomarkers. Mol Psychiatry. 2014;19:560-567.
17. Guintivano J, Brown T, Newcomer A, et al. Identification and replication of a combined epigenetic and genetic biomarker predicting suicide and suicidal behaviors. Am J Psychiatry. 2014 Jul 30. doi: 10.1176/appi.ajp.2014.1401008. [Epub ahead of print].
18. Guy W. ECDEU Assessment Manual for Psychopharmacology. Rockville, MD: U.S. Department of Health, Education, and Welfare; 1976.
19. Holmes TH, Rahe RH. The Social Readjustment Rating Scale. J Psychosom Res. 1967;11:213-218.
20. Hosang GM, Shiles C, Tansey KE, McGuffin P, Uher R. Interaction between stress and the BDNF Val66Met polymorphism in depression: a systematic review and meta-analysis. BMC Med. 2014;12:7.
21. Ising M, Lucae S, Binder EB, et al. A genomewide association study points to multiple loci that predict antidepressant drug treatment outcome in depression. Arch Gen Psychiatry. 2009;66:966-975.
22. James KE, White RF, Kraemer HC. Repeated split sample validation to assess logistic regression and recursive partitioning: an application to the prediction of cognitive impairment. Stat Med. 2005;24:3019-3035.

23. Kasper S, Hajak G, Wulff K, et al. Efficacy of the novel antidepressant agomelatine on the circadian rest-activity cycle and depressive and anxiety symptoms in major depressive disorder: a randomized double-blind comparison with sertraline. J Clin Psychiatry. 2010;71:109-120.

24. Kozma CM, Dickson M, Phillips AL, Meletiche DM. Medication possession ratio: implications of using fixed and variable observation periods in assessing adherence with disease-modifying drugs in patients with multiple sclerosis. Patient Pref Adherence. 2013;7:509-516.

25. Laje G, Lally N, Mathews D, et al. Brain-derived neurotrophic factor Val66Met polymorphism and antidepressant efficacy of ketamine in depressed patients. Biol Psychiatry. 2012;72(11):e27-e28. doi:10.1016/j.biopsych.2012.05.031.

26. Lauderdale DS, Knutson KL, Yan LL, Liu K, Rathouz PJ. Sleep duration: how well do self-reports reflect objective measure? The CARDIA Sleep Study. Epidemiology. 2008;19:838-845.

27. Lee KY, Jeong SH, Kim SH, et al. Genetic role of BDNF Val66Met and 5-HTTLPR polymorphisms on depressive disorder. Psychiatr Investig. 2014;11(2):192-199.

28. Lichtblau N, Schmidt FM, Schumann R, Kirkby KC, Himmerich H. Cytokines as biomarkers in depressive disorder: current standing and prospects. Int Rev Psychiatry. 2013;25(5):592-603.

29. Lopresti AL, Maker GL, Hood SD, Drummond PD. A review of peripheral biomarkers in major depression: the potential of inflammatory and oxidative stress biomarkers. Prog Neuro-Psychopharmacol Biol Psychiatry. 2014;48:102-111.

30. Luciano LV, Ayuso-Mateos JL, Aquado J, et al. The 12-item World Health Organization Disability Assessment Schedule II (WHO-DAS II): a nonparametric item response analysis. BMC Med Res Methodology. 2010;10:45.

31. Major Depressive Disorder Working Group of the Psychiatric GWAS Consortium. A mega-analysis of genome-wide association studies for major depressive disorder. Mol Psychiatry. 2013;18:497-511.

32. McHugh SM, Roman S, Davis B, et al. Effects of genetic variation in the P2RX7 gene on pharmacodynamics of a $P2X_7$ receptor antagonist: a prospective genotyping approach. Br J Clin Pharmacol. 2012;74(2):376-380.

33. McMahon FJ, Buervenich S, Charney D, et al. Variation in the gene encoding the serotonin 2A receptor is associated with outcome of antidepressant treatment. Am J Hum Genet. 2006;78:804-814.

34. Medical Outcomes Systems. The M.I.N.I. https://www.medical-outcomes.com/index/mini (Accessed 27 Aug 2014).

35. Meites TM, Deveney CM, Steele KT, Holmes AJ, Pizzagalli DA. Implicit depression and hopelessness in remitted depressed individuals. Behav Res Ther. 2008;46:1078-1084.

36. Miller MA, Rahe RH. Life changes scaling for the 1990s. J Psychomat Res. 1997;43(3):279-292.

37. Miller MD, Paradis CF, Houck PR, et al. Rating chronic medical illness burden in geropsychiatric practice and research: application of the cumulative illness rating scale. Psychiatr Res. 1992;41:237-248.

38. Molendijk ML, Spinhoven P, Polak M, Bus BAA, Penninx BWWJH, Elzinga BM. Serum BDNF concentrations as peripheral manifestations of depression: evidence from a systematic review and meta-analyses on 179 associations (N = 9484). Mol Psychiatry. 2014;19:791-800.

39. Montgomery SA, Asberg M. A new depression scale designed to be sensitive to change. Br J Psychiatry. 1979;134:382-389.

40. Montgomery SA, Kennedy SH, Burrows GD, Lejoyeux M, Hindmarch I. Absence of discontinuation symptoms with agomelatine and occurrence of discontinuation symptoms with paroxetine: a randomized, double-blind, placebo-controlled discontinuation study. Int Clin Psychopharmacol. 2004;19:271-280.

41. Morris DW, Toups M, Trivedi MH. Measurement-based care in the treatment of clinical depression. Focus 2012;X(4):428-433.

42. Mundt JC, Vogel AP, Feltner DE, Lenderking WR. Vocal acoustic biomarkers of depression severity and treatment response. Biol Psychiatry. 2012;72:580-587.

43. Munroe SM, Harkness KL. Life stress, the “kindling” hypothesis, and the recurrence of depression: considerations from a life stress perspective. Psychol Rev. 2005;112:417-445.

44. Nierenberg AA, Husain MM, Trivedi, MH, et al. Residual symptoms after remission of major depressive disorder with citalopram and risk of relapse: a STAR*D report. Psychol Med. 2010;40:41-50.

45. Paddock S, Laje G, Charney D, et al. Association of GRIK4 with outcome of antidepressant treatment in the STAR*D cohort. Am J Psychiatry. 2007;164:1181-1188.

46. Papakostas GI, Petersen T, Majal Y, Mischoulon D, Nierenberg AA, Fava M. Quality of life assessments in major depressive disorder: a review of the literature. Gen Hosp Psychiatry. 2004;26:13-17.

47. Posner K, Oquendo MA, Gould M, Stanley B, Davies M. Columbia Classification Algorithm of Suicide Assessment (C-CASA): classification of suicidal events in the FDA's pediatric suicidal risk analysis of antidepressants. Am J Psychiatry. 2007;164:1035-1043.

48. Powell TR, McGuffin P, D'Souza UM, et al. Putative transcriptomic biomarkers in the inflammatory cytokine pathway differentiate major depressive disorder patients from control subjects and bipolar disorder patients. PLoS ONE 2014;9(3):e91076. doi:10.1371/journal/pone.0091076.

49. QualityMetric. MOS Sleep Scale. http://www.qualitymetric.com/WhatWeDo/SFHealthSurveys/MOSSleepScale/tabid/285/Default.aspx (Accessed 6 July 2014).

50. Rapaport MH, Bose A, Zheng H. Escitalopram continuation treatment prevents relapse of depressive episodes. J Clin Psychiatry. 2004;65:44-49.

51. Rush AJ, Trivedi MH, Ibrahim HM, et al. The 16-item Quick Inventory of Depressive Symptomatology (QIDS), clinical rating (QIDS-C), and self-report (QIDS-SR): a psychometric evaluation in patients with chronic major depression. Biol Psychiatry. 2003;54(5):573-583.

52. Rush AJ, Trivedi MH, Wisniewski SR, et al. Acute and longer-term outcomes in depressed outpatients requiring one or several treatment steps: a STAR*D report. Am J Psychiatry. 2006,163:1905–1917.

53. Schmidt HD, Shelton RC, Duman RS. Functional biomarkers of depression: diagnosis, treatment, and pathophysiology. Neuropsychopharmacology. 2011;36:2375-2394.

54. Schneider B, Prvulovic D. Novel biomarkers in major depression. Curr Opinion Psychiatry. 2013;26:47-53.

55. Schroeter ML, Sacher J, Steiner J, Schoenknecht P, Mueller K. Serum S100B represents a new biomarker for mood disorders. Curr Drug Targets. 2013;14:1237-1248.

56. Segal ZV, Gemar M, Williams S. Differential cognitive response to a mood challenge following successful cognitive therapy or pharmacotherapy for unipolar depression. J Abnorm Psychol. 1999;108:3-10.

57. Snaith RP, Hamilton M, Morley S, Humayan A, Hargreaves D, Trigwell P. A scale for the assessment of hedonic tone: The Snaith-Hamilton Pleasure Scale. Br J Psychiatry. 1995;167:99-103.

58. Soskin DP, Cassiello C, Isacoff O, Fava M. The inflammatory hypothesis of depression. Focus: J Lifelong Learning Psychiatry. 2012;X (4):413-421.

59. Spitzer RL, Kroenke K, Williams JB, Lowe B. A brief measure for assessing generalized anxiety disorder: the GAD-7. Arch Intern Med. 2006;166(10):1092-1097.

60. Sullivan PF, Neale MC, Kendler KS. Genetic epidemiology of major depression: review and meta-analysis. Am J Psychiatry. 2000;157:1552-1562.

61. Trevino AC, Quateri TF, Malyska N. Phonologically-based biomarkers for major depressive disorder. EURASIP J Adv Signal Proc. 2011;42:1-18.

62. Trivedi MH, Rush AJ, Ibrahim MH, et al. The Inventory of Depressive Symptomatology, Clinician Rating (IDS-C) and Self-Report (IDS-SR), and the Quick Inventory of Depressive Symptomatology, Clinician Rating (QIDS-C) and Self-Report (QIDS-SR) in public sector patients with mood disorders: a psychometric evaluation. Psychol Med. 2004;34(1):73-82.

63. Trivedi MH, Wisniewski SR, Morris DW, et al. Concise Health Risk Tracking scale: a brief self-report and clinician rating of suicidal risk. J Clin Psychiatry. 2011;72:757-764.

64. Uher R, Perroud N, Ng MYM, et al. Genome-wide pharmacogenetics of antidepressant response in the GENDEP project. Am J Psychiatry. 2010;167:555-564.

65. University of Pittsburgh Epidemiology Data Center. IDS/QIDS: Instruments in English and multiple translations. www.ids-qids.org. Accessed 28 May 2014.

66. Valkanova V, Ebmeier KP, Allan CL. CRP, IL-6 and depression: a systematic review and meta-analysis of longitudinal studies. J Affect Dis. 2013;150:736-744.

67. van Dongen J, Jansen R, Smit D, et al. The contribution of the functional IL6R polymorphism rs2228145, eQTLs and other genome-wide SNPs to the heritability of plasma sIL-6R levels. Behav Genet. 2014;44:368-382.

68. van Hout B, Janssen MF, Feng Y-S, et al. Interim scoring for the EQ-5D-5L: mapping the EQ-5D-5L to EQ-5D-3L value sets. Value Health. 2012; 15:708-715.

69. World Health Organization. The Global Burden of Disease: 2004 Update. Geneva, Switzerland:World Health Organization. 2008. http://www.who.int/healthinfo/global_burden_disease/2004_report_update/en/index.html (accessed 9 March 2014).

70. World Health Organization. WHO Disability Assessment Schedule 2.0. http://www.who.int/classifications/icf/whodasii/en/ (Accessed 7 July 2014).

71. Williams JBW, Kobak KA. Development and reliability of a structured interview guide for the Montgomery-Asberg Depression Rating Scale (SIGMA). Br J Psychiatry. 2008;192:52-58.

72. Williamson JR, Quatieri TF, Helfer BC, Horwitz R, Yu B, Mehta DD. Vocal biomarkers of depression based on motor incoordination. Proceedings of the 3rd International Audio/Visual Emotion Challenge and Workshop: Depression and Continuous Emotion. 2013:1-7.

73. Yang SJ, Steward R, Kang H-J, et al. Response to antidepressants in major depressive disorder with melancholic features: the CRESCEND study. J Affect Dis. 2013;14:42-50.

74. Zhang C, Li, Z, Wu Z, et al. A study of N-methyl-D-aspartate receptor gene (GRIN2B) variants as predictors of treatment-resistant major depression. Psychopharmacology. 2014;231:685-693.

Attachment 1: Patient Adherence to Antidepressant Medication Questionnaire

An example of the PAQ is provided below.

Patient Adherence to Antidepressant Medication

1. How often have you taken your medication (or medications) during the last week? Please check the description that best describes your medication use.

- ☐ a. I have taken my medications every day without missing a day.
- ☐ b. I have missed taking my medications only one day.
- ☐ c. I have only missed taking my medication two days.
- ☐ d. I have missed taking my medications three or four days.
- ☐ e. I have missed taking my medications five or more days.
- ☐ f. I have stopped taking my medications.

2. Have you made any changes in how you take your medication (medications)? Please check any that apply for the past week.

- ☐ a. I have reduced my dose at times because I am feeling better.
- ☐ b. I have reduced my dose at times because of the medication's side-effects.
- ☐ c. I have increased my dose at times because I am feeling worse.
- ☐ d. I have not taken my medication as directed because I cannot afford it.
- ☐ e. I have always taken my medication as prescribed.

Mood Disorders Research Program and Clinic Copyright University of Texas Southwestern Medical Center
Final Draft

Attachment 2: Early Trauma Inventory Self Report-Short Form

An example of the ETISR-SF is provided below.

Early Trauma Inventory Self Report-Short Form (ETISR-SF)

J. Douglas Bremner, Emory University School of Medicine, Atlanta GA

Participant Name or ID:____________________ DOB:_______ Age:____ Assessment Date:__________

Part 1. General Traumas. <u>Before the age of 18</u>

1. Were you ever exposed to a life-threatening natural disaster?.................................. YES NO
2. Were you involved in a serious accident? .... YES NO
3. Did you ever suffer a serious personal injury or illness? .... YES NO
4. Did you ever experience the death or serious illness of a parent or a primary caretaker? .... YES NO
5. Did you experience the divorce or separation of your parents? .... YES NO
6. Did you experience the death or serious injury of a sibling? .... YES NO
7. Did you ever experience the death or serious injury of a friend? .... YES NO
8. Did you ever witness violence towards others, including family members? .... YES NO
9. Did anyone in your family ever suffer from mental or psychiatric illness or have a a "breakdown"? .... YES NO
10. Did your parents or primary caretaker have a problem with alcoholism or drug abuse? .... YES NO
11. Did you ever see someone murdered? .... YES NO

Part 2. Physical Punishment. <u>Before the age of 18</u>

1. Were you ever slapped in the face with an open hand? .... YES NO
2. Were you ever burned with hot water, a cigarette or something else? .... YES NO
3. Were you ever punched or kicked? .... YES NO
4. Were you ever hit with an object that was thrown at you? .... YES NO
5. Were you ever pushed or shoved? .... YES NO

Part 3. Emotional Abuse. <u>Before the age of 18</u>

1. Were you often put down or ridiculed? .... YES NO
2. Were you often ignored or made to feel that you didn't count? .... YES NO
3. Were you often told you were no good? .... YES NO
4. Most of the time were you treated in a cold, uncaring way or made to feel like you were not loved? .... YES NO
5. Did your parents or caretakers often fail to understand you or your needs?............ YES NO

Part 4. Sexual Events. <u>Before the age of 18</u>

1. Were you ever touched in an intimate or private part of your body (e.g. breast, thighs, genitals) in a way that surprised you or made you feel uncomfortable? .... YES NO
2. Did you ever experience someone rubbing their genitals against you?.................... YES NO
3. Were you ever forced or coerced to touch another person in an intimate or private part of their body? .... YES NO
4. Did anyone ever have genital sex with you against your will? .... YES NO
5. Were you ever forced or coerced to perform oral sex on someone against your will?. YES NO
6. Were you ever forced or coerced to kiss someone in a sexual rather than an affectionate way? .... YES NO

If you responded "YES" for any of the above events, answer the following for the one that has had the greatest impact on your life. In answering consider how you felt <u>at the time of the event</u>.

1. Did you experience emotions of intense fear, horror or helplessness?......................... YES NO
2. Did you feel out-of-your-body or as if you were in a dream? .... YES NO

Revised on 3/09

Attachment 3: Cumulative Illness Rating Scale

An example of the CIRS is provided below.

Patient ID |_|_|_|_|_|_|_|_|_|_|

Date |_|_| / |_|_| / |_|_|_|_|
MM DD YYYY

INSTRUCTIONS:
Refer to the CIRS manual. Write brief descriptions of the medical problem(s) that justified the endorsed score on the line following each item. (Use reverse side for more writing space)

RATING STRATEGY
0 - no problem
1 - current mild problem or past significant problem
2 - moderate disability or morbidity / requires "first line" therapy
3 - severe constant significant disability / "uncontrollable" chronic problems
4 - extremely severe / immediate treatment required / end organ failure / severe impairment in function

RATING (IF NOT PRESENT, PLEASE CHECK 0)

	0	1	2	3	4	
1.	☐	☐	☐	☐	☐	**Heart** ______
2.	☐	☐	☐	☐	☐	**Vascular** ______
3.	☐	☐	☐	☐	☐	**Haematopoietic** ______
4.	☐	☐	☐	☐	☐	**Respiratory** ______
5.	☐	☐	☐	☐	☐	**Eyes, ears, nose, throat, and larynx** ______
6.	☐	☐	☐	☐	☐	**Upper GI** ______
7.	☐	☐	☐	☐	☐	**Lower GI** ______
8.	☐	☐	☐	☐	☐	**Liver** ______
9.	☐	☐	☐	☐	☐	**Renal** ______
10.	☐	☐	☐	☐	☐	**Genitourinary** ______
11.	☐	☐	☐	☐	☐	**Musculoskeletal / Integument** ______
12.	☐	☐	☐	☐	☐	**Neurological** ______
13.	☐	☐	☐	☐	☐	**Endocrine/metabolic and breast** ______
14.	☐	☐	☐	☐	☐	**Psychiatric illness (<u>excluding MDD</u>)** ______

CRC ID |_|_|_|_|_|_|_|_|_|_|

CRS v3.0 01/01/2003

Pg. 1 of 1

Attachment 4: Massachusetts General Hospital Antidepressant Treatment Response Questionnaire

An example of the MGH-ATRQ is provided below.

MGH ANTIDEPRESSANT TREATMENT RESPONSE QUESTIONNAIRE (ATRQ)

Please indicate the correct answer to the following questions:

(1) Have you received any treatment with medications since the beginning of **THIS CURRENT** episode or period of depression? Please circle the correct answer.

YES **NO**

(2) If **YES**, please review the list on page 2 and put a check next to any medication(s) that you have taken for at least 6 or 10 weeks during THIS episode or period of depression.

(3) Of those medication(s) that you have checked from the list on page 2, please put a second check next to those that you have taken at a dosage equal to or greater than the minimum dosage listed for that medication.

(4) Of those medication(s) that you have checked from the list on page 2, please put a third check next to those that you have taken with another drug [e.g., buspirone (Buspar), lithium, psychostimulants such as methylphenidate (Ritalin), atypical antipsychotics such as olanzapine (Zyprexa)] added to augment or boost the antidepressant effect.

(5) Of the medications that you have checked on page 2, please write below the name of the one that you feel helped you the most with your depression: ____________________

(6) If a rating of 100 is "completely improved" and 0 is "not improved at all," how close to 100 did you get on this medication? Please put a check next to the answer that best applies to you.

_____ a) Less than 25% improved

_____ b) Between 25% and 49% improved

_____ c) Between 50% and 75% improved

_____ d) More than 75% improved

List of Antidepressant Medications. Instructions: Please **check** the names of any medications that you have taken for **at least 6 or 10 weeks** since the beginning of **THIS EPISODE** or period of depression. Please also **check** if your daily dosage of the medication was **equal to or greater than the minimum** dose listed below. Finally, please **check** whether a drug [e.g., buspirone (Buspar), lithium, psychostimulants such as methylphenidate (Ritalin), atypical antipsychotics such as olanzapine (Zyprexa)] was added to augment or boost the antidepressant effect.

Drug Class

Brand Name	Generic Name	At least 6 Weeks	or At least 10 Weeks	Minimum Dose	Equal or greater to	Minimum Dose	Equal or greater to	Drug was added to augment or boost effect
**Tricyclic Antidepressants**								
Adapin	doxepin	_____	_____	150mg/d	____	250mg/d	____	____
Anafranil	clomipramine	_____	_____	150mg/d	____	250mg/d	____	____
Asendin	amoxapine	_____	_____	150mg/d	____	250mg/d	____	____
Endep/Elavil	amitriptyline	_____	_____	150mg/d	____	250mg/d	____	____
Ludiomil	maprotiline	_____	_____	150mg/d	____	250mg/d	____	____
Norpramin	desipramine	_____	_____	150mg/d	____	250mg/d	____	____
Pamelor	nortriptyline	_____	_____	75mg/d	____	125mg/d	____	____
Sinequan	doxepin	_____	_____	150mg/d	____	250mg/d	____	____
Surmontil	trimipramine	_____	_____	150mg/d	____	250mg/d	____	____
Tofranil	imipramine	_____	_____	150mg/d	____	250mg/d	____	____
Vivactil	protriptyline	_____	_____	30mg/d	____	60mg/d	____	____
Azafen	pipofezine	_____	_____	150mg/d	____	300mg/d	____	____
Agedal/Elronon	noxiptiline	_____	_____	100mg/d	____	200mg/d	____	____
**Monoamine Oxidase Inhibitors (MAOIs)**								
Marplan	isocarboxazid	_____	_____	30mg/d	____	60mg/d	____	____
Nardil	phenelzine	_____	_____	45mg/d	____	90mg/d	____	____
Pamate	tranylcypromine	_____	_____	30mg/d	____	60mg/d	____	____
Emsam	selegiline patch	_____	_____	6 mg/24 hrs	____	12 mg/hrs	____	____
Aurorix	moclobemide	_____	_____	300 mg/d	____	600 mg/d	____	____
Pirazidol	pirlindole	_____	_____	200 mg/d	____	300 mg/d	____	____
**Selective Serotonin Reuptake Inhibitors (SSRIs)**								
Luvox	fluvoxamine	_____	_____	50mg/d	____	150mg/d	____	____
Paxil	paroxetine	_____	_____	20/25mg/d	____	60/75mg/d	____	____
Prozac	fluoxetine	_____	_____	20mg/d	____	60mg/d	____	____
Zoloft	sertraline	_____	_____	50mg/d	____	150mg/d	____	____
Celexa	citalopram	_____	_____	20mg/d	____	60mg/d	____	____
Lexapro	escitalopram	_____	_____	10mg/d	____	30mg/d	____	____
**Serotonin-Norepinephrine Reuptake Inhibitors (SNRIs)**								
Effexor	venlafaxine	_____	_____	150mg/d	____	250mg/d	____	____
Cymbalta	duloxetine	_____	_____	60mg/d	____	120mg/d	____	____
Pristiq	desvenlafaxine	_____	_____	50mg/d	____	100mg/d	____	____
Savella	milnacipran	_____	_____	100mg/d	____	200mg/d	____	____
Fetzima	levomilnacipran	_____	_____	40mg/d	____	120mg/d	____	____
**Other Antidepressants**								
Viibryd	vilazodone	_____	_____	40 mg/d	____	80mg/d	____	____
Desyrel	trazodone	_____	_____	300mg/d	____	600mg/d	____	____
Serzone	nefazodone	_____	_____	300mg/d	____	600mg/d	____	____
Wellbutrin	bupropion	_____	_____	300mg/d	____	450mg/d	____	____
Remeron	mirtazapine	_____	_____	15mg/d	____	45mg/d	____	____
Valdoxan	agomelatine	_____	_____	25mg/d	____	50mg/d	____	____
Stablon	tianeptine	_____	_____	37.5mg/d	____	75mg/d	____	____
Edronax	reboxetine	_____	_____	4 mg/d	____	8mg/d	____	____
Bolvidon/Depnon, Norval/Tolvon	mianserin	_____	_____	30 mg/d	____	90mg/d	____	____
Insidon	opipramol	_____	_____	150 mg/d	____	300mg/d	____	____

Did you receive electro-convulsive treatment (ECT) **during the current episode** (please circle the correct answer): YES NO

Did you **ever** receive vagal nerve stimulation (VNS) or deep brain stimulation (DBS) (please circle the correct answer): YES NO

Attachment 5: Montgomery Asberg Depression Rating Scale

An example of the MADRS is provided below.

Montgomery and Åsberg (MADRS) Depression Rating Scale

©Stuart Montgomery 1978, Measures of Depression, Fulcrum Press, London

The rating is based on a clinical interview. The rater must decide whether the rating lies on the defined scale steps (0, 2, 4, 6) or between them (I, 3, 5).

The scale to be used for the past 7 days

*1. **Apparent Sadness***

Representing despondency, gloom and despair, (more than just ordinary transient low spirits) reflected in speech, facial expression, and posture. Rate by depth and inability to brighten up.

- ☐ 0 No sadness
- ☐ 1
- ☐ 2 Looks dispirited but does brighten up without difficulty.
- ☐ 3
- ☐ 4 Appears sad and unhappy most of the time.
- ☐ 5
- ☐ 6 Looks miserable all the time or extremely despondent

*2. **Reported sadness***

Representing reports of depressed mood, regardless of whether it is reflected in appearance or not. Includes low spirits, despondency or the feeling of being beyond help and without hope. Rate according to intensity, duration and the extent to which the mood is reported to be influenced by events.

- ☐ 0 Occasional sadness in keeping with the circumstances
- ☐ 1
- ☐ 2 Sad or low but brightens up without difficulty
- ☐ 3
- ☐ 4 Pervasive feelings of sadness or gloominess. The mood is still influenced by external circumstances
- ☐ 5
- ☐ 6 Continuous or unvarying sadness, misery or despondency

3. ***Inner tension***

Representing feelings of ill-defined discomfort, edginess, inner turmoil, mental tension mounting to either panic, dread or anguish. Rate according to intensity, frequency, duration and the extent of reassurance called for.

- ☐ 0 Placid. Only fleeting inner tension
- ☐ 1
- ☐ 2 Occasional feelings of edginess and ill-defined discomfort
- ☐ 3
- ☐ 4 Continuous feelings of inner tension or intermittent panic which the patient can only master with some difficulty.
- ☐ 5
- ☐ 6 Unrelenting dread or anguish or overwhelming panic

4. ***Reduced sleep***

Representing the experience of reduced duration or depth of sleep compared to the subject's own normal pattern when well.

- ☐ 0 Sleeps as usual
- ☐ 1
- ☐ 2 Slight difficulty dropping off to sleep or slightly reduced, light or fitful sleep.
- ☐ 3
- ☐ 4 Sleep reduced or broken by at least two hours.
- ☐ 5
- ☐ 6 Less than two or three hours sleep.

5. ***Reduced appetite***
Representing the feeling of a loss of appetite compared with when well. Rate by loss of desire for food or the need to force oneself to eat.

- ☐ 0 Normal or increased appetite
- ☐ 1
- ☐ 2 Slightly reduced appetite.
- ☐ 3
- ☐ 4 No appetite or food is tasteless.
- ☐ 5
- ☐ 6 Needs persuasion to eat at all

6. ***Concentration difficulties***
Representing difficulties in collecting one's thoughts mounting to incapacitating lack of concentration. Rate according to intensity, frequency, and degree of incapacity produced.

- ☐ 0 No difficulties in concentrating
- ☐ 1
- ☐ 2 Occasional difficulties in collecting one's thoughts.
- ☐ 3
- ☐ 4 Difficulties in concentrating and sustaining thought which reduces ability to read or hold a conversation.
- ☐ 5
- ☐ 6 Unable to read or converse without great difficulty

7. ***Lassitude***
Representing a difficulty getting started or slowness initiating and performing everyday activities.

- ☐ 0 Hardly any difficulty in getting started or no sluggishness
- ☐ 1
- ☐ 2 Difficulties in starting activities
- ☐ 3
- ☐ 4 Difficulties in starting simple routine activities which are carried out with effort
- ☐ 5
- ☐ 6 Complete lassitude. Unable to do anything without help

8. ***Inability to feel***
Representing the subjective experience of reduced interest in the surroundings, or activities that normally give pleasure. The ability to react with adequate emotion to circumstances or people is reduced.

- ☐ 0 Normal interest in the surroundings and in other people
- ☐ 1
- ☐ 2 Reduced ability to enjoy usual interests
- ☐ 3
- ☐ 4 Loss of interest in the surroundings or loss of feelings for friends and acquaintances
- ☐ 5
- ☐ 6 The experience of being emotionally paralysed, inability to feel anger, grief or pleasure and a complete or even painful failure to feel for close relatives and friends.

9. ***Pessimistic thoughts***
Representing thoughts of guilt, inferiority, self-reproach, sinfulness, remorse and ruin.

- ☐ 0 No pessimistic thoughts
- ☐ 1
- ☐ 2 Fluctuating ideas of failure, self-reproach or self depreciation
- ☐ 3
- ☐ 4 Persistent self-accusations, or definite but still rational ideas of guilt or sin or increasingly pessimistic about the future
- ☐ 5
- ☐ 6 Delusions of ruin, remorse or unredeemable sin or self-accusations which are absurd and unshakable.

10. ***Suicidal thoughts***
Representing the feeling that life is not worth living, that a natural death would be welcome, suicidal thoughts, and preparations for suicide. Suicidal attempts should not in themselves influence the rating.

- ☐ 0 Enjoys life or takes it as it comes
- ☐ 1
- ☐ 2 Weary of life or only fleeting suicidal thoughts.
- ☐ 3
- ☐ 4 Probably better off dead or suicidal thoughts are common, and suicide is considered as a possible solution, but without specific plans or intention
- ☐ 5
- ☐ 6 Explicit plans for suicide when there is an opportunity or active preparations for suicide

Attachment 6: Clinical Global Impression – Severity

An example of the CGI-S is provided below.

Clinical Global Impression

Severity of illness

Considering your total clinical experience with this particular population, how mentally ill is the patient at this time?

0 = Not assessed
1 = Normal, not at all ill
2 = Borderline mentally ill
3 = Mildly ill
4 = Moderately ill
5 = Markedly ill
6 = Severely ill
7 = Among the most extremely ill patients

Reproduced from Guy W, editor. ECDEU Assessment Manual for Psychopharmacology. 1976. Rockville, MD, U.S. Department of Health, Education, and Welfare

Attachment 7: Snaith Hamilton Pleasure Scale

An example of the SHAPS is provided below.

Appendix. This scale may be reproduced under its proper title for personal use and research. Reproduction in any book or manual or for commercial purpose must be negotiated with the *British Journal of Psychiatry*.

This questionnaire is designed to measure your ability to experience pleasure *in the last few days*.
It is important to read each statement very *carefully*.
Tick *one* of the boxes [ ] to indicate how much you agree or disagree with each statement.

1. I would enjoy my favourite television or radio programme:

Strongly disagree [ ]
Disagree [ ]
Agree [ ]
Strongly agree [ ]

2. I would enjoy being with my family or close friends:

Definitely agree [ ]
Agree [ ]
Disagree [ ]
Strongly disagree [ ]

3. I would find pleasure in my hobbies and pastimes:

Strongly disagree [ ]
Disagree [ ]
Agree [ ]
Strongly agree [ ]

4. I would be able to enjoy my favourite meal:

Definitely agree [ ]
Agree [ ]
Disagree [ ]
Strongly disagree [ ]

5. I would enjoy a warm bath or refreshing shower:

Definitely agree [ ]
Agree [ ]
Disagree [ ]
Strongly disagree [ ]

6. I would find pleasure in the scent of flowers or the smell of a fresh sea breeze or freshly baked bread:

Strongly disagree [ ]
Disagree [ ]
Agree [ ]
Strongly agree [ ]

7. I would enjoy seeing other people's smiling faces:

Definitely agree	[ ]
Agree	[ ]
Disagree	[ ]
Strongly disagree	[ ]

8. I would enjoy looking smart when I have made an effort with my appearance:

Strongly disagree	[ ]
Disagree	[ ]
Agree	[ ]
Strongly agree	[ ]

9. I would enjoy reading a book, magazine or newspaper:

Definitely agree	[ ]
Agree	[ ]
Disagree	[ ]
Strongly disagree	[ ]

10. I would enjoy a cup of tea or coffee or my favourite drink:

Strongly disagree	[ ]
Disagree	[ ]
Agree	[ ]
Strongly agree	[ ]

11. I would find pleasure in small things, e.g. bright sunny day, a telephone call from a friend:

Strongly disagree	[ ]
Disagree	[ ]
Agree	[ ]
Strongly disagree	[ ]

12. I would be able to enjoy a beautiful landscape or view:

Definitely agree	[ ]
Agree	[ ]
Disagree	[ ]
Strongly disagree	[ ]

13. I would get pleasure from helping others:

Strongly disagree	[ ]
Disagree	[ ]
Agree	[ ]
Strongly agree	[ ]

14. I would feel pleasure when I receive praise from other people:

Definitely agree	[ ]
Agree	[ ]
Disagree	[ ]
Strongly disagree	[ ]

Attachment 8: Quick Inventory of Depressive Symptomatology-Self Report-16-Item

An example of the QIDS-SR_{16} is provided below.

The Quick Inventory of Depressive Symptomatology (16-Item) (Self-Report) (QIDS-SR_{16})

Name or ID: ____________________ Date: ____________________

CHECK THE ONE RESPONSE TO EACH ITEM THAT BEST DESCRIBES YOU FOR THE PAST SEVEN DAYS.

During the past seven days...

1. Falling Asleep:

- ☐ 0 I never take longer than 30 minutes to fall asleep.
- ☐ 1 I take at least 30 minutes to fall asleep, less than half the time.
- ☐ 2 I take at least 30 minutes to fall asleep, more than half the time.
- ☐ 3 I take more than 60 minutes to fall asleep, more than half the time.

2. Sleep During the Night

- ☐ 0 I do not wake up at night.
- ☐ 1 I have a restless, light sleep with a few brief awakenings each night.
- ☐ 2 I wake up at least once a night, but I go back to sleep easily.
- ☐ 3 I awaken more than once a night and stay awake for 20 minutes or more, more than half the time.

3. Waking Up Too Early:

- ☐ 0 Most of the time, I awaken no more than 30 minutes before I need to get up.
- ☐ 1 More than half the time, I awaken more than 30 minutes before I need to get up.
- ☐ 2 I almost always awaken at least one hour or so before I need to, but I go back to sleep eventually.
- ☐ 3 I awaken at least one hour before I need to, and can't go back to sleep.

4. Sleeping Too Much:

- ☐ 0 I sleep no longer than 7-8 hours/night, without napping during the day.
- ☐ 1 I sleep no longer than 10 hours in a 24-hour period including naps.
- ☐ 2 I sleep no longer than 12 hours in a 24-hour period including naps.
- ☐ 3 I sleep longer than 12 hours in a 24-hour period including naps.

During the past seven days...

5. Feeling Sad:

- ☐ 0 I do not feel sad.
- ☐ 1 I feel sad less than half the time.
- ☐ 2 I feel sad more than half the time.
- ☐ 3 I feel sad nearly all of the time.

Please complete either 6 or 7 (not both)

6. Decreased Appetite:

- ☐ 0 There is no change in my usual appetite.
- ☐ 1 I eat somewhat less often or lesser amounts of food than usual.
- ☐ 2 I eat much less than usual and only with personal effort.
- ☐ 3 I rarely eat within a 24-hour period, and only with extreme personal effort or when others persuade me to eat.

- OR -

7. Increased Appetite:

- ☐ 0 There is no change from my usual appetite.
- ☐ 1 I feel a need to eat more frequently than usual.
- ☐ 2 I regularly eat more often and/or greater amounts of food than usual.
- ☐ 3 I feel driven to overeat both at mealtime and between meals.

Please complete either 8 or 9 (not both)

8. Decreased Weight (Within the Last Two Weeks):

- ☐ 0 I have not had a change in my weight.
- ☐ 1 I feel as if I have had a slight weight loss.
- ☐ 2 I have lost 2 pounds or more.
- ☐ 3 I have lost 5 pounds or more.

- OR -

9. Increased Weight (Within the Last Two Weeks):

- ☐ 0 I have not had a change in my weight.
- ☐ 1 I feel as if I have had a slight weight gain.
- ☐ 2 I have gained 2 pounds or more.
- ☐ 3 I have gained 5 pounds or more.

During the past seven days...

10. Concentration / Decision Making:

☐ 0 There is no change in my usual capacity to concentrate or make decisions.

☐ 1 I occasionally feel indecisive or find that my attention wanders.

☐ 2 Most of the time, I struggle to focus my attention or to make decisions.

☐ 3 I cannot concentrate well enough to read or cannot make even minor decisions.

11. View of Myself:

☐ 0 I see myself as equally worthwhile and deserving as other people.

☐ 1 I am more self-blaming than usual.

☐ 2 I largely believe that I cause problems for others.

☐ 3 I think almost constantly about major and minor defects in myself.

12. Thoughts of Death or Suicide:

☐ 0 I do not think of suicide or death.

☐ 1 I feel that life is empty or wonder if it's worth living.

☐ 2 I think of suicide or death several times a week for several minutes.

☐ 3 I think of suicide or death several times a day in some detail, or I have made specific plans for suicide or have actually tried to take my life.

13. General Interest

☐ 0 There is no change from usual in how interested I am in other people or activities.

☐ 1 I notice that I am less interested in people or activities.

☐ 2 I find I have interest in only one or two of my formerly pursued activities.

☐ 3 I have virtually no interest in formerly pursued activities.

During the past seven days...

14. Energy Level:

☐ 0 There is no change in my usual level of energy.

☐ 1 I get tired more easily than usual.

☐ 2 I have to make a big effort to start or finish my usual daily activities (for example, shopping, homework, cooking, or going to work).

☐ 3 I really cannot carry out most of my usual daily activities because I just don't have the energy.

15. Feeling Slowed Down:

☐ 0 I think, speak, and move at my usual rate of speed.

☐ 1 I find that my thinking is slowed down or my voice sounds dull or flat.

☐ 2 It takes me several seconds to respond to most questions and I'm sure my thinking is slowed.

☐ 3 I am often unable to respond to questions without extreme effort.

16. Feeling Restless:

☐ 0 I do not feel restless.

☐ 1 I'm often fidgety, wringing my hands, or need to shift how I am sitting.

☐ 2 I have impulses to move about and am quite restless.

☐ 3 At times, I am unable to stay seated and need to pace around.

Attachment 9: Generalized Anxiety Disorder 7-Item Scale

An example of the GAD-7 is provided below.

**Over the last 7 days, how often have you been bothered by the following problems?** *(Use "✓" to indicate your answer)*	Not at all	Several days	More than half the days	Nearly every day
1. Feeling nervous, anxious or on edge	0	1	2	3
2. Not being able to stop or control worrying	0	1	2	3
3. Worrying too much about different things	0	1	2	3
4. Trouble relaxing	0	1	2	3
5. Being so restless that it is hard to sit still	0	1	2	3
6. Becoming easily annoyed or irritable	0	1	2	3
7. Feeling afraid as if something awful might happen	0	1	2	3

Copyright © Pfizer Inc. All rights reserved. Developed by Drs. Robert L. Spitzer, Janet B Williams, and Kurt Kroenke.
Adapted with permission of developer, July 2014

Developed by Drs. Robert L. Spitzer, Janet B.W. Williams, Kurt Kroenke and colleagues, with an educational grant from Pfizer Inc. No permission required to reproduce, translate, display or distribute.

Attachment 10: Perceived Stress Scale

An example of the PSS is provided below.

Perceived Stress Scale

The questions in this scale ask you about your feelings and thoughts **during the last month**. In each case, you will be asked to indicate by circling *how often* you felt or thought a certain way.

Name ______________________ Date ________

Age ________ Gender (*Circle*): **M F** Other ______________________

0 = Never 1 = Almost Never 2 = Sometimes 3 = Fairly Often 4 = Very Often

1. In the last month, how often have you been upset because of something that happened unexpectedly?	**0**	**1**	**2**	**3**	**4**
2. In the last month, how often have you felt that you were unable to control the important things in your life?	**0**	**1**	**2**	**3**	**4**
3. In the last month, how often have you felt nervous and "stressed"?	**0**	**1**	**2**	**3**	**4**
4. In the last month, how often have you felt confident about your ability to handle your personal problems?	**0**	**1**	**2**	**3**	**4**
5. In the last month, how often have you felt that things were going your way?	**0**	**1**	**2**	**3**	**4**
6. In the last month, how often have you found that you could not cope with all the things that you had to do?	**0**	**1**	**2**	**3**	**4**
7. In the last month, how often have you been able to control irritations in your life?	**0**	**1**	**2**	**3**	**4**
8. In the last month, how often have you felt that you were on top of things?	**0**	**1**	**2**	**3**	**4**
9. In the last month, how often have you been angered because of things that were outside of your control?	**0**	**1**	**2**	**3**	**4**
10. In the last month, how often have you felt difficulties were piling up so high that you could not overcome them?	**0**	**1**	**2**	**3**	**4**

Please feel free to use the *Perceived Stress Scale* for your research.

Mind Garden, Inc.
info@mindgarden.com
www.mindgarden.com

References

The PSS Scale is reprinted with permission of the American Sociological Association, from Cohen, S., Kamarck, T., and Mermelstein, R. (1983). A global measure of perceived stress. *Journal of Health and Social Behavior, 24,* 386-396.

Cohen, S. and Williamson, G. Perceived Stress in a Probability Sample of the United States. Spacapan, S. and Oskamp, S. (Eds.) *The Social Psychology of Health.* Newbury Park, CA: Sage, 1988.

Attachment 11: Medical Outcomes Study Sleep Scale, Revised, 12 Items

An example of the MOS Sleep-R is provided below.

MOS 12-Item Sleep Scale Single-Item Presentation Text Acute, United States (English)

Item Name	Instructions	Question Text	Answer Text 1	Answer Text 2	Answer Text 3	Answer Text 4	Answer Text 5
	Your Sleep						
		For each of the following questions, please select the one box that best describes your answer.					
SLEEPA_01	None	How long did it usually take for you to fall asleep during the past week?	0-15 minutes	16-30 minutes	31-45 minutes	46-60 minutes	More than 60 minutes
SLEEPA_02	None	On the average, how many hours did you sleep each night during the past week?	*The phrase* "Tap the black triangle below to select the number of hours per night" *should appear, along with a drop-down box (range 0-24) to record the # of hours**				
SLEEPA_03	How often during the past week did you…	Feel that your sleep was not quiet (moving restlessly, feeling tense, speaking, etc., while sleeping)?	All of the time	Most of the time	Some of the time	A little of the time	None of the time
SLEEPA_04	How often during the past week did you…	Get enough sleep to feel rested upon waking in the morning?	All of the time	Most of the time	Some of the time	A little of the time	None of the time
SLEEPA_05	How often during the past week did you…	Awaken short of breath or with a headache?	All of the time	Most of the time	Some of the time	A little of the time	None of the time
SLEEPA_06	How often during the past week did you…	Feel drowsy or sleepy during the day?	All of the time	Most of the time	Some of the time	A little of the time	None of the time
SLEEPA_07	How often during the past week did you…	Have trouble falling asleep?	All of the time	Most of the time	Some of the time	A little of the time	None of the time
SLEEPA_08	How often during the past week did you…	Awaken during your sleep time and have trouble falling asleep again?	All of the time	Most of the time	Some of the time	A little of the time	None of the time
SLEEPA_09	How often during the past week did you…	Have trouble staying awake during the day?	All of the time	Most of the time	Some of the time	A little of the time	None of the time
SLEEPA_10	How often during the past week did you…	Snore during your sleep?	All of the time	Most of the time	Some of the time	A little of the time	None of the time

Item Name	Instructions	Question Text	Answer Text 1	Answer Text 2	Answer Text 3	Answer Text 4	Answer Text 5
SLEEPA_11	How often during the past week did you…	Take naps (5 minutes or longer) during the day?	All of the time	Most of the time	Some of the time	A little of the time	None of the time
SLEEPA_12	How often during the past week did you…	Get the amount of sleep you needed?	All of the time	Most of the time	Some of the time	A little of the time	None of the time
		Copyright, 1986, RAND.<br>MOS 12-Item Sleep Scale Acute – Revised 2010<br>United States (English)					

* Please review the potential need to change this text to suit each e-pro vendor/product. As an alternative to this text, the following can be utilized:
The phrase “Select the number of hours per night” *should appear, along with a drop-down box (range 0-24) to record the # of hours*

Translation for this phrase is as follows: Enter translation

Copyright, 1986, RAND.
MOS 12-Item Sleep Scale Acute – Revised 2010
Single-Item Presentation Text Acute, United States (English)

Attachment 12: EuroQol Group: 5 Dimension, 5 Level

An example of the EQ-5D-5L is provided below.

EQ-5D-5L

Health Questionnaire

English version for the USA

SUBMISSION

USA (English) © 2009 EuroQol Group. EQ-5D™ is a trade mark of the EuroQol Group

Under each heading, please check the ONE box that best describes your health TODAY

MOBILITY

I have no problems walking ❑
I have slight problems walking ❑
I have moderate problems walking ❑
I have severe problems walking ❑
I am unable to walk ❑

SELF-CARE

I have no problems washing or dressing myself ❑
I have slight problems washing or dressing myself ❑
I have moderate problems washing or dressing myself ❑
I have severe problems washing or dressing myself ❑
I am unable to wash or dress myself ❑

USUAL ACTIVITIES *(e.g. work, study, housework, family or leisure activities)*

I have no problems doing my usual activities ❑
I have slight problems doing my usual activities ❑
I have moderate problems doing my usual activities ❑
I have severe problems doing my usual activities ❑
I am unable to do my usual activities ❑

PAIN / DISCOMFORT

I have no pain or discomfort ❑
I have slight pain or discomfort ❑
I have moderate pain or discomfort ❑
I have severe pain or discomfort ❑
I have extreme pain or discomfort ❑

ANXIETY / DEPRESSION

I am not anxious or depressed ❑
I am slightly anxious or depressed ❑
I am moderately anxious or depressed ❑
I am severely anxious or depressed ❑
I am extremely anxious or depressed ❑

2

USA (English) © 2009 EuroQol Group. EQ-5D™ is a trade mark of the EuroQol Group

SUBMISSION

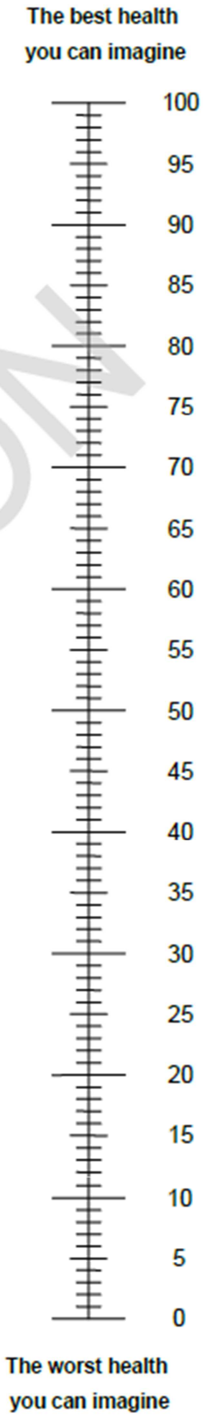

- We would like to know how good or bad your health is TODAY.
- This scale is numbered from 0 to 100.
- 100 means the best health you can imagine.
  0 means the worst health you can imagine.
- Mark an X on the scale to indicate how your health is TODAY.
- Now, please write the number you marked on the scale in the box below.

YOUR HEALTH TODAY = ☐

SUBMISSION

3

USA (English) © 2009 EuroQol Group. EQ-5D™ is a trade mark of the EuroQol Group

Attachment 13: World Health Organization Disability Assessment Schedule

An example of the WHODAS 2.0 is provided below.

WHODAS 2.0

WORLD HEALTH ORGANIZATION
DISABILITY ASSESSMENT SCHEDULE 2.0

12-item version, self-administered

This questionnaire asks about difficulties due to health conditions. Health conditions include diseases or illnesses, other health problems that may be short or long lasting, injuries, mental or emotional problems, and problems with alcohol or drugs.

Think back over the past 30 days and answer these questions, thinking about how much difficulty you had doing the following activities. For each question, please circle only one response.

In the past 30 days, how much difficulty did you have in:						
S1	Standing for long periods such as 30 minutes?	None	Mild	Moderate	Severe	Extreme or cannot do
S2	Taking care of your household responsibilities?	None	Mild	Moderate	Severe	Extreme or cannot do
S3	Learning a new task, for example, learning how to get to a new place?	None	Mild	Moderate	Severe	Extreme or cannot do
S4	How much of a problem did you have joining in community activities (for example, festivities, religious or other activities) in the same way as anyone else can?	None	Mild	Moderate	Severe	Extreme or cannot do
S5	How much have you been emotionally affected by your health problems?	None	Mild	Moderate	Severe	Extreme or cannot do

Please continue to next page...

Page 1 of 2 *(12-item, self-administered)*

WHODAS 2.0

WORLD HEALTH ORGANIZATION
DISABILITY ASSESSMENT SCHEDULE 2.0

12
Self

In the past 30 days, how much difficulty did you have in:						
S6	Concentrating on doing something for ten minutes?	None	Mild	Moderate	Severe	Extreme or cannot do
S7	Walking a long distance such as a kilometre [or equivalent]?	None	Mild	Moderate	Severe	Extreme or cannot do
S8	Washing your whole body?	None	Mild	Moderate	Severe	Extreme or cannot do
S9	Getting dressed?	None	Mild	Moderate	Severe	Extreme or cannot do
S10	Dealing with people you do not know?	None	Mild	Moderate	Severe	Extreme or cannot do
S11	Maintaining a friendship?	None	Mild	Moderate	Severe	Extreme or cannot do
S12	Your day-to-day work?	None	Mild	Moderate	Severe	Extreme or cannot do

H1	Overall, in the past 30 days, how many days were these difficulties present?	*Record number of days* ____
H2	In the past 30 days, for how many days were you totally unable to carry out your usual activities or work because of any health condition?	*Record number of days* ____
H3	In the past 30 days, not counting the days that you were totally unable, for how many days did you cut back or reduce your usual activities or work because of any health condition?	*Record number of days* ____

This completes the questionnaire. Thank you.

Page 2 of 2 *(12-item, self-administered)*

Attachment 14: Recent Life Change Stress Test (Baseline)

An example of the RLCST (Baseline) is provided below.

Recent Life Change Stress Test (RLCST)

This questionnaire asks you about life events that you recently experienced. For each event, indicate if the event happened to you **over the past year.**

**Health**		
An illness or injury which was very serious	☐ Yes	☐ No
An illness or injury which was moderately severe	☐ Yes	☐ No
An illness or injury which was less serious than above	☐ Yes	☐ No
**Work**		
Change to a new type of work	☐ Yes	☐ No
Change in your work conditions	☐ Yes	☐ No
Change in your work responsibilities	☐ Yes	☐ No
Taking courses to help you	☐ Yes	☐ No
Troubles at work	☐ Yes	☐ No
Major business readjustment	☐ Yes	☐ No
Loss of your job	☐ Yes	☐ No
Retirement	☐ Yes	☐ No
**Home and Family**		
Change in residence	☐ Yes	☐ No
Major change in living conditions	☐ Yes	☐ No
Change in family get-togethers	☐ Yes	☐ No
Major change in health or behavior of a family member	☐ Yes	☐ No
Marriage	☐ Yes	☐ No
Pregnancy	☐ Yes	☐ No
Miscarriage or abortion	☐ Yes	☐ No
Birth or adoption of a child	☐ Yes	☐ No
Spouse begins or stops work	☐ Yes	☐ No
Change in arguments with spouse	☐ Yes	☐ No
Problems with relatives or in-laws	☐ Yes	☐ No
Parents divorce	☐ Yes	☐ No
A parent remarries	☐ Yes	☐ No
Separation from spouse due to work or marital difficulties	☐ Yes	☐ No
Child leaves home	☐ Yes	☐ No
Relative moves in with you	☐ Yes	☐ No
Divorce	☐ Yes	☐ No
Birth of a grandchild	☐ Yes	☐ No

Death of spouse	☐ Yes	☐ No
Death of child	☐ Yes	☐ No
Death of parent or sibling	☐ Yes	☐ No
**Personal and Social**		
Change in personal habits	☐ Yes	☐ No
Beginning or ending of school	☐ Yes	☐ No
Change of school or college	☐ Yes	☐ No
Change in political beliefs	☐ Yes	☐ No
Change in religious beliefs	☐ Yes	☐ No
Change in social activities	☐ Yes	☐ No
Vacation	☐ Yes	☐ No
New, close, personal relationship	☐ Yes	☐ No
Engagement to marry	☐ Yes	☐ No
Personal relationship problems	☐ Yes	☐ No
Sexual difficulties	☐ Yes	☐ No
An accident	☐ Yes	☐ No
Minor violation of the law	☐ Yes	☐ No
Being held in jail	☐ Yes	☐ No
Major decision about your future	☐ Yes	☐ No
Major personal achievement	☐ Yes	☐ No
Death of a close friend	☐ Yes	☐ No
**Financial**		
Major loss of income	☐ Yes	☐ No
Major increase in income	☐ Yes	☐ No
Loss or damage to personal property	☐ Yes	☐ No
Major purchase	☐ Yes	☐ No
Minor purchase	☐ Yes	☐ No
Credit difficulties	☐ Yes	☐ No

Adapted from the online version of the Recent Life Change Stress Test ©, available at www.drrahe.com.

Attachment 15: Recent Life Change Stress Test (Since Last Visit)

An example of the RLCST (Since Last Visit) is provided below.

Recent Life Change Stress Test (RLCST)

This questionnaire asks you about life events that you recently experienced. For each event, indicate if the event happened to you **since your last visit.**

**Health**		
An illness or injury which was very serious	☐ Yes	☐ No
An illness or injury which was moderately severe	☐ Yes	☐ No
An illness or injury which was less serious than above	☐ Yes	☐ No
**Work**		
Change to a new type of work	☐ Yes	☐ No
Change in your work conditions	☐ Yes	☐ No
Change in your work responsibilities	☐ Yes	☐ No
Taking courses to help you	☐ Yes	☐ No
Troubles at work	☐ Yes	☐ No
Major business readjustment	☐ Yes	☐ No
Loss of your job	☐ Yes	☐ No
Retirement	☐ Yes	☐ No
**Home and Family**		
Change in residence	☐ Yes	☐ No
Major change in living conditions	☐ Yes	☐ No
Change in family get-togethers	☐ Yes	☐ No
Major change in health or behavior of a family member	☐ Yes	☐ No
Marriage	☐ Yes	☐ No
Pregnancy	☐ Yes	☐ No
Miscarriage or abortion	☐ Yes	☐ No
Birth or adoption of a child	☐ Yes	☐ No
Spouse begins or stops work	☐ Yes	☐ No
Change in arguments with spouse	☐ Yes	☐ No
Problems with relatives or in-laws	☐ Yes	☐ No
Parents divorce	☐ Yes	☐ No
A parent remarries	☐ Yes	☐ No
Separation from spouse due to work or marital difficulties	☐ Yes	☐ No
Child leaves home	☐ Yes	☐ No
Relative moves in with you	☐ Yes	☐ No
Divorce	☐ Yes	☐ No
Birth of a grandchild	☐ Yes	☐ No

Death of spouse	☐ Yes	☐ No
Death of child	☐ Yes	☐ No
Death of parent or sibling	☐ Yes	☐ No
**Personal and Social**		
Change in personal habits	☐ Yes	☐ No
Beginning or ending of school	☐ Yes	☐ No
Change of school or college	☐ Yes	☐ No
Change in political beliefs	☐ Yes	☐ No
Change in religious beliefs	☐ Yes	☐ No
Change in social activities	☐ Yes	☐ No
Vacation	☐ Yes	☐ No
New, close, personal relationship	☐ Yes	☐ No
Engagement to marry	☐ Yes	☐ No
Personal relationship problems	☐ Yes	☐ No
Sexual difficulties	☐ Yes	☐ No
An accident	☐ Yes	☐ No
Minor violation of the law	☐ Yes	☐ No
Being held in jail	☐ Yes	☐ No
Major decision about your future	☐ Yes	☐ No
Major personal achievement	☐ Yes	☐ No
Death of a close friend	☐ Yes	☐ No
**Financial**		
Major loss of income	☐ Yes	☐ No
Major increase in income	☐ Yes	☐ No
Loss or damage to personal property	☐ Yes	☐ No
Major purchase	☐ Yes	☐ No
Minor purchase	☐ Yes	☐ No
Credit difficulties	☐ Yes	☐ No

Adapted from the online version of the Recent Life Change Stress Test ©, available at www.drrahe.com.

Attachment 16: Pain, Frequency, Intensity, and Burden Scale

An example of the P-FIBS is provided below.

Indicate how you feel by circling one number on the scale for each question (items 1-4).

1. How frequently have you experienced pain in the past week?

Never		Some of the Days		About Half of the Days		Most of the Days		Everyday
0	1	2	3	4	5	6	7	8

2. How would you rate the intensity of your pain in the past week?

No Pain		Mild Pain		Moderate Pain		Severe Pain		Unbearable Pain
0	1	2	3	4	5	6	7	8

3. How much did pain interfere with your daily life in the past week?

Never		Some of the Days		About Half of the Days		Most of the Days		Everyday
0	1	2	3	4	5	6	7	8

4. How often did you use medication or other treatment to manage your pain in the past week?

Never		Some of the Days		About Half of the Days		Most of the Days		Everyday
0	1	2	3	4	5	6	7	8

© 2009 UT Southwestern Medical Center at Dallas.

Attachment 17: First Symptom of Relapse - Self-Assessment

An example of the FSR-SA is provided below.

First Symptom of Relapse – Self Assessment (FSR-SA)

This question is intended to collect information to understand the change(s) around the time your depression became worse, and how you experienced this. Please think about how you have been feeling and how your depression has been, considering all the ways it affects your health and wellbeing. Describe below the first sign, symptom or indication you noticed that made you aware of your worsening depression.

Please describe below or enter "none" if appropriate:

____

____

____

____

____

____

How many weeks ago was this?

- ☐ **1 week**
- ☐ **2 weeks**
- ☐ **3 weeks**
- ☐ **4 weeks**
- ☐ **5 weeks**
- ☐ **6 weeks**
- **Other** ☐☐ **weeks**

Attachment 18: Concise Health Risk Tracking Scale (Baseline)

An example of the CHRT (Baseline) is provided below.

Concise Health Risk Tracking (CHRT)
with Clinician Rated Behavioral Module

Please rate the extent to which each of the following statements describes how you have been feeling or acting in the past week.

For example, if you feel the statement very accurately describes how you have been feeling in the past week, you would give a rating of "Strongly Agree." If you feel the statement is not at all how you have been feeling in the past week, you would give a rating of "Strongly Disagree."

		Strongly Disagree (0)	Disagree (1)	Neither Agree nor Disagree (2)	Agree (3)	Strongly Agree (4)
1.	I feel as if things are never going to get better.	☐	☐	☐	☐	☐
2.	I have no future.	☐	☐	☐	☐	☐
3.	It seems as if I can do nothing right.	☐	☐	☐	☐	☐
4.	Everything I do turns out wrong.	☐	☐	☐	☐	☐
5.	There is no one I can depend on.	☐	☐	☐	☐	☐
6.	The people I care the most for are gone.	☐	☐	☐	☐	☐
7.	I wish my suffering could just all be over.	☐	☐	☐	☐	☐
8.	I feel that there is no reason to live.	☐	☐	☐	☐	☐
9.	I wish I could just go to sleep and not wake up.	☐	☐	☐	☐	☐
10.	I find myself saying or doing things without thinking.	☐	☐	☐	☐	☐
11.	I often make decisions quickly or "on impulse."	☐	☐	☐	☐	☐
12.	I often feel irritable or easily angered.	☐	☐	☐	☐	☐
13.	I often overreact with anger or rage over minor things.	☐	☐	☐	☐	☐
14.	I have been having thoughts of killing myself.	☐	☐	☐	☐	☐
15.	I have thoughts about how I might kill myself.	☐	☐	☐	☐	☐
16.	I have a plan to kill myself.	☐	☐	☐	☐	☐

Propensity Score (sum items 1-13): _________

Risk Score (sum items 14-16): _________

Version 3.0

© UT Southwestern Medical Center, 2008

Concise Health Risk Tracking (CHRT) - Clinician Rated Behavioral Module

Ask patient the questions in *bold italics*. Please rate the patient's behavior over the past week.

YES	NO	#	Item
☐	☐	1	**Suicidal Ideation** - Passive (i.e. wanting to be dead) and/or active (i.e. method, intent, plan) SI present.<br>***This last week did you think you might be better off dead or wish you were dead?***<br>***Did you have any thoughts of harming or injuring yourself in any way?***<br>If Yes: ***Have you thought about how you might do this?***<br>***Have there been times when you seriously considered harming or injuring yourself?***<br>***Do you intend to kill yourself or harm yourself in any way? Do you have a plan?***<br>***How often have you had these thoughts? How long do they last?***<br>***Do you have any difficulty controlling those thoughts?***<br>Level of Ideation<br>☐ Passive ☐ Active w/method ☐ Active w/method, intent ☐ Active w/method, intent, plan<br>Frequency of Ideation<br>☐ Less than once a week ☐ Once a week ☐ 2-5 times per week ☐ Daily<br>Duration of Ideation<br>☐ Few seconds ☐ < 1 hour on < 4 days ☐ 1 to 4 hours on ≥ 4 days ☐ > 4 hours daily<br>Controllability of Ideation<br>☐ Easy to control ☐ Minor difficulty controlling ☐ Significant effort to control ☐ Cannot control
☐	☐	2.	**Suicide Attempt** - Patient made a suicide attempt (i.e. they engaged in a potentially self-injurious behavior associated with intent to die. Intent can be stated by patient or inferred by rater).<br>***This last week did you attempt to harm or injure yourself in any way?***<br>If Yes: ***Can you tell me what happened? Was this an accident or on purpose?***<br>If On Purpose: ***Why did you _____? Were you trying to kill yourself when you _____?***<br>If Yes, list method: ______________________________
☐	☐	3.	**Self-injurious Behavior - No Intent to Die** - Purposeful self-injurious behavior with no intent to die.<br>***This last week, have you done anything to prepare yourself for suicide or take any steps towards killing yourself?***<br>If Yes: ***What did you do? Were you thinking about killing yourself when you _____?***<br>***Did you stop yourself, or did someone else stop you before you harmed yourself?***
☐	☐	4.	**Preparatory Acts** - Making preparatory acts toward imminent suicidal behavior (Person takes steps to injure self but is stopped by self or others. Intent to die is either stated by patient or inferred by rater).
☐	☐	5.	**Completed Suicide** - Confirmed (i.e. Coroner's report, suicide note, other collateral information).
☐	☐	6.	**Self-injurious Behavior - Unknown Intent** - Purposeful self-injurious behavior where associated intent to die is unknown and cannot be inferred.
☐	☐	7.	**Death (not enough information to classify as suicide)**
☐	☐	8.	**Other Injury** - Other not purposeful injury (accidental, psychiatric, medical), no deliberate self-harm).
☐	☐	9.	***Nonfatal Injury (not enough information to* classify)**

Version 3.0

© UT Southwestern Medical Center, 2008

Attachment 19: Concise Health Risk Tracking Scale (Since Last Visit)

An example of the CHRT (Since Last Visit) is provided below.

Concise Health Risk Tracking (CHRT)
with Clinician Rated Behavioral Module

Please rate the extent to which each of the following statements describes how you have been feeling or acting since your last visit.

For example, if you feel the statement very accurately describes how you have been feeling since your last visit, you would give a rating of "Strongly Agree." If you feel the statement is not at all how you have been feeling since your last visit, you would give a rating of "Strongly Disagree."

		Strongly Disagree (0)	Disagree (1)	Neither Agree nor Disagree (2)	Agree (3)	Strongly Agree (4)
1.	I feel as if things are never going to get better.	☐	☐	☐	☐	☐
2.	I have no future.	☐	☐	☐	☐	☐
3.	It seems as if I can do nothing right.	☐	☐	☐	☐	☐
4.	Everything I do turns out wrong.	☐	☐	☐	☐	☐
5.	There is no one I can depend on.	☐	☐	☐	☐	☐
6.	The people I care the most for are gone.	☐	☐	☐	☐	☐
7.	I wish my suffering could just all be over.	☐	☐	☐	☐	☐
8.	I feel that there is no reason to live.	☐	☐	☐	☐	☐
9.	I wish I could just go to sleep and not wake up.	☐	☐	☐	☐	☐
10.	I find myself saying or doing things without thinking.	☐	☐	☐	☐	☐
11.	I often make decisions quickly or "on impulse."	☐	☐	☐	☐	☐
12.	I often feel irritable or easily angered.	☐	☐	☐	☐	☐
13.	I often overreact with anger or rage over minor things.	☐	☐	☐	☐	☐
14.	I have been having thoughts of killing myself.	☐	☐	☐	☐	☐
15.	I have thoughts about how I might kill myself.	☐	☐	☐	☐	☐
16.	I have a plan to kill myself.	☐	☐	☐	☐	☐

Propensity Score (sum items 1-13): _________

Risk Score (sum items 14-16): _________

Version 3.0

© UT Southwestern Medical Center, 2008

Concise Health Risk Tracking (CHRT) - Clinician Rated Behavioral Module

Ask patient the questions in *bold italics*. Please rate the patient's behavior since the last visit.

YES	NO	#	Item
YES ☐	NO ☐	1	**Suicidal Ideation** - Passive (i.e. wanting to be dead) and/or active (i.e. method, intent, plan) SI present.<br>***Since your last visit did you think you might be better off dead or wish you were dead?***<br>***Did you have any thoughts of harming or injuring yourself in any way?***<br>If Yes: ***Have you thought about how you might do this?***<br>***Have there been times when you seriously considered harming or injuring yourself?***<br>***Do you intend to kill yourself or harm yourself in any way? Do you have a plan?***<br>***How often have you had these thoughts? How long do they last?***<br>***Do you have any difficulty controlling those thoughts?***<br>Level of Ideation<br>☐ Passive ☐ Active w/method ☐ Active w/method, intent ☐ Active w/method, intent, plan<br>Frequency of Ideation<br>☐ Less than once a week ☐ Once a week ☐ 2-5 times per week ☐ Daily<br>Duration of Ideation<br>☐ Few seconds ☐ < 1 hour on < 4 days ☐ 1 to 4 hours on ≥ 4 days ☐ > 4 hours daily<br>Controllability of Ideation<br>☐ Easy to control ☐ Minor difficulty controlling ☐ Significant effort to control ☐ Cannot control
YES ☐	NO ☐	2.	**Suicide Attempt** - Patient made a suicide attempt (i.e. they engaged in a potentially self-injurious behavior associated with intent to die. Intent can be stated by patient or inferred by rater).<br>***Since your last visit did you attempt to harm or injure yourself in any way?***<br>If Yes: ***Can you tell me what happened? Was this an accident or on purpose?***<br>If On Purpose: ***Why did you _____? Were you trying to kill yourself when you _____?***<br>If Yes, list method: ____________________
YES ☐	NO ☐	3.	**Self-injurious Behavior - No Intent to Die** - Purposeful self-injurious behavior with no intent to die.<br>***Since your last visit, have you done anything to prepare yourself for suicide or take any steps towards killing yourself?***<br>If Yes: ***What did you do? Were you thinking about killing yourself when you _____?***<br>***Did you stop yourself, or did someone else stop you before you harmed yourself?***
YES ☐	NO ☐	4.	**Preparatory Acts** - Making preparatory acts toward imminent suicidal behavior (Person takes steps to injure self but is stopped by self or others. Intent to die is either stated by patient or inferred by rater).
YES ☐	NO ☐	5.	**Completed Suicide** - Confirmed (i.e. Coroner's report, suicide note, other collateral information).
YES ☐	NO ☐	6.	**Self-injurious Behavior - Unknown Intent** - Purposeful self-injurious behavior where associated intent to die is unknown and cannot be inferred.
YES ☐	NO ☐	7.	**Death (not enough information to classify as suicide)**
YES ☐	NO ☐	8.	**Other Injury** - Other not purposeful injury (accidental, psychiatric, medical), no deliberate self-harm).
YES ☐	NO ☐	9.	***Nonfatal Injury (not enough information to* classify)**

Version 3.0

© UT Southwestern Medical Center, 2008

INVESTIGATOR AGREEMENT

I have read this protocol and agree that it contains all necessary details for carrying out this study. I will conduct the study as outlined herein and will complete the study within the time designated.

I will provide copies of the protocol and all pertinent information to all individuals responsible to me who assist in the conduct of this study. I will discuss this material with them to ensure that they are fully informed regarding the study drug, the conduct of the study, and the obligations of confidentiality.

Coordinating Investigator (where required):

Name (typed or printed): ______________________

Institution and Address: ______________________

____

____

____

Signature: ______________________ Date: ______________________

(Day Month Year)

Principal (Site) Investigator:

Name (typed or printed): ______________________

Institution and Address: ______________________

____

____

____

Telephone Number: ______________________

Signature: ______________________ Date: ______________________

(Day Month Year)

Sponsor's Responsible Medical Officer:

Name (typed or printed): Ella Daly, MD

Institution: Janssen Research & Development, LLC

Signature: electronic signature appended at the end of the protocol Date: ______________________

(Day Month Year)

Note: If the address or telephone number of the investigator changes during the course of the study, written notification will be provided by the investigator to the sponsor, and a protocol amendment will not be required.

LAST PAGE

SIGNATURES

Signed by	Date	Justification
Ella Daly	05Sep2014, 01:30:45 AM, UTC	Document Approval
