# Supplementary material for: Personalized relapse prediction in patients with major depressive disorder using digital biomarkers
Source: Sci Rep. 2023 Oct 30;13:18596. doi: 10.1038/s41598-023-44592-8 (PMC10616277; doi:10.1038/s41598-023-44592-8)
Supplement: Supplementary file 2 — Supplementary Information 2. [file 41598_2023_44592_MOESM2_ESM.pdf]

Study Title:

A collaborative investigation of predictors of relapse in major depressive disorder: CAN-BIND-1 extension study
(Wellness Monitoring for Major Depressive Disorder)

Principal Investigator:
Sidney H. Kennedy, MD, FRCPC
PPD

UHN Co-Investigators:
PPD

Other Site Co-Principal Investigators
PPD

Other Site Co-Investigator:
PPD

Study Funder:
Ontario Brain Institute (OBI)

Clinical Research Partner:
Janssen Research & Development, LLC

1. Background Information

Prevalence and the Risk of Recurrence in Major Depressive Disorder

Major Depressive Disorder (MDD) is a prevalent and debilitating illness, and an estimated 1 in 5 people experience at least one major depressive episode (MDE) during their lifetime (Kessler et al, 2003). Despite high rates of response over 12 weeks of antidepressant treatment (Uher, et al, 2013) there is an 85% risk of recurrence within 15 years of a MDE (Mueller et al, 1999) and residual depressive symptoms occur in 60% of cases during long-term follow up (Judd et al. 1998). Biomarkers of remission and relapse have yet to be elucidated. Identifying such biomarkers could allow for early clinical intervention and treatment adjustment to effectively prevent remission or relapse.

In recent years, mobile or remote biosensors have become useful tools while managing individuals with multiple and complex medical conditions. The use of such technologies is already enabling patints to be monitored remotely , thus avoiding long waiting lines, diminishing travel costs and time, and reducing burden incurred by multiple visits to health care servies. The use of mobile-based technologies has already shown some promising results among individuals with mental health conditions. Studies have indicated significant contributions to health care support and symptom reduction in adolescent depression (Reid et al., 2013) and schizophrenia (Granholm et al., 2012); and in some instances, contributed to improved treatment adherence (Free et al., 2013).

Summary of CAN-BIND-1

The Canadian Biomarker Integration Network in Depression (CAN-BIND) multi-center study was devised to address the knowledge gap in relation to the ability to predict treatment response (and remission) in MDD. Detailed clinical, neuroimaging, molecular and genetic data from patients with MDD are being obtained to explore biomarkers that may predict response to treatment for MDD (Kennedy et al, 2012). The CAN-BIND-1 study involves treatment of MDD outpatients aged 18 to 55 years of age, with a MADRS score ≥ 24 and episode duration of > 3 months at study entry. Patients receive 8-week open label treatment with escitalopram (10-20mg/day). Assessment of responder status (≥ 50% reduction on MADRS from baseline) is determined at 8 weeks. Responders to escitalopram continue for a second 8-week period during which they remain on escitalopram and are followed clinically. Non-responders to escitalopram receive add-on pharmacotherapy with aripiprazole (2-10mg/day), an evidence based augmentation strategy for a second 8-week period. Clinical, plasma and urine biomarker evaluations are conducted 0, 2, 8 and 16 weeks, and imaging and EEG at 0, 2 and 8 weeks.

Rationale for Extension Study

This extension study is a prospective, longitudinal, observational study aimed at identifying biomarkers of relapse in MDD. The protocol closely mirrors the OBSERVEMDD001 study run by Janssen R&D LLC, which also has the aim of identifying biomarkers of relapse in a population of patients with MDD who are currently responding to treatment or combination of treatments. This extension study will involve naturalistic follow up of responders from the study entitled “Integrated biological markers for the prediction of treatment response in depression”, or the CAN-BIND-1 Study. The study will be conducted in partnership with Janssen Research & Development and will utilize remote monitoring technology for data gathering in addition to data collected at study visits; study data will be shared and analyzed

collaboratively. Measures collected during the CAN-BIND-1 Study and during this extension study will be evaluated alone and in combination to identify predictors of relapse. In addition, the study is also open to other participants who completed other CAN-BIND studies, as well as remitters who meet the inclusion criteria. Since patients usually seek medical attention only after a relapse has occurred, imminent precursors to relapse are not well known. In this extension study, participants who are currently responding to an oral antidepressant treatment regimen and/or therapeutic intervention will be monitored over a minimum period of 13 months, which provides a unique opportunity to discover near-term biomarkers of relapse. Results may refine the clinical approach to relapse management, and may ultimately help MDD patients sustain wellness while on antidepressant medication.

2. Study Objectives and Hypothesis

Primary Objective

The primary objective of this study is to collect a broad range of clinical data during a period of follow up of individuals who are currently responding to treatment or a combination of treaments to monitor for relapse and subsequently to evaluate the data for potential biomarkers of relapse. Domains to be assessed include mood and anxiety symptoms, anhedonia, sleep, pain, quality of life, stressful life events, treatment compliance, activity, and speech and voice characteristics. Self-report measures, clinician-rated assessments, voice capture and objective measures via actigraphy will be used to assess these domains.

Study Hypothesis

The collection and evaluation of clinical data during this study, and if available, paired with a rich set of clinical, imaging and molecular data collected during the CAN-BIND-1 study may reveal biomarkers of relapse for MDD.

Previously collected biomarker data from other CAN-BIND studies may also be available upon mutual agreement of all investigators and collaborators involved. Amendment to any existing Research Collaboration Agreement(s) and/or a new Research Collaboration Agreement is necessary.

3. Study Design

Study Design Summary

This will be an exploratory, prospective, observational study in which a range of variables will be measured and evaluated as potential predictors of relapse. Some study subjects who are currently undergoing treatment for MDD (e.g., oral antidepressant medication, cognitive behavioural therapy, etc., or a combination of treatments) will continue their treatment as prescribed by their treating physician during the study. Others that may have responded may not necessarily be on any treatment at the time of study enrolment, but responded to a treatment intervention in the previous MDD episode

and are continuing to respond at the time of enrolment. There will be no study-related treatments or interventions and no coverage for therapies will be provided.

After providing written informed consent, participants will receive a study-specific smartphone (LogPad®) and a study-specific wrist-worn device, the GT9X Link.

In addition, a subset of the population with eligible smartphones will be asked to download an app called HealthRhythms® (HR) to their personal devices and continuously use it for their entire study participation. They will be asked to uninstall the app upon study completion or pre-mature withdrawal. The HealthRhythms® mobile health app is a smartphone app that continuously records data via the participant's mobile phone. This mobile health app was developed through a collaborative partnership between experienced researchers in mental health and innovators in mobile technology. It has been tested in a large study conducted at the University of Pittsburgh (Abdullah et al, In Press).

The study will consist of a screening phase of up to 2 weeks and an observational phase of variable duration, with study visits every 8 weeks. Subjects who relapse during the observational phase study will continue in the observational phase, with study visits continuing every 8 weeks, until relapse is observed in a number of participants that is determined by interim analysis or until the end of the 1-year enrolment period for the last-subject-in, whichever comes first. The total duration for each study subject will therefore be vatriable. Concomitant medications for stable medical conditions will be allowed at the discretion of the study psychiatrist. Vitamins, supplements, birth control pills, and over the counter pain medication will also be allowed at the discretion of the study psychiatrist, but must be disclosed by the patient and noted on the file for data analysis purposes.

At baseline and every 8 weeks thereafter, participants will be assessed for depressive symptom severity, as well as information in relation to their use of healthcare services. This information will be collected using an electronic data collection device called the SitePad® during clinical visits. During the baseline and 8-week interval clinic visits, participants will also complete self-reports through Brain-CODE's REDCap interface and provide blood samples. Brain-CODE is the Ontario Brain Institute's neuroinformatics platform, further described in the Data Management section. In addition, participants will remotely complete weekly self-reports through the use of a LogPad®. Speech and voice characteristics will be assessed at two-week intervals also through the use of a LogPad®. Motor activity and sleep parameters will be captured using the GT9X Link, a wrist-worn device throughout the study period.

For non-CAN-BIND participants, we will conduct the Childhood Experience of Care and Abuse (CECA) [Bifulco, Brown, and Harris, 1994] and Life Events and Difficulties Schedule (LEDS) [Bifulco, et. al., 1989] interviews. These are one-time, semi-structured interviews. And these are designed to carefully assess the history of childhood maltreatment and the effects of stress and early life experience on neurobehavioural biomarkers in depression. Both CECA and LEDS interview will be conducted by telephone and audio-recorded. They will be subsequently coded by research assistants. Ratings will be based on manualized examples to ensure standardization and to prevent rater drift. The ratings will be entered in REDCap. Raw audio recordings will be deleted at the end of the study. Both phone interviews may be scheduled at once or may be split into two sessions and will be conducted at anytime during the course of study when it is convenient to the study participants.

All data will be collected and evaluated with a view towards identifying predictors of relapse. Scales and assessments are detailed below under “Clinical Assessments”. This study will be carried out in partnership with Janssen Research & Development, LLC. All data collected using the LogPad®, SitePad®, REDCap,GT9X Link, and HealthRhythms® app will be shared with Janssen Research & Development, LLC. Data analysis and publications arising from this study will be carried out collaboratively with Janssen Research & Development, LLC.

Dosage and Administration

If applicable, study subjects will continue to undergo their treatment regimen as recommended by their treating physician during the study. Adjustment to their treatment regimen (if applicable) will be allowed as clinically indicated, including if a subject relapses, and will be documented in the case report form. The study investigator will be required to document why a change was made to the treatment regimen. Any adjustments to the treatment regimen, such as the addition of cognitive behavioural therapy, are also allowable but require documentation as to the necessity of their usage.

Clinical Assessments

- Physical Examination
  A physical examination will be performed at screening and at the end-of-study or early withdrawal visit. Height and weight will be measured at the screening visit.

- The Mini-international Neuropsychiatric Interview (M.I.N.I.) (Sheehan, D.V., 2015)
  A short and structured diagnostic interview for DSM-V and ICD-10.

- Childhood Experience of Care and Abuse (CECA) (Bifulco, Brown, and Harris, 1994)
  A semi-structured clinician-rated interview that includes the following scales: (a) antipathy – hostility and coldness toward the child; (b) neglect – indifference to the child’s physical and emotional needs; (c) physical abuse – violence directed toward the child by patents; and (d) sexual abuse – non-consensula sexual contact by any perpetrator.

- Life Events and Difficulties Schedules (LEDS) (Bifulco, A., et.al., 1989)
  A semi-structured contextual interview rating system that assesses stressful life events that have occurred within 6 months of depression onset or past year if depression is chronic. The events captured are grouped in the following domains: education, occupation, housing, finances, role changes, legal, health, romantic relationships, other relationships, and deaths.

Clinician Assessments to be completed via SitePad® every 8 weeks

- Montgomery Asberg Depression Rating Scale (MADRS) (Montgomery and Asberg 1979)
  MADRS was the primary outcome in the CAN-BIND-1 study due to its sensitivity to treatment. MADRS will be the primary measure to evaluate depressive symptoms, including whether relapse (as defined below) has occurred. The MADRS is a 10-item clinician-rated scale designed to measure depression severity and detects changes due to antidepressant treatment.

- Clinical Global Impression – Severity (CGI-S) (Guy et al., 1976)

The CGI-S is a physician-rated scale that is designed to rate the severity of the subject's illness at the time of assessment, relative to the clinician's past experience with subjects who have the same diagnosis and improvement with treatment.

- Healthcare Resource Use Questionnaire (HRUQ)
  The HRUQ is a clinician-administered assessment developed by Janssen Research & Development. The utilization of health care services, including hospitalizations, emergency room visits, day hospital attendance, outpatient visits, and work productivity, are evaluated.

- Concise health risk tracking (CHRT) – review and score (Trivedi et al, 2011)
  The clinician will use the SitePad® to review the subject responses and to calculate the Propensity Score and Risk Score for the CHRT (described below).

- Vital Signs (Pulse/Heart Rate, Blood Pressure)
  Vital signs, including body weight, will be measured at each study visit. Blood pressure and pulse/heart rate measurements will be assessed in a supine position with a completely automated device. Manual techniques will be used only if an automated device is not available. Blood pressure and pulse/heart rate measurements should be preceded by at least 5 minutes of rest in a quiet setting without distractions (e.g., television, cell phones).

Self-reports to be completed on site via SitePad® according to the visit schedule

- Early Trauma Inventory Self Report – Short Form (ETISR-SF) (Bremner, 2007)
  The ETISR-SF is simple to administer childhood trauma scale. This 29-item scale covers physical punishment, emotional abuse and sexual events before the age of 18 and general traumas after the age of 18. The scale has good validity and internal consistency.

- Pain Frequency, Intensity, and Burden Scale (P-FIBS) (dela Cruz et al., 2014)
  The P-FIBS is a 4-item brief self-reported measure developed to assess pain in research settings or as a screening tool in clinical settings.

- Depression Implicit Association Test (Depression IAT) (Meites et al., 2008)
  The Project Implicit Mental Health (PIMH) depression-specific Implicit Association Test (IAT) is an association test designed to assess automatic cognitive processes; subjects are asked to categorize a concept (e.g., "me" vs "not me") with an attribute (e.g., "logical") by responding to stimulus words (i.e., stimulus words including synonyms as well as antonyms of the attribute being tested). Reaction time is measured; faster responding is interpreted to represent stronger automatic associations between concept and attribute.

- Concise Health Risk Tracking (CHRT) (Trivedi et al, 2011)
  The CHRT has two sections. The first is a 16-item subject self-reported screen for suicidal ideation and associated symptoms. The second is a clinician-rated behavioural module, Suicidal Behavioural Evaluation, which covers all Columbia Classification Algorithm of Suicide Assessment (C-CASA) domains and involves actively querying subjects about the occurrence of suicidal thinking.

- Snaith-Hamilton Pleasure Scale (SHAPS) (Snaith et al., 1995)

The SHAPS is a 14-item self-report scale shown to be a reliable, valid, and unidimensional instrument for assessing the hedonic capacity in patients with MDD [21 and 22]. Four domains of pleasures response are covered: interest and pastimes, social interaction, sensory experience, and food and drink.

- Recent Life Changes Stress Test (RLCST) (Miller et al., 1997)
  The RLCST is a subject-completed scale that measures the number of life change events, such as marriage or death of a family member, the subject has experienced or expects to experience in the near future.

- First Symptom of Relapse – Self-Assessment (FSR-SA)
  The FSR-SA is a single-item assessment that will be completed by all subjects who relapse, with the objective of providing additional information from the subject's perspective about potential predictors of relapse.

Self-reports to be completed on site via REDCap every 8 weeks

- Pittsburgh Sleep Quality Index (PSQI) (Buysse et al. 1989)
  The PSQI is a self-rated questionnaire specifically designed to measure sleep quality. The questionnaire includes questions on sleep duration, sleep disturbance, sleep latency, day dysfunction due to sleepiness, sleep efficiency, overall sleep quality and need for medication to sleep. This questionnaire takes less than 5 minutes to complete.

- Sheehan Disability Scale (SDS) (Sheehan et al, 1996)
  The SDS-VAS is a self-rated questionnaire that assesses functional disability and impairment due to psychiatric symptoms. The SDS-VAS consists of three functional impairment items and two items related to productivity losses due to the symptoms and impairment. Impairment is evaluated with the 10-point self-rated social life and family life/home responsibilities subscales of the Sheehan Disability Scale (0, none; 1-3, mild; 4-6, moderate; 7-9, marked; 10, extreme). Significant impairment for each subscale was defined by a rating of 7 or higher.

- Quality of Life Enjoyment and Satisfaction Questionnaire (Q-LES-Q) (Endicott et al, 1993)
  This 16-item questionnaire contains questions about your general life satisfaction on aspects such as physical health, leisure time and overall sense of wellbeing. Each item is scored on a 5-point scale (very poor to very good); higher scores indicate greater satisfaction and enjoyment. It takes 5 minutes to complete.

- Brief Symptom Inventory (BSI-53) (Derogatis et al., 1993)
  The BSI-53 is a 53-item self-rated questionnaire that assesses depression across nine symptom domains which include aspects such as obsessions, sensitivity, depression, anxiety, hostility, anxiety and paranoia. Each item is ranked on a 5-point scale from 0 (not at all) to 4 (extremely), and rankings represent intensity of distress over the past week. This scale takes 10 minutes to complete.

- Lam Employment Absence and Productivity Scale (LEAPS) (Lam et al., 2009)
  The LEAPS is a 10-item, self-rated scale that provides information on occupational functioning. The items are based on symptoms that have the most impact on work productivity. The scale

takes 3 to 5 minutes to complete and has been shown to have good internal and external validity in outpatient patients with MDD.

- Brief Diet Inventory (BDI)
  This inventory consists of five simple questions to assess dietary habits that relate to the frequency of consumption of various foods and beverages, as well as multivitamins.

- International Physical Activity Questionnaire (IPAQ) (Craig, 2003)
  The IPAQ was developed as an instrument to monitor cross-national physical activity and inactivity. It assesses physical activity across a comprehensive set of domains including leisure time, domestic and gardening activities, work-related and transport-related activity.

- World Health Organization Quality of Life Short Version (WHO-QoL-BREF) (Orley & Kuyken, 1994)
  This 26-item self-rated questionnaire that assesses your view of your quality of life across four domains including physical health, psychological health, social relationships and environment. This brief scale takes 5 minutes to complete.

- Biological Rhythms Interview of Assessment In Neuropsychiatry (BRIAN) (Giglio et al, 2009)
  This is a scale designed to assess circadian rhythms in mood disorders, including important information relating to sleep dysregulation

- Rothschild Scale for Antidepressant Tachyphylaxis (RSATTM) (Rothschild, 2008)
  It is a self-report questionnaire assessing energy level, motivation and interest, cognitive functioning, weight gain, sleep and sexual functioning. Each item is measured using a 5-point ordinal scale with anchor points.

Self-reports to be completed remotely via LogPad® at regular intervals.

Weekly assessments:

- Quick Inventory of Depressive Symptomatology-Self-Report (QIDS-SR) (Rush et al, 2003)
  The QIDS-SR is a 16 item scale used to assess the severity of depressive illness derived from the 30-item Inventory of Depressive Symptomatology (IDS). The scale includes questions on mood, sleep and level of interest, and takes 5-10 minutes to complete.

- Generalized Anxiety Disorder 7-item (GAD-7) (Spitzer et al. 2006)
  The GAD-7 is a 7-item self-rated questionnaire for assessing generalized anxiety disorder and its severity. Items are ranked on a 4-point scale from 0 (not at all sure) to 3 (nearly every day), providing a total severity score from 0 to 21. It should take 5 minutes to complete this scale.

- Medical Outcomes Study Sleep Scale, Revised, 12-items (MOS Sleep-R) (Hays et al., 2005)
  The MOS Sleep-R provides detailed information on subjective sleep behavior. The instrument have 6 subscales including sleep disturbance, snoring, shortness of breath or headache, sleep adequacy, sleep somnolence, and sleep quantity

- Patient Adherence to Antidepressant Medication Questionnaire (PAQ)

The PAQ is a brief, 2-item questionnaire that provides information regarding medication compliance.

Biweekly assessments:

- EuroQol Group; 5 dimension; 5 level (EQ-5D-5L) (Brooks R, 1996)
  The EQ-5D-5L is a standardized 2-part instrument for use as a measure of health outcome, primarily designed for self-completion by respondents.

- Speech and Voice Characteristics
  Speech and voice characteristics will be assessed using speech diary recordings via a study-specific smartphone (LogPad®). Subjects will use a software application on the smartphone to record speech at 2-week intervals. The application will prompt each subject to describe his/her physical condition during the past 2 weeks; his/her mental condition during the past 2 weeks; how his/her physical and mental feelings have affected his or her ability to function during the past 2 weeks; the event during the past 2 weeks that made him or her the happiest. Participants will also be asked to read a provided passage, pronounce vowel sounds and count numbers aloud.

Monthly assessments:

- World Health Organization Disability Assessment Schedule 2.0 (WHODAS 2.0) (Carlozzi et al., 2015)
  The WHODAS 2.0 is a generic instrument developed by the WHO to provide a standardized method for measuring health and disability across cultures.

- Perceived Stress Scale (PSS) (Cohen et al., 1983)
  The PSS is a 10-item, self-reported, unidimensional instrument developed to measure perceived stress in response to situations in a person's life. Respondents report the prevalence of an item within the last month on a 5-point scale.

Activity and Sleep Parameters via GT9X Link

Activity and sleep parameters will be measured using a GT9X Link, a wrist device. GT9X Link measures will include:

- Sleep duration (total of sleep per night in hours)
- Sleep pattern (time of sleep onset; time to sleep end)
- Sleep quality (mean activity during rest period in counts/minute; sleep fragmentation; sleep efficiency; GT9X Link estimate of wake after sleep onset in minutes)
- Daytime activity (mean daytime activity in counts per minute; peak daytime activity counts/minute)

Measures collected by the mobile health app (HealthRhythms® App)

Data will be collected through the app in the following ways: 1) Self-reports (via mobile phone); and 2) Passively collected data via mobile phone sensors.

The HR app is designed to run in the background and continuously collected phone usage data about the following:

- *On or Off state of screen* - Whether the smartphone screen is on or off, locked or unlocked.
- *Location* - Latitude/longitude coordinates (and other features) derived via Wi-Fi, cell phone towers and GPS.
- *Physical activity* - This includes pedometer data reported by the Operating System over a given time interval, as well as motion activities reported by the Operating System, derived via available hardware on the smartphone, e.g. accelerometer, gyroscope.

  Inferences from can be generated from the data collected above:
  - Travel diameter – total diameter in meters that a person travels in a day
  - Travel distance – the over all distance a person travels in a day
  - Time at home – the percentage of a day a person spends at home
  - Places visited – the location that a person spent time in
  - Physical Activity (walking rate) – number of steps per minute
  - Social Activity (voice) – percentage of voice signal per day
  - Technology use (smartphone use) – percentage of phone use per day
  - Sleep: sleep duration - number of minutes participant slept; and sleep timing – time the participant went to bed and woke up

In addition, for this particular study, participants will be occasionally prompted to complete 3 self-resport questionnaires:

- Photographic Affect Meter (PAM) (Pollak et. al., 2011)
  It is a photograph based measure of affect. See sample image below.

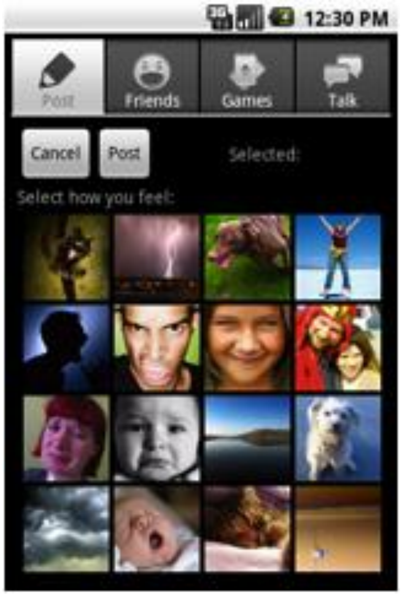

- Patient Health Questionnaire (PHQ-8) (Kroenke, K. et. a., 2009)
  It is a well-validated measure of depression
- Sleep Questionnaire, consisting of the following items:
  Q1. When did you wake up?
  Q2. When did you go to sleep last night?

Relapse Criteria

Relapse will be defined as any of the following:

- MADRS total score ≥22 for at least 2 consecutive weeks. If the relapse criterion of MADRS total score ≥22 is met at a study visit (scheduled or unscheduled), an additional visit (i.e., the Relapse Verification visit) will be scheduled within 1 to 2 weeks to verify the relapse.
- Hospitalization for worsening of depression
- Suicidal ideation with intent, or suicidal behavior
- Other (investigator will be asked to describe)

Subjects who relapse will continue to be followed in the study, i.e. the intent is to continue to follow all relapsed and non-relapsed subjects. Additional details can be found under “Follow up for relapse verification” below.

Blood Draws and Biospecimen Handling

At each 8 week visit, blood samples will also be collected. CAN-BIND has a standard biospecimen collection kit that will be assembled and distributed to each clinical site. Samples will be collected at the participating sites across Canada and will be processed and shipped on dry ice to the Douglas Hospital Research Centre, McGill University, Montreal. The DHRC biorepository is equipped for secure sample storage and for the handling of biohazardous substances such as biological fluids, tissues and cell cultures. The facility is located in an area with access restricted to authorized personnel and is staffed by technicians trained in the receipt, data entry, processing, storage and retrieval of biospecimens. The biorepository features an automated sample storage and retrieval platform (Sample Access Manager, Hamilton Storage Technologies, Inc.) offering -80°C and -20°C inert gas storage environments with current capacity for 15,000 additional samples at either temperature. These freezers are attached to a back-up power supply and are monitored by a call-out alarm system to alert staff if a drop in temperature occurs. Daily monitoring of freezer temperature and operation is conducted to ensure proper freezer performance and maintenance. Upon reception, each sample is registered and annotated into our biospecimen tracking database. This system assigns a unique identifier and allocates each sample to a specific location in the sample bank. After sample information is entered, each sample aliquot container is identified by a 2D barcode cryo-label encoding the sample identifier and location for efficient and accurate retrieval. All blood samples collected will be analyzed for the purposes of this resesearch study only. Samples from other research sites will be stored at the Douglas Hospital Research Centre and analyzed together. Pregnant women will be excluded from blood draws given added complexities to molecular analyses in this population.

Study Conclusion

At study conclusion, subjects will have an end-of-study visit conducted, and will then be referred for further follow up with their treating physician.

4. Study Population

Men and women between 18 and 65 years of age who have previously met DSM-V criteria for Major Depressive Episode (MDE) in MDD as determined by MINI, who participated in the CAN-BIND-1 protocol, and who are currently responding to antidepressant medication will be offered the opportunity to participate in this study. In addition, previous study participants from other CAN-BIND studies will be approached for the Wellness Monitoring Study. The extension study is also open to remitters of MDD and patients attending the Mood Disorders Clinic who meet the criteria. Participants who are currently responding to treatment for MDD e.g., oral antidepressants, rTMS, cognitive behavioural therapy, etc., or a combination of treatments. Participants must have a Montgomery Asberg Depression Rating Scale (MADRS) total score ≤14 at both baseline and screening visits.
Inclusion criteria

Each potential subject must satisfy all of the following criteria to be enrolled in the study.

1. Outpatients 18 to 65 years of age.
2. Meet DSM-V criteria for MDE in MDD past or recurrent episode as determined by the MINI.
3. In the current or most recent MDE, subject must be responding or responded to a treatment or a combination of treatments for MDD.
4. Subjects must have a MADRS total score ≤14.
5. Subject must be willing and able to complete self-reported assessments via a study-specific smartphone (LogPad®), including sufficient fluency in English.
6. Subject must be willing to wear a GT9X Link, wrist-worn device for the duration of the study.

Exclusion Criteria

Any potential subject who meets any of the following criteria will be excluded from participating in the study.

1. Any Axis I diagnosis, other than MDD, that is considered the primary diagnosis.
2. Bipolar I or Bipolar-II diagnosis (lifetime), MDD with psychotic features (lifetime), schizophrenia, or schizoaffective disorder.
3. Presence of a significant Axis II diagnosis (borderline, antisocial).
4. High suicidal risk, defined by clinician judgment.
5. History of drug or alcohol use, with a severity of at least moderate or severe, according to DSM-V criteria, within 6 months before screening.
6. Presence of significant neurological disorders, head trauma, or other unstable medical conditions.
7. Subject has received an investigational drug (including investigational vaccines) or used an invasive investigational medical device within 90 days before screening or is currently enrolled in an investigational study.
8. Subject has any condition for which, in the opinion of the investigator, participation would not be in the best interest of the subject (e.g., compromise the well-being) or that could prevent, limit, or confound the protocol-specified assessments.
9. Subject is an employee of the investigator or study site, with direct involvement in the proposed study or other studies under the direction of that investigator or study site, or is a family member of an employee or the investigator.

NOTE: Investigators will ensure that all study enrolment criteria have been met at screening. If a subject's status changes (including receipt of additional medical records) after screening but before the

baseline visit such that he or she no longer meets all eligibility criteria, then the subject will be excluded from participation in the study.
Recruitment:

Initial contact will be made by the CAN-BIND-1 study coordinator or study psychiatrist. The study coordinator or study psychiatrist will inform the participant of the study during his/her last CAN-BIND-1 study visit. Participants who have already exited the CAN-BIND-1 study will be contacted by phone about this study only if consent to contact was obtained on the CAN-BIND-1 consent form. The coordinator will obtain written consent from the participant to participate in this extension study. One further telephone call will be made if there is no response to the initial attempt at contact.

The same protocol will be implemented for potential participants from other CAN-BIND studies. The study investigators from other studies will introduce the Extension Study to potential participants on their last study visit. Participants who have exited other CAN-BIND studies will be contacted by the study coordinators associated with the other studies to establish a link, only if consent for future research projects was obtained. If the participant agrees to learn more about the Wellness Study, the Study Coordinator will initiate contact for screening and written informed consent.

In addition, patients attending the study investigator's Mood Disorders clinic will be screened for eligibility. The psychiatrists will only introduce the Wellness Study to the patients. The study coordinator will follow-up to avoid coercion and also discuss the study at length. The patients will be informed that if they decide not to participate, their treatment care will not be affected in anyway. The study coordinator will proceed with screening and obtaining of written informed consent if the patient agrees to participate.

Sample Size Determination:

The first patient completed CAN-BIND-1 in November2013, and at the beginning of October 2016, there were 211 patients at baseline (week 0) and 158 patients had completed the full 16 week study. Of these completers, approximately 72% are classified as responders and can be screened for this extension study. Sample size was determined by the number of participants who were eligible and consented. CAN-BIND-1 enrolled 211 MDD participants total, and of these it was expected that approximately 50% will meet criteria and agree to participate in the follow-up.
Recruitment for the Wellness Study commenced in July 2016. There are 70 active participants enrolled to-date.

5. Contracted Hardware and Software

SitePad® Dell Venue 11

The SitePad® Dell Venue 11 will be used for clinician-administered assessments and some of the 8-week interval self-reports. This device allows for data to be transmitted via Ethernet, Wi-Fi or embedded 3.5/4G. No separate transmission devices are required.

LogPad® N5
The LogPad® N5 will be used by participants to complete self-reports remotely between study visits. This consumer smartphone is a locked-down, dedicated electronic Clinical Outcome Assessment (eCOA) device. This device will also be used with a microphone (detailed below) for voice diaries.

GT9X Link, wrist-worn device
The GT9X-BT Link Bluetooth Activity Monitor device by ActiGraph® will be used to collect activity and sleep information throughout the study duration. Data will be uploaded by study coordinators during clinic visits. Study staff will also use these data to monitor participant adherence and compliance to the provisions in the protocol, in particular, the wearing and use of the equipment. Participants will be instructed to wear the GT9X Link 24 hours per day.

AKG C520 Microphone Kit
The AKG Pro Audio C520 L Head-Worn Condenser Microphone will be used in conjunction with the Griffin MicConnect Microphone Connector for speech diary recordings every two weeks. This microphone and microphone connector connect directly to the LogPad® N5.

Millisecond IAT Software Plug-in and Windows Mini USB Keyboard (KB1700)
The Implicit Association Test (IAT) software and keyboard will be used with the SitePad® for the Depression-IAT assessment only. The plug-in launches in the SitePad®, and the license is managed by Project Implicit. The keyboard functions only when this software is launched and in session.

HealthRhythms® (HR) App
The app was developed by HealthRhythms® Inc. (https://HealthRhythms®.com/en/). It is a privately owned technology company with an interest in promoting the use of mobile health technologies for wellness monitoring in individuals with mood disorders. HealthRhythms® is a for-profit organization focused on improving the assessment and treatment of mental health. The app is designed to constantly run in the background, automatically collecting information that can be used to measure mood, social, and sleep patterns throughout the day and night. Janssen will be providing HealthRhythms mobile app license for the study participants enrolled in the study as needed.

6. Data Management

Data Management Overview
Subjects will use the LogPad®, SitePad® and REDCap to enter self-report data as per the Time and Events Schedule in Appendix 1. Clinicians and/or approved study personnel will use the SitePad® to enter participant interview data as per the Time and Events Schedule in Appendix 1. CECA and LEDS ratings will be entered into REDCap by research staff. Actigraph data will be collected continuously throughout the study using the GT9X Link ,awrist-worn device. Data from all platforms will be centrally stored in the Ontario Brain Institute's Brain-CODE platform for researcher access. Data from REDCap will be entered directly into and managed in the Brain-CODE platform. Data from the LogPad® and SitePad® will be uploaded to the ERT centrally managed database and transferred to Brain-CODE at monthly intervals. Data from the GT9X Link will be uploaded to and managed in the CenterPoint System and will be transferred to Brain-CODE regularly. Approved UHN personnel will be provided with password protected access to data within Brain-CODE related to this study. All data from the LogPad®, SitePad®, REDCap, GT9X Link, and HealthRhythms® app will be shared with Janssen Research & Development, LLC.

Data Management within Ontario Brain Institute's Brain-CODE Platform

Some self-report data will be collected using the REDCap clinical trials software, and will be sent to the Ontario Brain Institute's Brain-CODE (www.braincode.ca) database, deployed at the High Performance Computer Virtual Lab (HPCVL) data centre (Kingston, Ontario). The HPCVL is a Compute Canada high performance computing consortium and the only facility of its kind in Canada that supports regulatory-compliant (e.g., 21 CFR Part 11, HIPAA, PIPEDA) processes for securing privacy of healthcare data. More specifically, bank-level security is ensured at the HPCVL through application of 256-bit encryption and issuances of Entrust certificates through the web port. HPCVL has achieved the designation of an Entrust Certificate Authority. Automated backups are performed daily (on-site) and weekly (off-site) to ensure data availability and disaster recovery. All dedicated equipment is segregated on a separate network and protected by multiple layers of security including a security appliance firewall.

Research Electronic Data Capture (REDCap) is a web-based system for data collection where participants will complete the self-report questionnaires and enter data in a web browser from remote locations (Harris et al. 2009). REDCap includes a complete suite of features to support Health Insurance Portability and Accountability Act (HIPAA) compliance, including a full audit trail, user-based privileges, and integration with the institutional server. Access to REDCap will be secured by an encrypted virtual private network and protected by multiple levels of authentication. A Data Manager will be assigned administrative privileges for study configuration, design of CRFs, data entry management, and quality control. The data management team will include qualified database administrators to program scripted and ad-hoc data queries, data backup and technical support. Access to the study's data in REDCap will be restricted to the members of the study team by username and password.

This system will be used for data entry, data monitoring and cleaning, and for the export of datasets for statistical analysis and predictive modeling. eCRFs employ valid, reliable and widely used scales and interviews in the mood disorders field, for maximum generalizability and standardization.

Data Management by ERT for Data Entered on the LogPad® and SitePad®

Study data entered into the LogPad® and SitePad® will be managed by ERT. ERT is a 3rd party contracted by the study funder, Janssen Research & Development, LLC. ERT specializes in clinical services and data solutions for multicenter studies. Data will enter redundant central servers hosted at two secure facilities, and will be sent to Iron Mountain for nightly back-ups and data commits to ensure data is not lost. A data archive is also created. The data is hosted on StudyWorks™, which has a user-friendly front-end for study site access to data. All data will be transferred securely to Brain-CODE at monthly intervals, as agreed in the contract with Janssen Research & Development, LLD.
Each device user, including study personnel and participants, must create a password and establish a secret question for device access. ERT verifies the integrity of data at several points, and all user actions on the LogPad and SitePad devices are captured in the data stream. The audit trail is an inherent part of the software, and ERT can track who accessed the data and when via the audit trail. Data extraction is executed by ERT. All audit trail information can be provided to each research site. Authentication is required to access the system web portal for viewing or retrieving data.

Data Management by ActiGraph®

Data recorded using the GT9X-BT Link Bluetooth Activity Monitor device by ActiGraph® will be uploaded to the CentrePoint Study Admin system (http://www.actigraphcorp.com/product-category/study-admin/), which is ActiGraph's secure cloud-based technology platform. Data will be uploaded as soon as

feasible on an ongoing basis, ideally at each in-person clinic visit. At study end, this data will be transferred to Janssen R&D LLC and OBI's Brain-CODE. The CentrePoint system is designed to preserve the integrity of data during data collection, storage, and transfer to external platforms such as Brain-CODE. Data storage is implemented using the Amazon Web Service (AWS) platform, which provides physical security, network security, availability and standards compliance. Web services and applications are deployed within the Microsoft Azure cloud platform to provide a secure framework for our public facing cloud services.

Authentication is required to access the system web portal for viewing or retrieving data. Each end-user will have a unique username and password, with specific password requirements. Passwords are encrypted on capture, not obtainable by system administrators, and transmitted via HTTPS. Data access is controlled to ensure that subject data can only be accessed by authorized users. Each end user is granted access permission to only the subject data related to their specific site(s) or study.

Voice and Speech Data

Voice data collected on the LogPad® N5 will be transferred to a third-party vendor contracted by Janssen R&D LLC. ERT will not be responsible for storing this data within StudyWorks, or including the raw voice data within the general study data transfer. ERT will create an outbound integration with the contracted third-party. Voice data from the LogPad® will be uploaded to an ERT web service. The ERT web service will be responsible for sending the data to the external vendor and creating a clinical record with StudyWorks associated with that site, subject, and report, to establish proof of transmission. At study end, all voice and speech data will be transferred to Janssen R&D LLC and OBI's Brain-CODE.

Data Management by HealthRhythms®

Participants will download the app from the Google Play Store. There are no identifiers or encrypted identifiers that will be transmitted. Only study ID and the behavioural sensed data are transmitted using Amazon Web Services (AWS) servers in the US. The mobile app requires encryption and are authenticated through AWS Cognito (via a provider such as Twitter Digits) to get valid AWS identity. Periodically (a few times a day) the app batches data together and uploads it to the HR backend system over a secure channel (HTTPS/TLS/SSL). The HR backend system is built on the Amazon Web Services (AWS) cloud. All data are stored on secure servers with secure password protection. All data received in AWS Simple Storage Service (S3) using AES-256 encryption. All iPhones are encrypted and password-protected by default, and we will explicitly require those features enabled for Android users. HealthRhythms will transfer data to Janssen periodically. All data collected and stored in the AWS servers will be deleted upon study completion.

7. Data Analysis

Statistical analysis and modeling will be carried out collaboratively between CAN-BIND and Janssen R&D, LLC. Our informatics team has wide ranging expertise spanning biostatistics, applied mathematics, machine learning, computer science, bioinformatics, neuroinformatics and high performance computing.

8. Informed Consent and Ethical Considerations

The Research Ethics Boards (REBs) of each participating site must approve the protocol and informed consent documents. The study will be conducted in accordance with the protocol. The investigators will provide the REBs with information about any changes that are made to the protocol by submitting amendments. Other ongoing information will be submitted to the REBs including information on serious or unexpected adverse events.

Good Clinical Practice (GCP)

The study will be conducted in accordance with Good Clinical Practice (GCP), as defined by the International Conference on Harmonization (ICH), and in agreement with the Declaration of Helsinki and in keeping with local regulations.

Monitoring

Monitoring of the study will be done on an ongoing basis to ensure that it is conducted in accordance with the study protocol, GCP, and applicable regulatory requirements. A UHN-based study monitor will check the accuracy of data entry into the eCRFs, as well as protocol adherence by the sites, through on-site visits and regular off-site communication with site personnel. The clinical characterization team at InDoc Research, the company that manages Brain-CODE, has expertise in eCRF creation, centralized data management, and creation of open access databases.

Also, periodic monitoring by a representative of the Janssen R&D, the clinical research partner, will occur to review the uploaded data in the different approved platforms for accuracy and completeness. Discrepancies will be resolved with the investigator or designee, as appropriate.

Delegation of Investigator Duties

The principal investigator will ensure that all persons assisting with the trial are adequately qualified, informed about the protocol, any amendments to the protocol and their trial-related duties and functions. The principal investigator will maintain a list of sub-investigators and their appropriately qualified persons to whom he/she has delegated significant trial-related duties. Should the principal investigator delegate the supervision of the study medication administration to a designated person, this individual must have the appropriate medical qualifications to effectively conduct or supervise any potential resuscitation procedures.

Participant Information and Informed Consent

Before being enrolled in the clinical study subjects must consent to participate after the nature, scope and possible consequences of the clinical study have been explained in a form understandable to them. An informed consent document that includes information about the study will be prepared and given to the participant. After reading the informed consent document, the subject must give consent in writing. The subject’s consent must be confirmed at the time consent by the personally dated signature of the subject and by the personally dated signature of the person conducting the informed consent discussions. A copy of the signed consent document must be given to the participant. The original signed consent document will be retained by each study site. The principal investigator will not undertake any measures specifically required only for the clinical study until valid consent has been obtained.

Confidentiality

Participant names will not be supplied to anyone not directly involved in the study conduct. Only the subject number will be recorded in case report forms, and if the subject name appears on any other document (e.g. laboratory report), it must be obliterated on the copy of the document retained in the Trial Master File or made available for audit. Study findings stored on a computer will be stored in accordance with local data protection laws. For the purposes of linking the Brain-CODE database to other databases, where applicable, participants' health card numbers (obtained during the main study and for participants from other CAN-BIND studies) will be entered in a secure browser, and a subject ID will be generated. De-identified data bearing this subject ID will then be transferred to OBI's secure database, known as Brain-CODE. Data will be confidential and in compliance with Ontario's privacy law (Personal Health Information Protection Act of Ontario). Unencrypted identifying information will not be sent to Brain-CODE. Personal identifying information (i.e. health card number) will be encrypted and stored in a separate database with the decryption code held by a third party that is independent of OBI and Brain-CODE and subject to security and privacy audits that is fully in compliance with Ontario's privacy law. Participant data records in Actigraph's CentrePoint and ERT's StudyWorks do not contain personally identifiable information. Each participant record has a numeric participant identifier.

9. Non-Participation and Early Termination

Every effort will be made to continue patients in the longitudinal study. Two attempts will be made to contact the patient for the follow up study. If these attempts are unsuccessful, the patient will be removed from the contact list. If a patient indicates that they do not wish to partake in the follow up study, their reason for non-participation will be documented and their name will be removed from the list of potential candidates. If a patient wishes not to continue in the follow up study, the reason for the early termination will be documented and a discontinuation visit will be requested.

10. Safety

Safety evaluations will include:

- Vital signs and weight
- Adverse event monitoring
- Concomitant medications
- Suicide assessment as measured by the Concise Health Risk Tracking (CHRT) scale
- Urine pregnancy testing for female subjects of child-bearing potential

As this is an observational study, investigators should follow the guidance in the approved local labels of medications that the subject is taking regarding discontinuation of therapy in subjects who become pregnant during the study.

Concomitant therapies must be recorded throughout the study beginning with the screening visit. Concomitant therapies should also be recorded 30 days after the end of the study only in conjunction with new or worsening adverse events or serious adverse events.

Follow up for suicidal ideation:

The CHRT scale, described above, was developed by Trivedi et al. (2011) as a tool to screen for suicidal ideation and related symptoms, as well as allowing for the prospective assessment of subjects for the

occurrence of treatment-emergent suicidality. If the person meets threshold and is evaluated as a high-risk for suicide, contact 911 or hospital emergency line and stay with the subject until assistance arrives or the person is transported to the hospital. If the person is subthreshold and is evaluated as being in a situation where there is no imminent risk, the study physician will assist in developing a plan for safety with the subject, including, but not limited to:

- Contacting participant's treating physician
- Supplying person with the appropriate referrals
- Encouraging the person to talk to a trusted family member or other community support resources
- Providing the person with suicide prevention hotline information
- If the person identifies as having no threat or risk of suicide, offer the subject resources located to their community such as community mental health services, and suicide prevention hotlines, just in case participant should find it helpful to have them on hand.

Immediately following the intervention, the responder will write up a detailed report explaining the specific symptoms such as thoughts of suicide, whether or not the person had a plan, if the person had the means to carry out the plan, the subject's history of suicide attempts, and what was done to intervene (i.e., emergency referral to ER, contact of subject's treating physician, providing information regarding community resources, etc.).

Follow up for relapse verification:

If the relapse criterion of MADRS total score ≥22 is met at a study visit (scheduled or unscheduled), an additional visit (i.e. relapse Verification visit) will be scheduled within 1 to 2 weeks to verify the relapse. Subjects who relapse will continue in the observational phase, with study visits every 8 weeks and subject will complete assessments at the frequency shown in the Time and Events Schedule until the observational follow-up is complete. Subjects who relapse but do not require a Relapse Verification visit (i.e. they meet relapse criteria other than that based on the MADRS total score) will complete the FSR-SA and have blood samples collected at their next scheduled visit. Any relapse event will be communicated by the study physician to the subject's treating physician.

11. Publications and Communications

This trial is registered at www.clinicaltrials.gov (ct.gov ID No. NCT02934334) in accordance with the policy from the International Committee of Medical Journal Editors. The results of this trial will be presented at scientific conferences and published, using the CONSORT guidelines, in peer-reviewed scientific journals.

APPENDIX 1: Time and Events Schedule (Part 1 and 2)

		Phase	Screening	Observational									
				BSL	Study Visit							Relapse Verification	End-of-Study
		Visit	1	2	3	4	5	6	7	8	X	--	--
		Week (start of)	-2	1	9	17	25	33	41	49	--	--	--
		Day	-14	1	57	113	169	225	281	337	--		
**STUDY PROCEDURES**		**DATA CAPTURE METHOD**											
**Administrative**	Informed consent		x										
	Inclusion / exclusion criteria		x										
	Demographics	REDCap	x										
	Dispense smartphone and actigraphy devices and materials			x									
	Download HR® application (if applicable)			x									
	Physical Exam	REDCap	x										x
	Medical History	REDCap	x										
	Antidepressant Treatment Tracking	REDCap	x	x									
	Reproductive History (females)	REDCap	x										
	Urine pregnancy for women of child-bearing potential		x										
	Vital Signs	REDCap	x	x	x	x	x	x	x	x	x	x	x
	CECA and LEDS interview (one-time, to be scheduled anytime)	REDCap											
**Clinician-Administered Assessments**	MINI Version 7 (if applicable)	REDCap	x										
	Montgomery Asberg Depression Rating Scale (MADRS)	SitePad and REDCap	x	x	x	x	x	x	x	x	x	x	x
	Clinical Global Impression - Severity (CGI-S)	SitePad	x	x	x	x	x	x	x	x	x	x	x
	Health Resource Risk Tracking (HRUQ)	SitePad	x	x	x	x	x	x	x	x	x	x	x
	Concise Health Risk Tracking (CHRT-Baseline) Clinician Module	SitePad	x	x	x	x	x	x	x	x	x	x	x
	Concise Health Risk Tracking (CHRT- Since Last Visit) Clinician Module	SitePad		x	x	x	x	x	x	x	x	x	x
**Patient-Administered Assessments**	Early Trauma Inventory Self-Report Form (ETISR-SF)	SitePad	x										
	Recent Life Changes Stress Test (RLCST)	SitePad	x										
	Recent Life Changes Stress Test (RCLST) - Since Last Visit	SitePad		x	x	x	x	x	x	x	x	x	x
	Concise Health Risk Tracking (CHRT-Baseline) Subject Module	SitePad	x										
	Concise Health Risk Tracking (CHRT- Since Last Visit)	SitePad		x	x	x	x	x	x	x	x	x	x
	Snaith-Hamilton Pleasure Scale (SHAPS)	SitePad		x	x	x	x	x	x	x	x		x
	Pain-Frequency, Intensity, Burden Scale (P-FIBS)	SitePad		x	x	x	x	x	x	x	x		x
	Depression Implicit Association Test (DIAT)	SitePad		x	x	x	x	x	x	x	x		x
	First Symptom of Relapse - Self Assessment (FSR-SA)	SitePad			x	x	x	x	x	x	x	x	x

		Phase	Screening	Observational									
				BSL	Study Visit							Relapse Verification	End-of-Study
		Visit	1	2	3	4	5	6	7	8	X	--	--
		Week (start of)	-2	1	9	17	25	33	41	49	--	--	--
		Day	-14	1	57	113	169	225	281	337	--		
Patient-Administered Assessments	Pittsburgh Sleep Quality Index (PSQI)	REDCap		x	x	x	x	x	x	x	x	x	x
	Sheehan Disability Scale (SDS)	REDCap		x	x	x	x	x	x	x	x	x	x
	Quality of Life Enjoyment and Satisfaction Questionnaire (Q-LESQ)	REDCap		x	x	x	x	x	x	x	x	x	x
	Brief Symptom Inventory (BSI-53)	REDCap		x	x	x	x	x	x	x	x	x	x
	Lam Employment Absence and Productivity Scale (LEAPS)	REDCap		x	x	x	x	x	x	x	x	x	x
	Brief Diet Inventory (BDI)	REDCap		x	x	x	x	x	x	x	x	x	x
	International Physical Activity Questionnaire (IPAQ)	REDCap		x	x	x	x	x	x	x	x	x	x
	World Health Organization Quality of Life - Short Version	REDCap		x	x	x	x	x	x	x	x	x	x
	Biological Rhythm Interview Assessment	REDCap		x	x	x	x	x	x	x	x	x	x
	Rothschild Scale for Antidepressants Tachyphlaxic (R-SAT)	REDCap		x	x	x	x	x	x	x	x	x	x
	Quick Inventory of Depression Symptology-Self	LogPad		x	x	x	x	x	x	x	x	x	x
	Generalized Anxiety Disorder Scale (GAD-7)	LogPad		x	x	x	x	x	x	x	x	x	x
	Medical Outcomes Study Sleep Scale, Revised (MOS Sleep-R)	LogPad		x	x	x	x	x	x	x	x		x
	Patient Adherence to Antidepressant Medication Questionnaire	LogPad		x	x	x	x	x	x	x	x	x	x
	Perceived Stress Scale (PSS)	LogPad		x	x	x	x	x	x	x	x	x	x
	WHODAS 2.0 (Disability Assessment Schedule)	LogPad		x	x	x	x	x	x	x	x		x
	EuroQoL Group, 5-Dimension, 5-Level (EQ-5D-5L)	LogPad		x	x	x	x	x	x	x	x		x
	Speech and Voice Characteristics	LogPad		x	x	x	x	x	x	x	x	x	x
	Photographic Affect Meter (PAM)	HealthRhythms App		x	x	x	x	x	x	x	x	x	x
	Patient Health Questionnaire (PHQ-8)	HealthRhythms App		x	x	x	x	x	x	x	x	x	x
	Sleep Questionnaire	HealthRhythms App		x	x	x	x	x	x	x	x	x	x
Others	Concomitant Therapy			x	x	x	x	x	x	x	x	x	x
	Adverse Events			x	x	x	x	x	x	x	x	x	x
	Biospecimen Collection			x	x	x	x	x	x	x	x	x	x

- If feasible, the screening and baseline visits can occur on the same day. Duplicate assessments will not be required if the screening and baseline visits occur on the same day.
- The observational phase will be of variable duration, with study visits every 8 weeks until a number of relapse has been observed as per interim analysis, or until the end of the 1-year enrolment period for the last-subject-in, whichever comes first.
- If relapse criterion of MADRS total score $\geq$22 is met at a study visit, an additional visit (Relapse Verification Visit) will be scheduled within 1 to 2 weeks to verify relapse. Subjects who relapse will continue in the observational phase, with study visits every 8 weeks as shown in the table.
- Subjects will continue to have study visits every 8 weeks after Week 49. Visit numbers will continue sequentially (e.g. Visit 9, 10, 11, etc.).
- Patient-administered assessments collected using the LogPad will be recorded weekly, in between clinic visits; and weekly for PAM and Sleep Questionnaire and then bi-weekly for the PHQ-8 which is collected using the HealthRhythms® application.

Appendix 2: List of Eligible Devices

The minimum Android eligible to download the HealthRhythms® app is Android 5+.

And for the iOS it is iPhone5s.

Support is offered for the following specific android devices:

Samsung Galaxy S7
Samsung Galaxy S7 Edge
Samsung Galaxy S6
Samsung Galaxy S6 Edge
Samsung Galaxy S5
Samsung Galaxy Note 5
Samsung Galaxy Note 4
Samsung Galaxy J7
LG G5
LG G4

References:

1. Abdullah, S., Matthews, M., Frank, E., Doherty, G., Gay, G. and Choudury, T. Automatic Detection of Social Rhythms in Bipolar Disorder. Journal of the American Medical Informatics Association, *in press.*
2. Bifulco, A., G.W. Brown, and T.O. Harris, *Childhood Experience of Care and Abuse (CECA): a retrospective interview measure.* J Child Psychol Psychiatry, 1994. 35(8): p. 1419-35.
3. Bifulco, A., Brown, G., Edwards, A., Harris, T., Neilson, E., Richards, C., and Robinson, R. *Life Events and Difficulties Schedule (LEDS–2): Vol. 1. Life events manual.* 1989. London, England: Royal Holloway and Bedford New College, University of London.
4. Bremner JD, Bolus R, Mayer EA. Psychometric properties of the Early Trauma Inventory-Self Report. J Nerv Ment Dis. 2007;194:211-218.
5. Brooks R, with the EuroQol Group. EuroQol: the current state of play. Health Policy. 1996;37:53-72.
6. Buysse DJ, Reynolds CF 3rd, Monk TH, Berman SR, Kupfer DJ. *The Pittsburgh Sleep Quality Index: a new instrument for psychiatric practice and research.* Psychiatry Res, 1989. 28(2):193-213.29.
7. Carlozzi NE, Kratz AL, Downing NR, Goodnight S, Miner JA, Migliore N, Paulsen JS. Validity of the 12-item World Health Organization Disability Assessment Schedule 2.0 (WHODAS 2.0) in individuals with Huntington disease (HD). Qual Life Res. 2015 Aug;24(8):1963-71.
8. Cohen S, Kamarck T, Mermelstein R. A global measure of perceived stress. J Health Soc Behav. 1983;24(4):385-396.
9. Craig CL, Marshall AL, Sjöström M, Bauman AE, Booth ML, Ainsworth BE, Pratt M, Ekelund U, Yngve A, Sallis JF, Oja P. *International physical activity questionnaire: 12-country reliability and validity. Med Sci Sports Exerc,* 2003. 35(8):1381-95. 31.
10. dela Cruz AM, Bernstein IH, Greer TL, et al. Self-rated measure of pain frequency, intensity, and burden: psychometric properties of a new instrument for the assessment of pain. J Psychiatr Res., 2014 Dec; 9:155-60.
11. Derogatis LR, Melisaratos N. The Brief Symptom Inventory: an introductory report. Psychol Med., 1983 Aug;13(3):595-605.
12. Endicott J, Nee J, Harrison W, Blumenthal R. Quality of Life Enjoyment and Satisfaction Questionnaire: a new measure. Psychopharmacology bulletin. 1993;29:321–6.
13. Free C, Phillips G, Watson L, Galli L, Felix L, Edwards P, Patel V, Haines A. The effectiveness of mobile-health technologies to improve health care service delivery processes: a systematic review and meta-analysis. PLoS Med. 2013;10(1):e1001363.
14. Giglio, L.M.F., et al., Development and use of a biological rhythm interview. J Affect Disord, 2009. .118(1-3): p. pp.
15. Granholm E, Ben-Zeev D, Link PC, Bradshaw KR, Holden JL. Mobile Assessment and Treatment for Schizophrenia (MATS): a pilot trial of an interactive text-messaging intervention for medication adherence, socialization, and auditory hallucinations. Schizophr Bull. 2012;38(3):414-25.
16. Guy W. ECDEU Assessment Manual for Psychopharmacology. Rockville, MD: U.S. Department of Health, Education, and Welfare; 1976.
17. Hays RD, Martin SA, Sesti AM, Spritzer KL. Psychometric properties of the Medical Outcomes Study Sleep measure. Sleep Med. 2005 Jan;6(1):41-4.
18. Judd LL, Akiskal HS, Maser JD, Zeller PJ, Endicott J, Coryell W, et al. A prospective 12-year study of subsyndromal and syndromal depressive symptoms in unipolar major depressive disorders. Archives of general psychiatry. 1998;55(8):694–700.

19. Kennedy SH, Downar J, Evans KR, Feilotter H, Lam RW, Glenda M, et al. The Canadian Biomarker Integration Network in Depression (CAN-BIND): Advances in Response Prediction. Current Pharmaceutical Design. 2012;18(416):1–12.
20. Kessler RC, Berglund P, Demler O, Jin R, Koretz D, Merikangas KR, et al. The epidemiology of major depressive disorder: results from the National Comorbidity Survey Replication (NCS-R). JAMA : the journal of the American Medical Association. 2003 Jun 18;289(23):3095–105.
21. Kroenke K., Strine T.W., Spritzer R.L., Williams J.B., Berry J.T., Mokdad A.H. The PHQ-8 as a measure of current depression in the general population. J Affect Disord. 2009; 114(1-3):163-73.
22. Lam, R.W., E.E. Michalak, and L.N. Yatham, *A new clinical rating scale for work absence and productivity: validation in patients with major depressive disorder.* BMC Psychiatry, 2009. 9: p. 78.
23. Meites TM, Deveney CM, Steele KT, Holmes AJ, Pizzagalli DA. Implicit depression and hopelessness in remitted depressed individuals. Behav Res Ther. 2008;46:1078-1084.
24. Miller MA, Rahe RH. Life changes scaling for the 1990s. J Psychomat Res. 1997;43(3):279-292.
25. Montgomery SA, Asberg M. A new depression scale designed to be sensitive to change. British Journal of Psychiatry. 1979;134:382–9.
26. Mueller TI, Leon AC, Keller MB, Solomon DA. Recurrence after recovery from major depressive disorder during 15 years of observational follow-up. American Journal of Psychiatry. 1999;156(7):1000–6.
27. Reid SC, Kauer SD, Hearps SJ, Crooke AH, Khor AS, Sanci LA, Patton GC. A mobile phone application for the assessment and management of youth mental health problems in primary care: health service outcomes from a randomised controlled trial of mobiletype. BMC Fam Pract. 2013;14:84.
28. Rush, A.J., et al., *The 16-Item Quick Inventory of Depressive Symptomatology (QIDS), clinician rating (QIDS-C), and self-report (QIDS-SR): a psychometric evaluation in patients with chronic major depression.* Biol Psychiatry, 2003. 54(5): p. 573-83.
29. Sheehan D. V., et. al. *The Mini-International Neuropsychiatric Interview (M.I.N.I): the development and validation of a structured diagnostic psychiatric interview for DSM-V and ICD-10.* J Clin Psychiatry, 1998;**59(suppl 20)**:22-33.
30. Sheehan D V, Harnett-Sheehan K, Raj B a. The measurement of disability. International clinical psychopharmacology. 1996;11 Suppl 3:89–95.
31. Snaith RP, Hamilton M, Morley S, Humayan A, Hargreaves D, Trigwell P. *A scale for the assessment of hedonic tone the Snaith-Hamilton Pleasure Scale.* Br J Psychiatry, 1995. 167(1): p. 99-103.
32. Spitzer, R.L., et al., A brief measure for assessing generalized anxiety disorder: the GAD-7. Arch Intern Med, 2006. 166(10): p. 1092-7.
33. Trivedi MH, Wisniewski SR, Morris DW, et al. Concise Health Risk Tracking scale: a brief self-report and clinician rating of suicidal risk. J Clin Psychiatry. 2011;72:757-764.
34. Uher RG. Common genetic variation and antidepressant efficacy in major depressive disorder: a meta-analysis of three genome-wide pharmacogenetic studies. The American journal of psychiatry. 2013 Feb 1;170(2):207–17.
35. The WHOQOL Group. The development of the World Health Organization quality of life assessment instrument (the WHOQOL). In J. Orley and W. Kuyken (Eds) Quality of Life Assessment: International Perspectives. Heidelberg: Springer Verlag, 1994.
