# Supplementary material for: Personalized relapse prediction in patients with major depressive disorder using digital biomarkers
Source: Sci Rep. 2023 Oct 30;13:18596. doi: 10.1038/s41598-023-44592-8 (PMC10616277; doi:10.1038/s41598-023-44592-8)
Supplement: Supplementary file 3 — Supplementary Information 3. [file 41598_2023_44592_MOESM3_ESM.docx]

**SUPPLEMENT-3**

**Personalized Relapse Prediction in Patients with Major Depressive Disorder using Digital Biomarkers**

Srinivasan Vairavan*, PhD, Homa Rashidisabet, BS, Qingqin S. Li, PhD, Seth Ness, PhD, Randall L. Morrison, MD, Claudio N. Soares, MD, PhD, FRCPC, MBA, Rudolf Uher, MD, PhD, CCT, Benicio N. Frey, MD, MSc, PhD, Raymond W. Lam, MD, FRCPC, FCAHS, Sidney H. Kennedy, MD, FRCPC, MRC Psych, Madhukar Trivedi, MD, Wayne C. Drevets, MD, Vaibhav A. Narayan, PhD

*Corresponding author

Srinivasan Vairavan, PhD

Janssen Research & Development, LLC

1125 Trenton Harbourton Road, Titusville, NJ 08560, USA

Email: [svairava@its.jnj.com](mailto:svairava@its.jnj.com), Tel: +1 609 7307592

**Contents**

[**I.** **Tables: Distribution of relapse criteria and EncDec parameters** 3](#_Toc129810093)

[**Table S1.** Distribution of relapse criteria in the analyzed participants 3](#_Toc129810094)

[**Table S2.** LSTM EncDec parameters 4](#_Toc129810095)

[**II.** **Figures: Study Design and LSTM EncDec-AD architecture** 5](#_Toc129810096)

[**Fig. S1** Study design 5](#_Toc129810097)

[**Fig. S2** Schematics of the LSTM EncDec-AD architecture 5](#_Toc129810098)

[**III.** **Adaptive anomalous instances detection using dynamic thresholds** 7](#_Toc129810099)

[(i) Adaptive anomaly threshold determination: 7](#_Toc129810100)

[(ii) Prune Anomalies: 7](#_Toc129810101)

[(iii) Figures 3 and 4: 8](#_Toc129810102)

[**Fig. S3** Flowchart for adaptive anomolous instance determination using dynamic thresholds. 8](#_Toc129810103)

[**Fig. S4** Adaptive anomaly detection using dynamic thresholds. 9](#_Toc129810104)

[**IV.** **Figures: Continuous evaluation and distribution of evaluated visits** 10](#_Toc129810105)

[**Fig. S5** Continuous evaluation of the personalized relapse prediction model 10](#_Toc129810106)

[**Fig. S6** Distribution of evaluated visits in (a) relapse and (b) non-relapse patients 11](#_Toc129810107)

[**V.** **Interpretation of actigraphy features and risk factors for relapse** 12](#_Toc129810108)

[***Relationship of actigraphy features with depression clinical outcome (relapse and symptom severity) and self-reported core-symptoms of depression:*** 12](#_Toc129810109)

[(i) Longitudinal modeling of actigraphy features: 12](#_Toc129810110)

[(ii) Inter-individual variability in actigraphy features and their association with core-symptoms of depression: 13](#_Toc129810111)

[**Fig. S7** AME for each depression clinical outcome (relapse and symptom severity) measured bi-monthly 15](#_Toc129810112)

[**Fig. S8** AME for self-reported symptom severity measured weekly (VQIDS-SR5) 16](#_Toc129810114)

[**Fig. S9** Actigraphy features correlation with VQIDS-SR5 17](#_Toc129810116)

[**VI.** **Personalized relapse prediction framework’s performance across gender and age groups** 18](#_Toc129810117)

[**Table S3.** Relapse prediction framework performance on unseen test data, provider burden, and patient burden for the age range 18-44 years 19](#_Toc129810118)

[**Table S4.** Relapse prediction framework performance on unseen test data, provider burden, and patient burden for the age range 45-64 years 20](#_Toc129810119)

[**Table S5.** Relapse prediction framework performance on unseen test data, provider burden, and patient burden for male patients 21](#_Toc129810120)

[**Table S6.** Relapse prediction framework performance on unseen test data, provider burden, and patient burden for female patients 22](#_Toc129810121)

[**VII. References** 23](#_Toc129810122)

1. **Tables: Distribution of relapse criteria and EncDec parameters**

## **Table S1.** Distribution of relapse criteria in the analyzed participants

| Relapse criteria | N | |
| --- | --- | --- |
|  | **OBSERVEMDD** | **CBN-WELL** |
| MADRS [1] total score of 22 at a study visit and a symptom worsening confirmed over an approximately 1 to 2-week interval. | 45 | 8 |
| Hospitalization for worsening of depression | 2 | 0 |
| Suicidal ideation with intent, or suicidal behavior | 1 | 2 |
| Investigator’s decision | 2 | 0 |

EncDec, Encoder-Decoder scheme; MADRS, Montgomery Asberg Depression Rating Scale

## **Table S2.** LSTM EncDec parameters

| Parameters | Values |
| --- | --- |
| Hidden layer | 1 |
| Number of hidden units | 14 |
| Sequence length $\boldsymbol{(l)}$ | 14 |
| Training iterations | 100 |
| Learning rate | 0.001 |
| Batch size | 1 |
| Optimizer | Adam |
| Number of input features | 7 |

EncDec, Encoder-Decoder scheme; LSTM, Long Short-Term Memory Networks

# **Figures: Study Design and LSTM EncDec-AD architecture**

## **Fig. S1** Study design


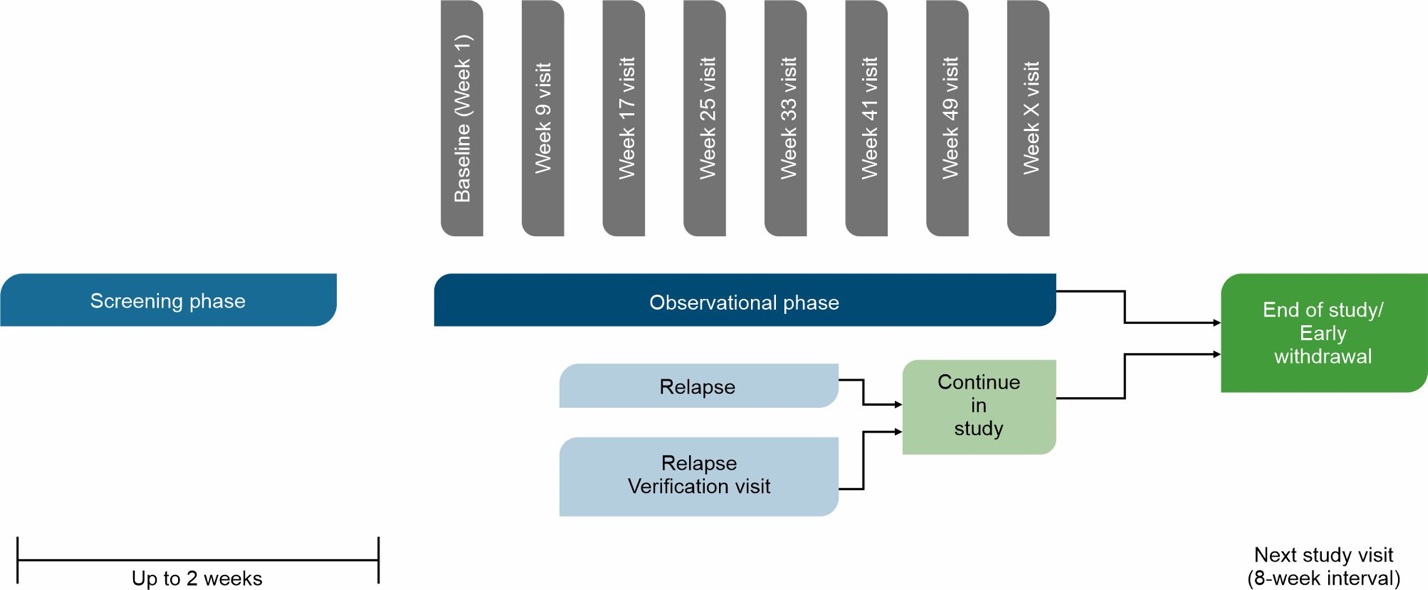


## **Fig. S2** Schematics of the LSTM EncDec-AD architecture


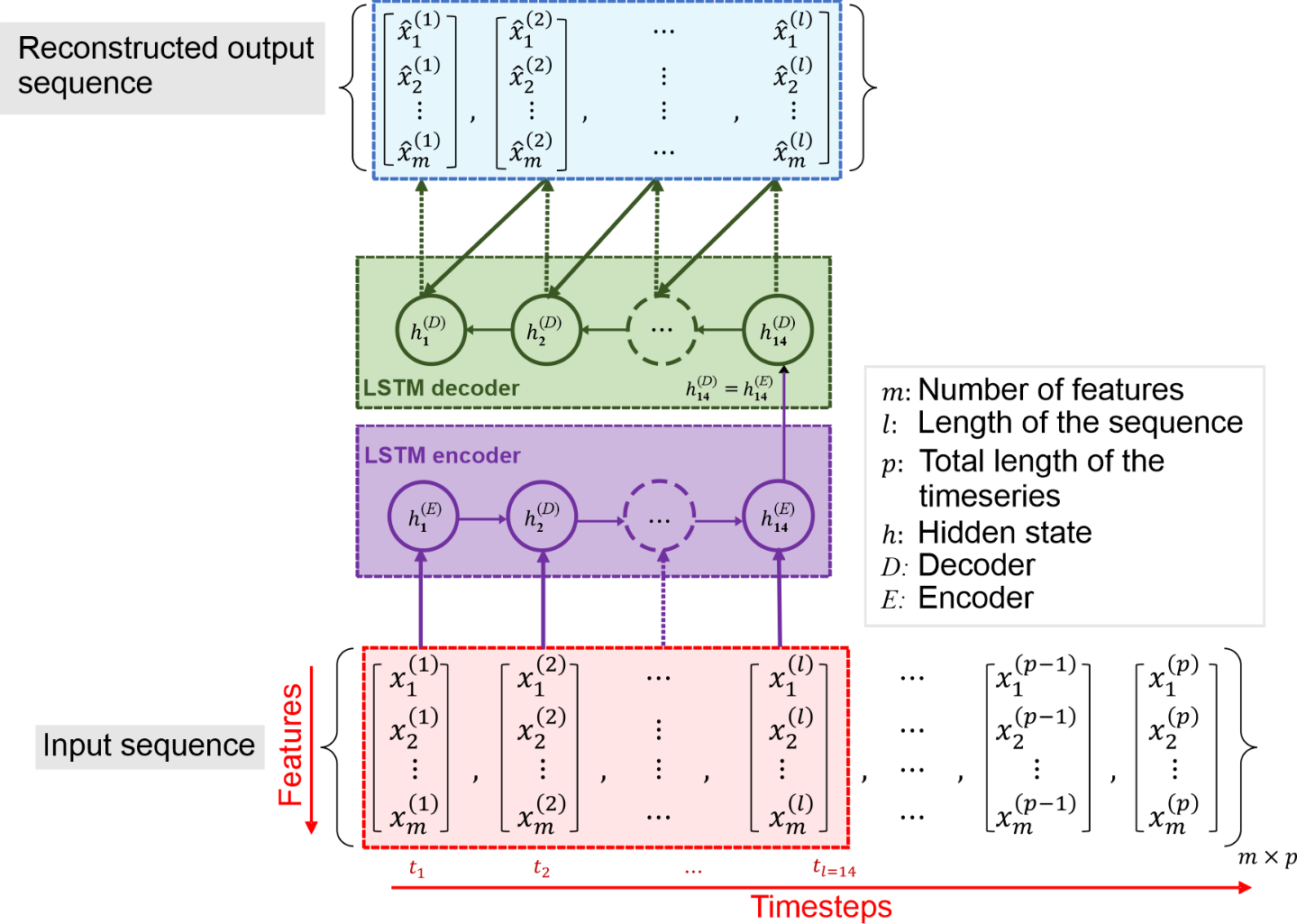


EncDec-AD, encoder-decoder anomaly detection; LSTM, Long Short-Term Memory Networks.

# **Adaptive anomalous instances detection using dynamic thresholds**

The flowchart for the different steps involved in the procedure shown in **Fig. S3** and **Fig. S4** depicts it for a representative patient. The two main steps involved in anomalous instance detection were the determination of adaptive anomaly threshold and the pruning of identified anomalous instances. They are both described below and shown in **Fig. S3b** and **Fig. S3c** respectively.

1. Adaptive anomaly threshold determination:

An anomaly threshold $(\epsilon_{all})$, defined as $\epsilon_{all}= \mu\left( a_{all} \right)+z\sigma\left( a_{all} \right)$; where $a_{all}$ is the anomaly score time series and $z$ is set to two representing the number of SDs above $\mu\left( a_{all} \right)$. The anomalous instances and the corresponding sequence of anomalous instances (defined as 1 time point before and after each anomalous instance) in $a_{all}$ above $\epsilon_{all}$ were removed, resulting in a time series of non-anomalous sections of $a_{all}$, namely, $a_{all\_non\_anom}$ (**Fig. S4b**). The anomalous instances in $a_{14}$ were identified using the procedure outlined in Hundman et al. [2]. The anomaly thresholds ($\epsilon_{all\_non\_anom})$were computed using $a_{all\_non\_anom}$ and similar values for $z$ as reported in Hundman et al. [2] were used. The rationale behind the choice of $a_{all\_non\_anom}$ for threshold calculation is to mitigate scenarios of huge peaks present in historical anomaly score $a_{all}$ leading to missed detection of anomalous instances in $a_{14}$. All the steps are shown in **Fig. S3b** **and Fig. S4d**.

1. Prune Anomalies:

Once the anomalous instances and their corresponding sequences were identified in $a_{14}$, the detected anomalous instances were evaluated to check if an instance is substantially above the background or if it is present in a segment with more anomalous instances. All the steps involved in the pruning process are shown in **Fig. S3c** and **Fig. S4f**.

1. Figures 3 and 4:

**Fig. S3** Flowchart for adaptive anomalous instance determination using dynamic thresholds.

(a) Overview of the different steps involved in the adaptive anomalous instances determination. All the steps are repeated with $T= 14 to N$ and $p= 0 to \left( N-14 \right)$ in steps of 1 day where $N$ is the total number of available anomaly scores; (b) Steps involved in adaptive anomaly threshold determination; (c) Steps involved in pruning identified anomalous instances. The threshold ($\rho$) is set to 30% above the background.


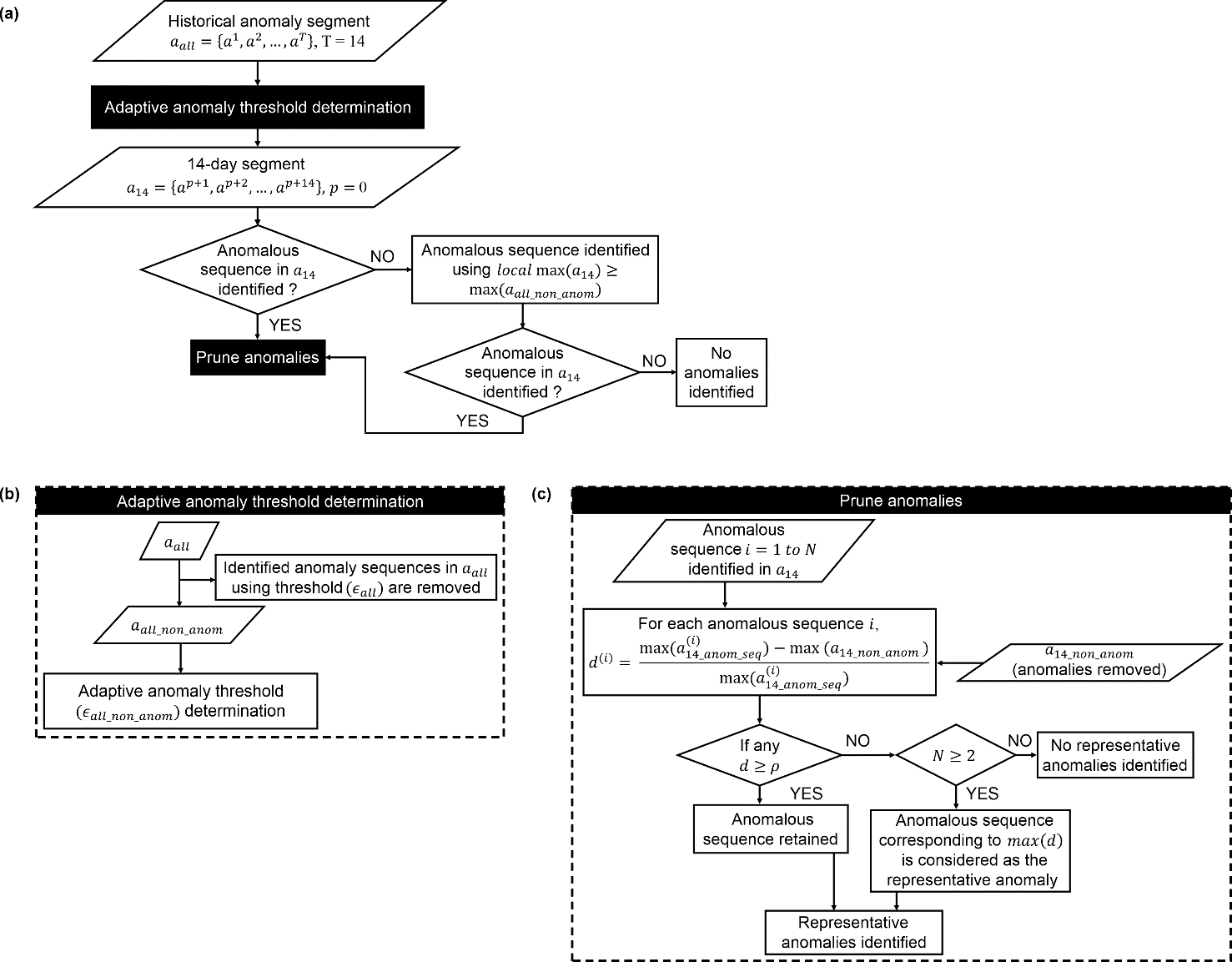


**Fig. S4** Adaptive anomaly detection using dynamic thresholds.

(a) Representative anomaly scores corresponding to test data segment including $a_{all}$ and $a_{14}$; (b) $a_{all\_non\_anom}$ is generated from $a_{all}$ by removing anomalous sequence above the threshold $\epsilon_{all}$ as shown in (a); (c) Anomaly scores corresponding to $a_{14}$; (d) Identification of potential anomalous sequence in $a_{14}$ that are above anomaly threshold $\epsilon_{all\_non\_anom}$; (e) $a_{14\_non\_anom}$ is generated from $a_{14}$ by removing anomlous sequence above the threshold $\epsilon_{all\_non\_anom}$ as shown in (d); (f) The identified anomalous instances in (d) are further pruned based on their normalized difference ($d$) from ${max(a}_{14\_non\_anom})$ and the anomalous instance with d $\geq$ $\rho$ where $\rho$ = 0.3 is chosen as the representative anomalous instance in $a_{14}$.


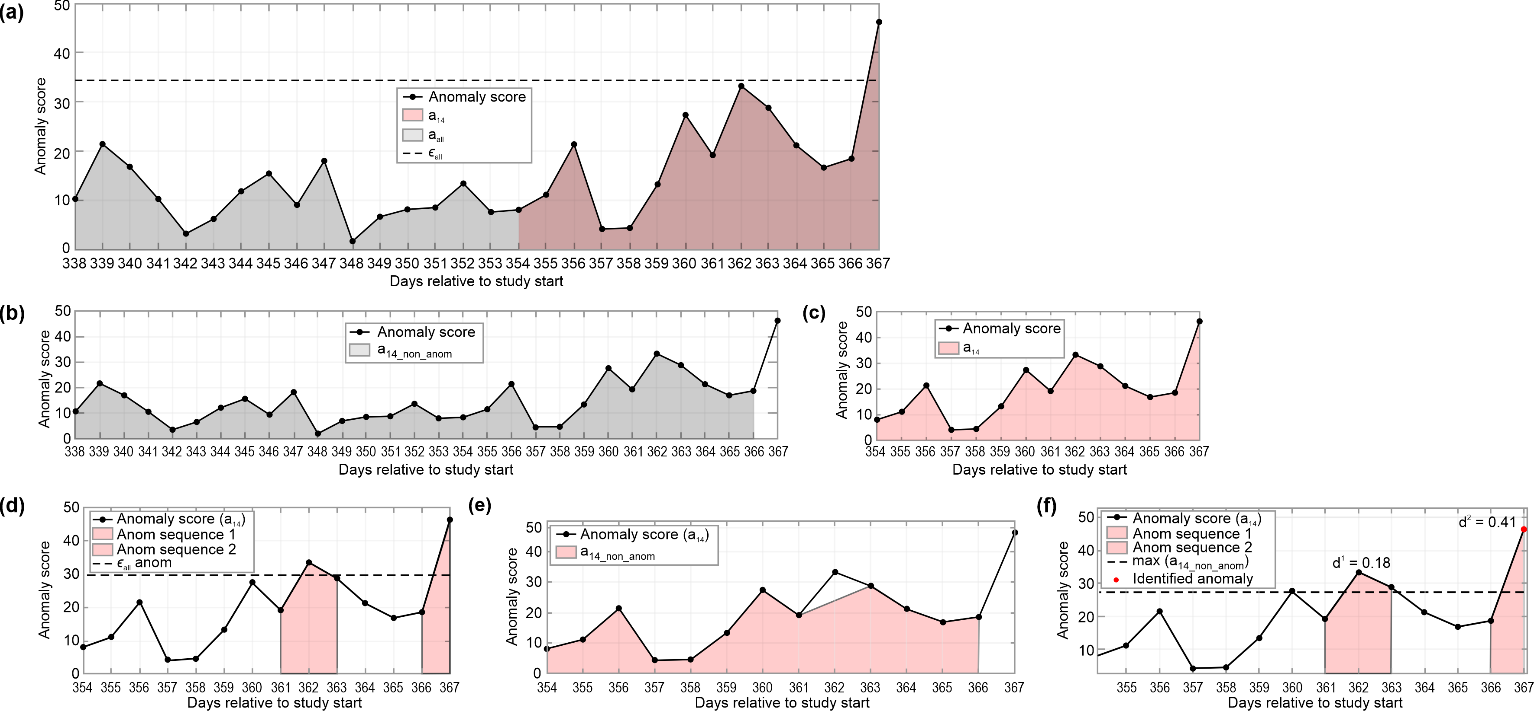


EncDec-AD, encoder-decoder anomaly detection; GAD-7, Generalized Anxiety Disorder 7-item scale; MADRS, Montgomery Asberg Depression Rating Scale; VQIDS-SR5, the Very Quick Inventory of Depressive Symptomatology

# **Figures: Continuous evaluation and distribution of evaluated visits**

## **Fig. S5** Continuous evaluation of the personalized relapse prediction model

**
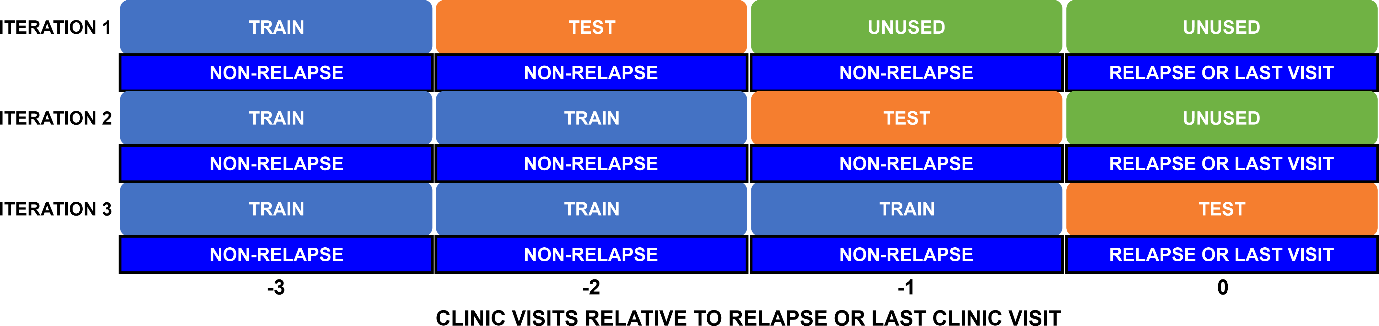
**

## **Fig. S6** Distribution of evaluated visits in (a) relapse and (b) non-relapse patients

**
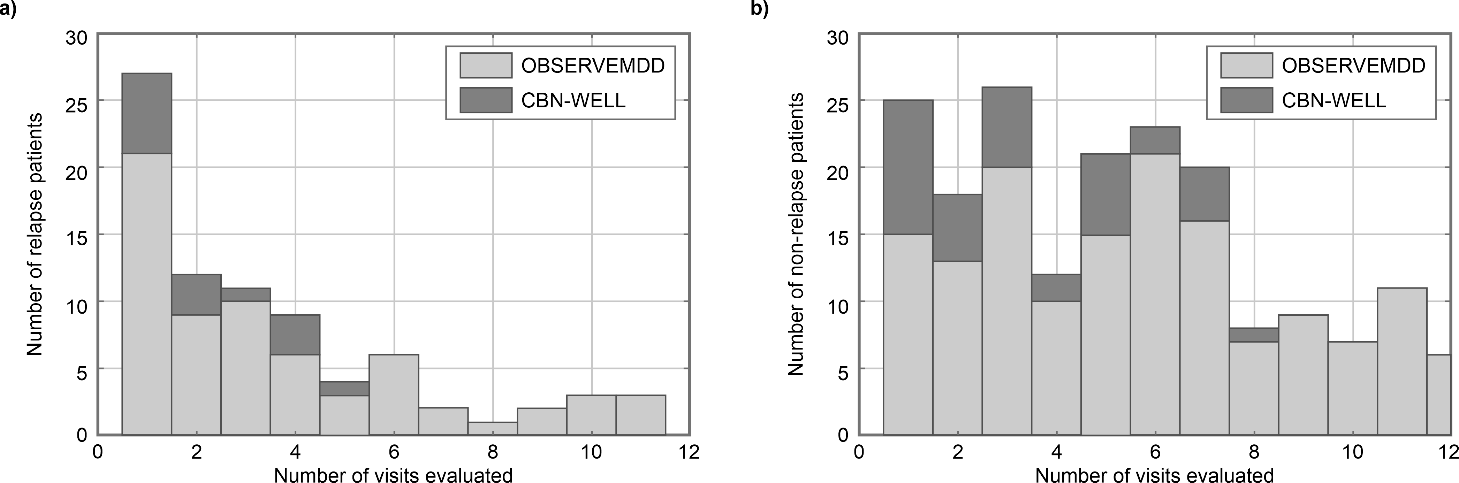
**

1. **Interpretation of actigraphy features and risk factors for relapse**

We extracted fractal and entropy features from actigraphy data to quantify fractal motor activity regulation [3], circadian regulation [3], and entropic signatures [4].

## ***Relationship of actigraphy features with depression clinical outcome (relapse and symptom severity) and self-reported core-symptoms of depression:***

To quantify the clinical relevance of actigraphy features and risk factors of relapse, we performed longitudinal modeling of actigraphy features with depression clinical outcomes (relapse, symptom severity with MADRS) and self-reported core symptoms of depression using VQIDS-SR5. In addition, we also computed the Spearman correlation for each patient actigraphy data with VQIDS-SR5 to quantify the inherent inter-individual variability.

1. Longitudinal modeling of actigraphy features: We assessed the association between actigraphy features and depression clinical outcome (relapse and symptom severity with MADRS) using linear mixed effect models (LMEs) (logistic regression models for relapse and linear models for symptom severity) in both studies. We included an individual random intercept to account for repeated assessments within each participant. Each actigraphy feature was standardized (mean = 0; standard deviation = 1) to improve estimation and interpretability. Age and gender are included as covariates as these influence activity patterns [5].

The effect of actigraphy features on depression clinical outcome was quantified using average marginal effects (AME). A marginal effect is the partial derivative of the regression equation with respect to each variable in the model for each unit in the data and the AME is the mean of these partial derivatives. For relapse, the AME represents the percentage points increase in the probability of relapse per standard deviation difference in the actigraphy feature. For depression severity, the AME represents the increase in MADRS total score per standard deviation difference in the actigraphy feature.

Models were estimated using the lme4 [6] and marginal effects [7] package for R. For each of 1410 (OBSERVEMDD: 1254 and CBN-WELL: 156) bi-monthly visit across 277 MDD patients available in both studies, we extracted the median and variance of available actigraphy features and associated with relapse and symptom severity measured with MADRS. In addition, we also assessed the association between weekly self-reported VQIDS-SR5 (N = 277 patients, median 19 weekly visits [interquartile range: 7-36 weekly visits]) corresponding to core-symptoms of depression and actigraphy features extracted over the preceding week in both studies. We also associated actigraphy features with QIDS-SR16 and we did find the same directionality in the associations.

Among the significant associations as observed from **Fig. S7**, a one standard deviation increase in ‘median of sample entropy during afternoon’ was associated with 0.99% (2.1 – 0.12) decrease in the probability of relapse and 0.68 (1.12 – 0.23) unit decrease in depression severity (**Fig. S7** a-c). In addition, a one standard deviation increase in ‘variance of sample entropy during afternoon’ was associated with 2.29 % (4.89 – 0.31) decrease in the probability of relapse and 0.35 (0.67 – 0.03) unit decrease in depression severity (**Fig. S7** b-d).

The AME analysis of the self-reported symptom severity (VQIDS-SR5) (**Fig. S8**) showed similar trends with actigraphy features as observed with AME analysis for depression clinical outcome. A one standard deviation increase in sample entropy during morning, afternoon, evening and DFA $\alpha_{1}$ was associated with 0.07 unit decrease in depression severity.

A reduced DFA $\alpha_{1}$ has been associated with dysregulation in higher brain activities including mood and cognitive function [3]. In addition, sample entropy during morning, afternoon, and evening time periods also decreases with increasing depression severity reflecting a reduced complexity in the underlying actigraphy timeseries.

These findings highlight that with increasing levels of depression, activity patterns become more monotonous (less variable) leading to decreased fractal regulation and entropy features.

1. Inter-individual variability in actigraphy features and their association with core-symptoms of depression:

To quantify the inter-individual variability in actigraphy features and their association with core-symptoms of depression, we computed the Spearman correlation between actigraphy features and VQIDS-SR5 collected every week for each patient. We restricted the analysis to patients with $\geq$12 weekly assessments in both the studies (N = 180 patients, median 30 weekly visits (interquartile range: 19-46 weekly visits)) to ensure reliability in estimation of correlation. The correlation matrix is shown in **Fig. S9**. As observed from the correlation plot there is inherent intra and inter-individual variability in the association of actigraphy features with core-symptoms of depression which necessitates personalized (N-of-1) models.

Even though the actigraphy features that we utilized in our N-of-1 personalized framework have clinical relevance (**Figs. S7-S8**), since the framework trains on prior data of an individual, *our framework does not rely on the same features to generalize across all individuals rather it looks at multivariate anomaly across the features on an individual basis*. We see that as a particular strength of this approach. Through this framework, the generalizability across individuals and different populations has been achieved by learning multivariate anomaly patterns pertaining to significant features for each individual, which need not be identical, but they do share commonalities and are associated with clinical symptoms in varying ways across individuals as shown in **Figs. S7-S9**.

**Fig. S7** AME for each depression clinical outcome (relapse and symptom severity) measured bi-monthly


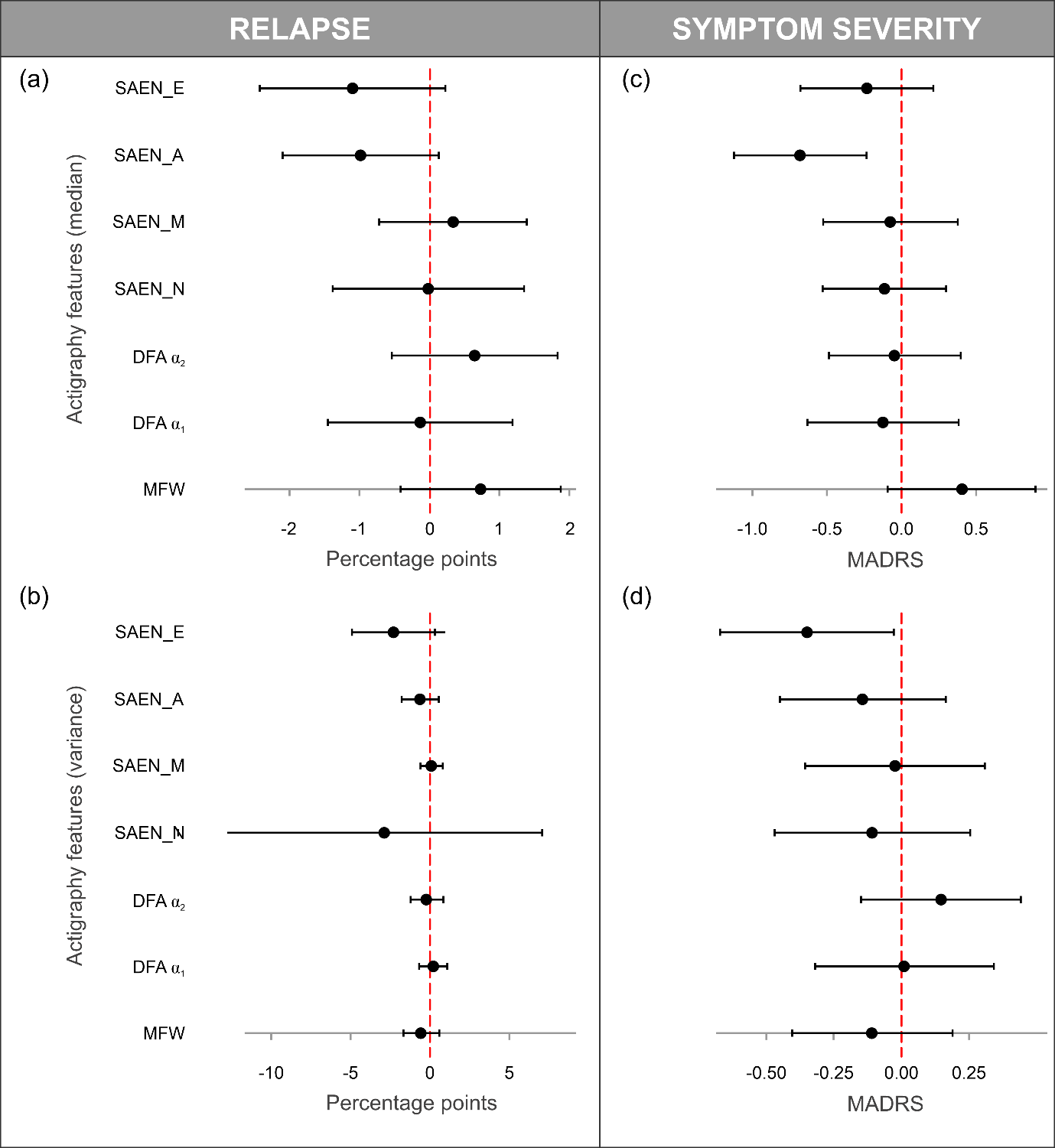


MFW corresponds to multifractal spectrum width, DFA $\alpha_{1}$ corresponds to the fluctuation exponent computed from 10 minutes (i.e., 10 data points with epoch length of 1 min) to 90 minutes, DFA $\alpha_{2}$ corresponds to the fluctuation exponent computed during 120 minutes to 600 minutes, respectively to capture the distinct regions of activity dynamics. SAEN_N corresponds to Sample Entropy during night (12 AM to 6 AM), SAEN_M corresponds to Sample entropy during morning (6 AM to 12 PM), SAEN_A corresponds to Sample entropy during afternoon (12 PM to 6 PM) and SAEN_E corresponds to Sample entropy during evening (6 PM to 12 AM).

**Fig. S8** AME for self-reported symptom severity measured weekly (VQIDS-SR5)


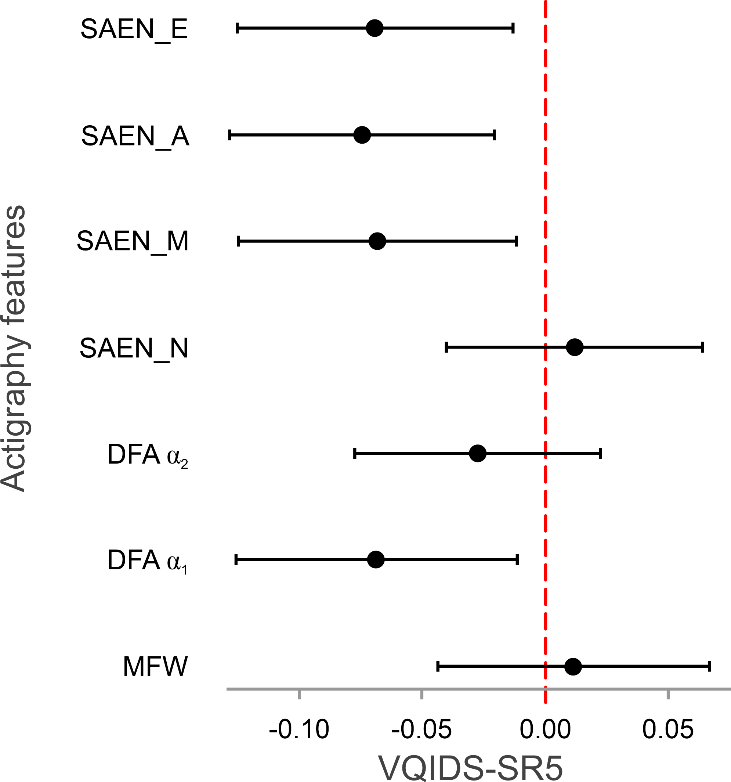


MFW corresponds to multifractal spectrum width, DFA $\alpha_{1}$ corresponds to the fluctuation exponent computed from 10 minutes (i.e., 10 data points with epoch length of 1 min) to 90 minutes, DFA $\alpha_{2}$corresponds to the fluctuation exponent computed during 120 minutes to 600 minutes, respectively to capture the distinct regions of activity dynamics. SAEN_N corresponds to Sample Entropy during night (12 AM to 6 AM), SAEN_M corresponds to Sample entropy during morning (6 AM to 12 PM), SAEN_A corresponds to Sample entropy during afternoon (12 PM to 6 PM) and SAEN_E corresponds to Sample entropy during evening (6 PM to 12 AM).

**Fig. S9** Actigraphy features correlation with VQIDS-SR5

**
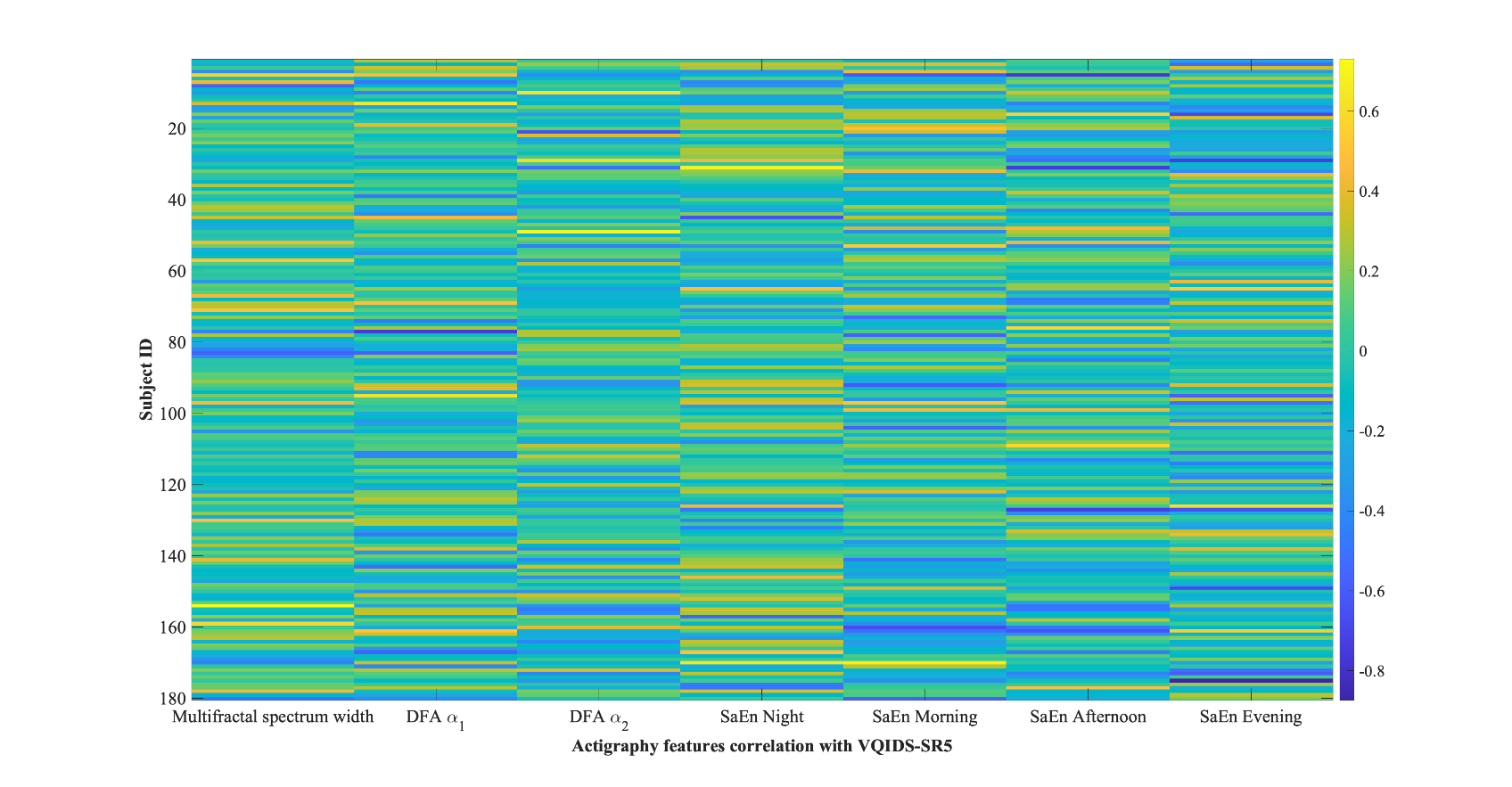
**

DFA $\alpha_{1}$ corresponds to the fluctuation exponent computed from 10 minutes (i.e., 10 data points with epoch length of 1 min) to 90 minutes, DFA $\alpha_{2}$corresponds to the fluctuation exponent computed during 120 minutes to 600 minutes, respectively to capture the distinct regions of activity dynamics. SaEn Night corresponds to Sample Entropy during night (12 AM to 6 AM), SaEn Morning corresponds to Sample entropy during morning (6 AM to 12 PM), SaEn Afternoon corresponds to Sample entropy during afternoon (12 PM to 6 PM) and SaEn Evening corresponds to Sample entropy during evening (6 PM to 12 AM).

# **Personalized relapse prediction framework’s performance across gender and age groups**

We assessed the differences in performances of the framework across sex and age ranges (18 - 44 years and 45-64 years) and the results are generalizable across comparisons in two studies. The median balanced accuracy of the framework was 71% (interquartile range: 70% - 75%) with median false alarm rate of 22.2% (interquartile range: 20.3% - 25.2%) leading to a median provider burden of 2.35 alarm/patient/year (interquartile range: 2.15 - 2.65) with a reduced patient burden (median: 50% [interquartile range: 44% - 53.2%]) requiring only 50% of the self-reported assessments compared to completing surveys on a standardized periodic basis (every week in this case). The detailed results are provided in **Tables S3-S6**.

**Table S3.** Relapse prediction framework performance on unseen test data, provider burden, and patient burden for the age range 18-44 years

|  | **Prediction performance** | | | | | | | | | |
| --- | --- | --- | --- | --- | --- | --- | --- | --- | --- | --- |
| **Dataset** | **Framework** | **SEN** | **SPEC** | **BAC** | | **PPV** | **NPV** | **FPR** | | **FAR** (%, per patient-year) |
| **OBSERVEMDD** | Active | 61.9 | 71.6 | 66.8 | | 8.0 | 97.9 | 28.4 | | 27.3, 2.0 |
|  | Passive + Active | 52.4 | 85.0 | 68.7 | | 12.2 | 97.8 | 15.0 | | 14.5, 1.4 |
| **CBN-WELL** | Active | 100.0 | 55.7 | 77.9 | | 18.4 | 100 | 44.3 | | 40.3, 3.3 |
|  | Passive + Active | 71.4 | 68.6 | 70.0 | | 18.5 | 96.0 | 31.4 | | 28.6, 2.7 |
|  | **Provider burden** | | | | | | | | | |
| **Dataset** | **Framework** | **Total number of patient days of observation** | | | **Total number of preemptive visits** | | | | **Provider burden**  **(per patient-year)** | |
| **OBSERVEMDD** | Active | 26581 | | | 162 | | | | 2.2 | |
|  | Passive + Active | 20278 | | | 90 | | | | 1.6 | |
| **CBN-WELL** | Active | 3397 | | | 38 | | | | 4.1 | |
|  | Passive + Active | 2986 | | | 27 | | | | 3.3 | |
|  | **Patient burden** | | | | | | | | | |
| **Dataset** | **Framework** | **Total number of self-reports assessments required (%)** | | | | | | | | |
| **OBSERVEMDD** | Active | 3100 (100) | | | | | | | | |
|  | Passive + Active | 1388 (44.8) | | | | | | | | |
| **CBN-WELL** | Active | 390 (100) | | | | | | | | |
|  | Passive + Active | 213 (54.6) | | | | | | | | |

BAC, balanced accuracy; FAR, false alarm rate; FPR, false positive rate; NPV, negative predictive value; PPV, positive predictive value; SEN, sensitivity; SPEC, specificity.

Provider burden was estimated based on the total number of preemptive visits (True Positive + False Positives) to the total number of patient days of observation; for instance, Patient burden was estimated based on the total number of self-report assessments required to be completed by the patient.

Since the “Active” framework was based on self-reports collected on a weekly basis, we defined it as the maximal patient burden (100%) and estimated the relative patient burden in “Active + Passive” framework as a fraction of the total self-reports used in that framework.

Number of relapse segments and non-relapse segments is 21 and 525 from 110 patients (OBSERVEMDD) and 7 and 70 from 30 patients (CBN-WELL). The observed prevalence rate of relapse at the population level and clinical site level is 25.45 % (82/100) and 3.85 % (21/546) for OBSERVEMDD and 23.33 % (7/30) and 9.1 % (7/77) for CBN-WELL respectively.

**Table S4.** Relapse prediction framework performance on unseen test data, provider burden, and patient burden for the age range 45-64 years

|  | **Prediction performance** | | | | | | | | | |
| --- | --- | --- | --- | --- | --- | --- | --- | --- | --- | --- |
| **Dataset** | **Framework** | **SEN** | **SPEC** | **BAC** | | **PPV** | **NPV** | | **FPR** | **FAR** (%, per patient-year) |
| **OBSERVEMDD** | Active | 86.2 | 65.4 | 75.8 | | 9.6 | 99.1 | | 34.6 | 33.2, 2.6 |
|  | Passive + Active | 75.9 | 77.5 | 76.7 | | 12.6 | 98.7 | | 22.5 | 21.6, 2.1 |
| **CBN-WELL** | Active | 66.7 | 63.2 | 64.9 | | 6.7 | 98.0 | | 36.8 | 35.4, 2.7 |
|  | Passive + Active | 66.7 | 75.0 | 70.8 | | 9.5 | 98.3 | | 25.0 | 24.1, 2.0 |
|  | **Provider burden** | | | | | | | | | |
| **Dataset** | **Framework** | **Total number of patient days of observation** | | | **Total number of preemptive visits** | | | **Provider burden**  **(per patient-year)** | | |
| **OBSERVEMDD** | Active | 33025 | | | 260 | | | 2.9 | | |
|  | Passive + Active | 27100 | | | 175 | | | 2.4 | | |
| **CBN-WELL** | Active | 3799 | | | 30 | | | 2.9 | | |
|  | Passive + Active | 3501 | | | 21 | | | 2.2 | | |
|  | **Patient burden** | | | | | | | | | |
| **Dataset** | **Framework** | **Total number of self-reports assessments required (%)** | | | | | | | | |
| **OBSERVEMDD** | Active | 3834 (100) | | | | | | | | |
|  | Passive + Active | 1852 (48.3) | | | | | | | | |
| **CBN-WELL** | Active | 448 (100) | | | | | | | | |
|  | Passive + Active | 271 (60.5) | | | | | | | | |

BAC, balanced accuracy; FAR, false alarm rate; FPR, false positive rate; NPV, negative predictive value; PPV, positive predictive value; SEN, sensitivity; SPEC, specificity.

Provider burden was estimated based on the total number of preemptive visits (True Positive + False Positives) to the total number of patient days of observation; for instance, Patient burden was estimated based on the total number of self-report assessments required to be completed by the patient.

Since the “Active” framework was based on self-reports collected on a weekly basis, we defined it as the maximal patient burden (100%) and estimated the relative patient burden in “Active + Passive” framework as a fraction of the total self-reports used in that framework.

Number of relapse segments and non-relapse segments is 29 and 679 from 117 patients (OBSERVEMDD) and 3 and 76 from 20 patients (CBN-WELL). The observed prevalence rate of relapse at the population level and clinical site level is 32.5 % (38/117) and 4.09 % (29/708) for OBSERVEMDD and 35 % (7/20) and 3.8 % (3/79) for CBN-WELL respectively.

**Table S5.** Relapse prediction framework performance on unseen test data, provider burden, and patient burden for male patients

|  | **Prediction performance** | | | | | | | | | | |
| --- | --- | --- | --- | --- | --- | --- | --- | --- | --- | --- | --- |
| **Dataset** | **Framework** | **SEN** | **SPEC** | **BAC** | | **PPV** | **NPV** | **FPR** | | | **FAR** (%, per patient-year) |
| **OBSERVEMDD** | Active | 64.3 | 67.3 | 65.8 | | 6.8 | 98.1 | 32.7 | | | 31.5, 2.4 |
|  | Passive + Active | 64.3 | 77.9 | 71.1 | | 9.8 | 98.3 | 22.1 | | | 21.3, 2.0 |
| **CBN-WELL** | Active | 100.0 | 61.3 | 80.6 | | 14.3 | 100.0 | 38.7 | | | 36.4, 2.9 |
|  | Passive + Active | 75.0 | 75.8 | 75.4 | | 16.7 | 97.9 | 24.2 | | | 22.7, 2.1 |
|  | **Provider burden** | | | | | | | | | | |
| **Dataset** | **Framework** | **Total number of patient days of observation** | | | **Total number of preemptive visits** | | | | **Provider burden**  **(per patient-year)** | | |
| **OBSERVEMDD** | Active | 18428 | | | 132 | | | | | 2.6 | |
|  | Passive + Active | 14870 | | | 92 | | | | | 2.3 | |
| **CBN-WELL** | Active | 2970 | | | 28 | | | | | 3.4 | |
|  | Passive + Active | 2566 | | | 18 | | | | | 2.6 | |
|  | **Patient burden** | | | | | | | | | | |
| **Dataset** | **Framework** | **Total number of self-reports assessments required (%)** | | | | | | | | | |
| **OBSERVEMDD** | Active | 2136 (100) | | | | | | | | | |
|  | Passive + Active | 984 (46.1) | | | | | | | | | |
| **CBN-WELL** | Active | 342 (100) | | | | | | | | | |
|  | Passive + Active | 175 (51.1) | | | | | | | | | |

BAC, balanced accuracy; FAR, false alarm rate; FPR, false positive rate; NPV, negative predictive value; PPV, positive predictive value; SEN, sensitivity; SPEC, specificity.

Provider burden was estimated based on the total number of preemptive visits (True Positive + False Positives) to the total number of patient days of observation; for instance, Patient burden was estimated based on the total number of self-report assessments required to be completed by the patient.

Since the “Active” framework was based on self-reports collected on a weekly basis, we defined it as the maximal patient burden (100%) and estimated the relative patient burden in “Active + Passive” framework as a fraction of the total self-reports used in that framework.

Number of relapse segments and non-relapse segments is 14 and 376 from 66 patients (OBSERVEMDD) and 4 and 62 from 19 patients (CBN-WELL). The observed prevalence rate of relapse at the population level and clinical site level is 27.2 % (18/66) and 3.6 % (14/390) for OBSERVEMDD and 26.3 % (5/19) and 6.1 % (4/66) for CBN-WELL respectively.

**Table S6.** Relapse prediction framework performance on unseen test data, provider burden, and patient burden for female patients

|  | **Prediction performance** | | | | | | | | | | |
| --- | --- | --- | --- | --- | --- | --- | --- | --- | --- | --- | --- |
| **Dataset** | **Framework** | **SEN** | **SPEC** | | **BAC** | | **PPV** | **NPV** | | **FPR** | **FAR** (%, per patient-year) |
| **OBSERVEMDD** | Active | 80.6 | 68.5 | | 74.5 | | 10.0 | 98.8 | | 31.5 | 30.2, 2.3 |
|  | Passive + Active | 66.7 | 82.0 | | 74.3 | | 13.9 | 98.3 | | 18.0 | 17.2, 1.7 |
| **CBN-WELL** | Active | 83.3 | 58.3 | | 70.8 | | 12.5 | 98.0 | | 41.7 | 38.9, 3.0 |
|  | Passive + Active | 66.7 | 69.0 | | 67.9 | | 13.3 | 96.7 | | 31.0 | 28.9, 2.4 |
|  | **Provider burden** | | | | | | | | | | |
| **Dataset** | **Framework** | **Total number of patient days of observation** | | | | **Total number of preemptive visits** | | | **Provider burden**  **(per patient-year)** | | |
| **OBSERVEMDD** | Active | 41178 | | | | 290 | | | 2.6 | | |
|  | Passive + Active | 32508 | | | | 173 | | | 1.9 | | |
| **CBN-WELL** | Active | 4226 | | | | 40 | | | 3.5 | | |
|  | Passive + Active | 3921 | | | | 30 | | | 2.8 | | |
|  | **Patient burden** | | | | | | | | | | |
| **Dataset** | **Framework** | **Total number of self-reports assessments required (%)** | | | | | | | | | |
| **OBSERVEMDD** | Active |  | | 4798 (100) | | | | | | | |
|  | Passive + Active |  | | 2256 (47) | | | | | | | |
| **CBN-WELL** | Active |  | | 496 (100) | | | | | | | |
|  | Passive + Active |  | | 309 (62) | | | | | | | |

BAC, balanced accuracy; FAR, false alarm rate; FPR, false positive rate; NPV, negative predictive value; PPV, positive predictive value; SEN, sensitivity; SPEC, specificity.

Provider burden was estimated based on the total number of preemptive visits (True Positive + False Positives) to the total number of patient days of observation; for instance, Patient burden was estimated based on the total number of self-report assessments required to be completed by the patient.

Since the “Active” framework was based on self-reports collected on a weekly basis, we defined it as the maximal patient burden (100%) and estimated the relative patient burden in “Active + Passive” framework as a fraction of the total self-reports used in that framework.

Number of relapse segments and non-relapse segments is 36 and 828 from 161 patients (OBSERVEMDD) and 6 and 84 from 31 patients (CBN-WELL). The observed prevalence rate of relapse at the population level and clinical site level is 29.8 % (48/161) and 4.2 % (36/864) for OBSERVEMDD and 29 % (9/31) and 6.6 % (6/90) for CBN-WELL respectively.

# **References**

1. Montgomery SA, Åsberg M. A new depression scale designed to be sensitive to change. The British journal of psychiatry*.* 1979;134:382-9.

2. Hundman K, Constantinou V, Laporte C, Colwell I, Soderstrom T. in *In* *Proceedings of the 24^th^ ACM SIGKDD international conference on knowledge discovery & data mining.* Detecting spacecraft anomalies using lstms and nonparametric dynamic thresholding. 387-95 (2018).

3. Li P, Lim ASP, Gao L, Hu C, Yu L, Bennett DA *et al.* More random motor activity fluctuations predict incident frailty, disability, and mortality. Sci Transl Med*.* 2019;11.

4. Hauge ER, Berle JO, Oedegaard KJ, Holsten F, Fasmer OB. Nonlinear analysis of motor activity shows differences between schizophrenia and depression: a study using Fourier analysis and sample entropy. PLoS One*.* 2011;6:e16291.

5. Li P, Yu L, Yang J, Lo MT, Hu C, Buchman AS *et al.* Interaction between the progression of Alzheimer's disease and fractal degradation. Neurobiol Aging*.* 2019;83:21-30.

6. Bates D, Maechler M, Bolker B, Walker S. lme4: Linear mixed-effects models using Eigen and S4. R package version 1.1-7. (2014).

7. Vincent, A.B. marginaleffects: Predictions, Comparisons, Slopes, Marginal Means, and Hypothesis Tests. R package version 0.11.0. (2023).
